# Easy-To-Access Quinolone Derivatives Exhibiting Antibacterial and Anti-Parasitic Activities

**DOI:** 10.3390/molecules26041141

**Published:** 2021-02-20

**Authors:** Richard M. Beteck, Audrey Jordaan, Ronnett Seldon, Dustin Laming, Heinrich C. Hoppe, Digby F. Warner, Setshaba D. Khanye

**Affiliations:** 1 Centre of Excellence for Pharmaceutical Sciences, North-West University, Potchefstroom 2520, South Africa; 2 SAMRC/NHLS/UCT Molecular Mycobacteriology Research Unit, Department of Pathology, Faculty of Health Sciences, Institute of Infectious Disease and Molecular Medicine, University of Cape Town, Cape Town 7925, South Africa; audrey.jordaan@uct.ac.za (A.J.); Digby.Warner@uct.ac.za (D.F.W.); 3 SAMRC Drug Discovery and Development Research Unit, University of Cape Town, Cape Town 7700, South Africa; ronnett.seldon@uct.ac.za; 4 Centre for Chemico- and Biomedicinal Research, Rhodes University, Makhanda 6140, South Africa; dustinlaming89@gmail.com (D.L.); h.hoppe@ru.ac.za (H.C.H.); 5 Department of Biochemistry and Microbiology, Faculty of Science, Rhodes University, Makhanda 6140, South Africa; 6 Wellcome Centre for Infectious Diseases Research in Africa, University of Cape Town, Cape Town 7925, South Africa; 7 Division of Pharmaceutical Chemistry, Faculty of Pharmacy, Rhodes University, Makhanda 6140, South Africa

**Keywords:** anti-*Mtb*, human African trypanosomiasis, quinolones, malaria, ESKAPE pathogens

## Abstract

The cell wall of *Mycobacterium tuberculosis* (*Mtb*) has a unique structural organisation, comprising a high lipid content mixed with polysaccharides. This makes cell wall a formidable barrier impermeable to hydrophilic agents. In addition, during host infection, *Mtb* resides in macrophages within avascular necrotic granulomas and cavities, which shield the bacterium from the action of most antibiotics. To overcome these protective barriers, a new class of anti-TB agents exhibiting lipophilic character have been recommended by various reports in literature. Herein, a series of lipophilic heterocyclic quinolone compounds was synthesised and evaluated in vitro against pMSp12::GFP strain of Mtb, two protozoan parasites (*Plasmodium falciparum* and *Trypanosoma brucei brucei*) and against ESKAPE pathogens. The resultant compounds exhibited varied anti-*Mtb* activity with MIC_90_ values in the range of 0.24–31 µM. Cross-screening against *P. falciparum* and *T.b. brucei*, identified several compounds with antiprotozoal activities in the range of 0.4–20 µM. Compounds were generally inactive against ESKAPE pathogens, with only compounds **8c**, **8g** and **13** exhibiting moderate to poor activity against *S. aureus* and *A. baumannii*.

## 1. Introduction

*Mycobacterium tuberculosis* (*Mtb*) is a pathogenic bacterium that has been reported to have co-evolved with humans over thousands of years [1]. *Mtb* causes TB [2], a disease that is currently the leading cause of death globally. An estimated 10.0 million people developed TB in 2018, of which approximately 1.5 million people lost their lives [3]. TB disproportionally affects low to middle-income countries [4]. For example, in 2017, 44% of TB cases occurred in south Asia and 25% in Africa, as compared to 2.7% cases in Europe, and 2.8% in the Americas [3]. The high TB prevalence in resource poor countries is partly fuelled by malnutrition [5] and co-infection with HIV/AIDS [6]. The treatment and management of TB face several lacunas [7]. These include reduced efficacy of most drugs in the first line treatment regimen (MIC 15 µM for ethambutol) [8], long treatment period of 6–24 months and the emergence and spread of strains of *Mtb* which are resistant to anti-TB drugs rifampicin and/or isoniazid [9,10]. As a result of drug resistance, 3.5% of new TB cases in 2017 could not be treated using rifampicin and/or isoniazid [3,11]. Therefore, there is a pressing need to engage in TB drug discovery research to explore and identify new compounds which are likely to counteract drug resistance.

The *Mtb* cell wall has a unique structural organisation which differs from that of most bacteria: the high lipid content mixed with polysaccharides makes it a formidable barrier almost impermeable to hydrophilic agents [12,13]. This presents a significant innate obstacle to most drugs/hit compounds which must penetrate the cell wall to effectively access their targets within *Mtb* [14]. In addition to forming a protective barrier, the cell wall is a host to several druggable proteins, some of which execute life sustaining metabolic processes such as energy production, synthesis of cell wall components as well as transport of nutrients and waste [15]. An additional barrier to drug penetration is the granuloma, which arises as a direct consequence of the host immune system trying to contain the pathogenic bacilli [16]. Characteristically, the granuloma is a lesion formed through the aggregation of several activated immune cells. At the centre of this structure is mostly bacilli infected macrophages. These macrophages eventually die, forming necrotic granulomas wherein the centre is filled with cheese-like cellular debris (caseum) [17,18,19].

**Figure 1:**
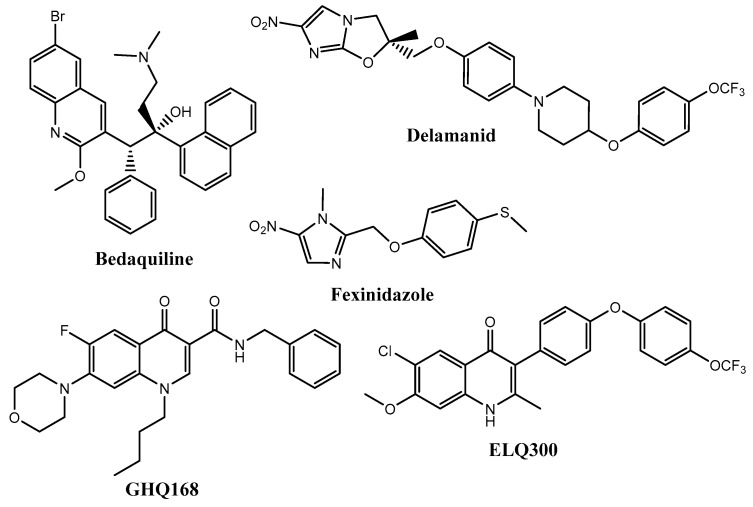
Structures of anti-TB, antitrypanosomal and antimalarial agents.

The structure of the *Mtb* cell wall, coupled with the microenvironments arising as a direct consequence of TB pathophysiology, requires new anti-TB agents with physiochemical properties that deviates from Lipinski’s rule of five [20]. One such desirable property is lipophilicity [21]. This is evident in bedaquiline and delamanid (Figure 1), the most recently approved anti-TB agents, both of which have high lipophilicity and molecular weight greater than 500 Da [22,23]. Moreover, it has been observed that anti-TB activity within a compound series increases with increased lipophilicity [24].

Besides TB, malaria is another major tropical infection [25]. This disease, caused principally by *Plasmodium falciparium* [26], affected 219 million people and led to 435000 deaths in 2018 [27]. More than 90% of malaria cases and deaths occur in sub-Saharan Africa. The treatment and prevention of malaria is traditionally dependent on the use of chemotherapeutic agents [28], which are often used in combinations [29]. After a decade of dwindling malaria cases, current statistics shows an increase in malarial incidence. For-example, in the period 2016 to 2017, the number of malarial cases increased by 2 million [30]. This increase is partly due to the emergence and spread of multi-drug resistant *P. falciparium* strains [31], and places urgent need for the discovery of new anti-malarial agents.

Similarly, sleeping sickness, caused by *Trypanosoma* species, is an incapacitating disease that affects only the rural communities in sub-Saharan Africa [32]. In 2018, roughly 1000 new cases of the disease were reported [33], and an estimated 70 million people are currently living in areas to which the disease is endemic [34]. In addition to the issue of drug resistant parasites [35], there are limited treatment alternatives for this disease and all clinically proven drugs for the treatment of sleeping sickness suffer from various limitations including poor drug-like properties [36]. For-example, patients on melarsoprol often die from the toxicity of the drug than the disease [37]. Despite these shortcomings, the drug development pipeline for sleeping sickness is empty with just only two candidates under development [38]. Recently, a partnership between Sanofi and Drugs for Neglected Diseases initiative (DND*i*) led to the approval of fexinidazole (Figure 1) as alternative therapeutic tool for targeting both stages (1 and 2) of trypanosomiasis [39]. Nevertheless, the emergence and growing concerns of resistance remain the basis to search for new compounds with the potential to cure sleeping sickness.

Previously, we reported several hit compounds containing a quinolone nucleus showing good biological activities against *Mtb*, *P. falciparum* and *Trypanosoma brucei brucei* [40,41]. More importantly, the quinolone nucleus is an important framework utilised to design a diverse class of novel compounds exhibiting a myriad of biological activities [42]. In recent years, several studies have revealed that in some developing countries there has been an increasing overlap between TB and infectious parasitic diseases in similar geographic distribution [43,44]. Considering a number of reported TB cases in regions where there is a high burden of infectious protozoan parasites, there is a likelihood of TB, malaria and trypanosomiasis co-infections [45,46]. Herein, we are reporting the synthesis of easily accessible lipophilic quinolone compounds and their in vitro activities against *Mtb* and other pathogens responsible for related tropical parasitic diseases.

## 2. Results and Discussions

### 2.1. Chemistry

**Scheme 1:**
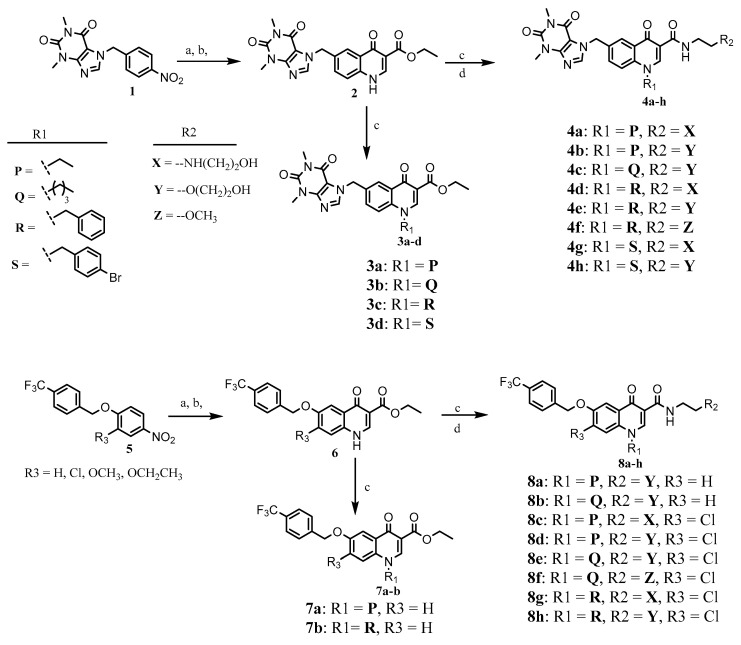
General synthesis of compounds **3a**–**d**, **4a**–**h**, **7a**–**b** and **8a**–**h**. *Reagents and conditions*: (a) Fe powder (1 equiv), NH_4_Cl (1 equiv), EtOH, reflux 12 h; (b) (i) diethyl ethoxymethylenemalonate (2 equiv), reflux 12 h, (ii) Dowtherm A, 245–250 °C, 5 min; (c) K_2_CO_3_ (1 equiv), CHCl_3_/THF (3:1), alkyl/arylhalide (1.2 equiv), reflux 12 h; (d) Amine (5 equiv), DBU (1.2 equiv), CHCl_3_, reflux 12 h.

**Scheme 2:**
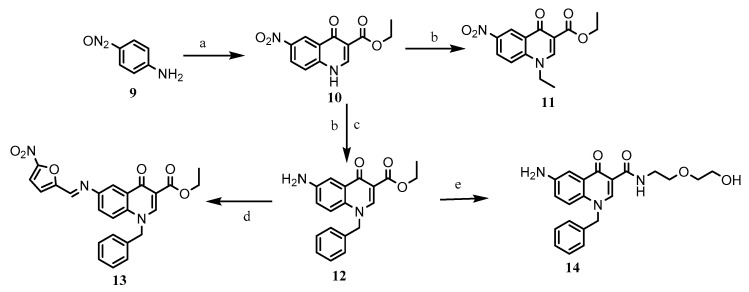
Synthesis of compounds **11**–**14**. *Reagents and conditions*: (a) (i) Diethyl ethoxymethylenemalonate (2 equiv), ACN, reflux 12 h; (ii) Eaton’s reagent, 70–80 °C, N_2_, overnight; (b) K_2_CO_3_ (1 equiv), CHCl_3_/THF (3:1), alkyl/arylhalide (1.2 equiv), reflux 12 h; (c) Fe powder (1 equiv), NH_4_Cl (1 equiv), EtOH reflux 12 h; (d) 5-nitrofurfural (1.2 equiv), AcOH (cat), EtOH, reflux overnight; (e) Amine (5 equiv), DBU (1.2 equiv), CHCl_3_, reflux 12 h.

**Scheme 3:**
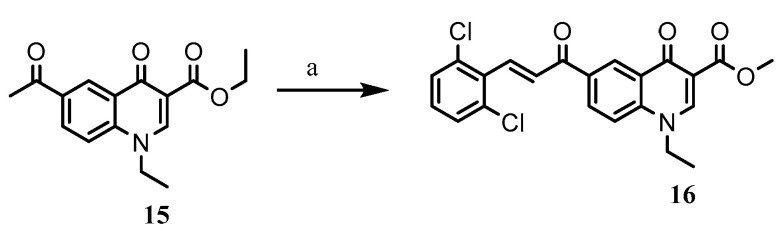
Synthesis of compounds **15**. *Reagents and conditions*: (a) NaOH, MeOH, 2,6-dichlorobenzaldehyde, r.t., 12 h.

Target compounds were easily accessed following the simple synthetic transformations presented in Scheme 1, Scheme 2 and Scheme 3 below. Compound **1** was obtained through alkylation of theophylline by *p*-nitrobenzyl bromide in acetone at room temperature [47]. Subsequently, reduction of the nitro group using iron powder in the presence of NH_4_Cl as the hydrogen source afforded an amino intermediate, which was condensed with diethyl ethoxyethylenemalonate and subjected to thermal cyclization at 245–250 °C for five minutes in Dowtherm A to give a quinolone **2** (Scheme 1). Selective alkylation of the quinolone **2** through the secondary amine with alkyl/aryl halides using a solvent mixture of chloroform/tetrahydrofurn (3:1) and K_2_CO_3_ as the base afforded target compounds **3a**–**d** with yields in the range 50–70% [40]. Treatment of **3a**–**d** with appropriate aliphatic primary amines following a previously reported procedure [48] afforded target compounds **4a**–**h** in 30–60% yields. Fragment **5** was obtained via *O*-alkylation of substitute 4-nitrophenenols with 4-(trifluoromethyl)benzyl bromide in refluxing acetone using K_2_CO_3_ as the base. Compounds **6** leading to **7a**–**b** and **8a**–**h** were obtained using the same procedure discussed for compound **2** leading to **3a**–**d** and compounds **4a**–**h**.

Quinolone analogues bearing an amino, or nitro group attached to the benzenoid ring were also synthesised according to Scheme 2. Briefly, *p*-nitroaniline (**9**) was treated with diethyl ethoxymethylene malonate in refluxing acetonitrile to afford the intermediate diethyl (4-nitrophenylaminomethylene)malonate, subsequently cyclized using Eaton’s reagent at 70–80 °C overnight under nitrogen atmosphere to afford the key starting compound **10** in 80% yield [49]. *N*-alkylation of **10** with EtBr gave target compound **11** in 70% yield. Alternatively, compound **10** was alkylated with BnBr, and subsequently treated with reduced iron powder and NH_4_Cl to afford target compound **12** in 65% yield. The amino group in **12** provided an easy handle for further derivatisation of the benzenoid ring. For-example, the -NH_2_ moiety reacted with 5-nitrofurfural through a Schiff base condensation to generate compound **13** in 80% yield. It is noteworthy to point out that while additional analogous compounds to **13** were pursued, they could not be reported since they were practically insoluble in common NMR solvents (CDCl_3_ or DMSO-*d_6_*). Treatment of **12** with amino-alcohol in the presence of 1,8-diazabicyclo[5.4.0]undec-7-ene (DBU) as a base afforded compound **14** in 40% yield.

As part of exploring structure-activity relationship (SAR), compound **15** (Scheme 3) was obtained following previously reported protocols [38]. Compound **15** underwent concurrent aldol condensation and *trans* esterification following treatment with NaOH and 2,6-dichlorobenzaldehyde in MeOH to afford compound **16** in 40% yield. All target compounds were confirmed using ^1^H-, ^13^C-NMR and HRMS analyses (see Supplementary Materials).

### 2.2. In Vitro Biological Evaluation

All target compounds were screened in vitro against the drug susceptible pMSp12::GFP strain of *Mtb*, the 3D7 chloroquine sensitive strain of *P. falciparum*, the 427 strain of *T. b. brucei* and against five bacteria dubbed ESKAPE pathogens. These compounds were also tested in vitro against human embryonic kidney cells (HEK293) for cytotoxicity.

#### 2.2.1. Anti-Mtb Activity

For *Mtb*, the compounds were evaluated via broth micro dilution assay in standard Middlebrook 7H9 base medium. Anti-*Mtb* activity was measured after fourteen days incubation and the activity of compounds showing anti-mycobacterial activity reported in Table 1 as MIC_90_, which is the minimum compound concentration required to inhibit 90% of Mtb growth. Rifampicin was included as a positive control. The majority of compounds in this library presented moderate to poor anti-Mtb activity, however compounds **8c, 8g**, and **16** exhibited low micro-molar activity with MIC_90_ values in the range of 0.9–2.0 µM. The in vitro biological data suggest that lipophilicity and/or structural variation influences anti-Mtb activity. Looking at the activity profile of the compound sub-series **3a**–**d** and **4a**–**h**, it could generally be inferred that increasing lipophilicity of N1 substituents seems to favour anti-*Mtb* activity. For example, compound **3a** and **3b** bearing ethyl and butyl substituents at N1, respectively, showed poor anti-*Mtb* activity (MIC_90_ > 125 µM) compared to the activity of 15.6 µM for **3d** which bears a more lipophilic *p*-bromobenzyl substituent. This trend is also observed when comparing **4d** (MIC_90_ 7.9 µM) having a benzyl substituent with **4a** (MIC_90_ ˃ 125 µM) having an ethyl substituent at N1. Compound **4g** (MIC_90_ 7.9 µM, ClogP 1.2) and 4h (MIC_90_ ˃ 125 µM, ClogP 1.3) have comparable ClogP values, but their anti-*Mtb* activity vary greatly. This suggests that structural properties, such as the presence of a basic secondary amine, rather than lipophilicity seem to influence anti-*Mtb* activity. Activity differences between compound **3d** (MIC_90_ 15.6 µM, ClogP 3.3) and its derivative **4g** (MIC_90_ 7.9 µM, ClogP 1.2) also showed no correlation with increasing lipophilicity mentioned above. Moving from **3d** to **4g,** anti-*Mtb* activity increases over two fold while lipophilicity decreases over 2.5 fold. This further confirms the influence of a basic secondary anime on enhancing anti-*Mtb* activity for this set of compounds.

In compounds **7a**–**b**, **8a**–**h**, the benzenoid ring is substituted with a lipophilic trifluoromethyl benzyloxy moiety and in some cases concurrently with a Cl atom. The biological data obtained for this sub-series indicates that the presence of an ethyl-3-carboxylate moiety as illustrated by **7a** and **7b** generally leads to compounds with no anti-*Mtb* activity, and that derivatisation to obtain *N*-[2-(2-hydroxyethoxy)ethyl]-3-carboxamides generally enhances activity. For example, compound **7b** bearing an ethyl-3-carboxylate is not active (MIC_90_ ˃ 125 µM), while its amide derivative **8h** shows moderate activity (MIC_90_ 16 µM). Looking at the activity profile of the compound **8c** (MIC_90_ 1.9 µM) and **8g** (MIC_90_ 0.9 µM), it could again be inferred that increasing lipophilicity of N1 substituents seems to favour anti-*Mtb* activity. Replacing the ethyl moiety at N1 position in compound **8c** with a benzyl moiety to afford compound **8g** led to a two-fold increase in anti-*Mtb*.

Compound **12** (*C*logP 2.3) with an -NH_2_ group attached to the benzenoid ring is devoid of anti-*Mtb* activity, while it’s derivative **13** shows anti-*Mtb* activity (MIC_90_ 8 µM, *C*logP 2.3). The observed activity could be attributed to increased lipophilicity and/or the presence of a nitrofuranyl moiety, which has been previously reported to enhance anti-*Mtb* activity [50]. Comparing compound **14** (MIC_90_ ˃ 125 µM) which bears only a polar -NH_2_ moiety on the benzenoid ring against compound **8h** (MIC_90_ 16 µM) which incorporates trifluoromethyl benzyloxy moiety and a Cl atom on the benzenoid ring example, demonstrate that increasing the lipophilicity of benzenoid substituents promote anti-*Mtb* activity.

#### 2.2.2. Antiplasmodial and Anti-Trypanosomal Activities

**Figure 2:**
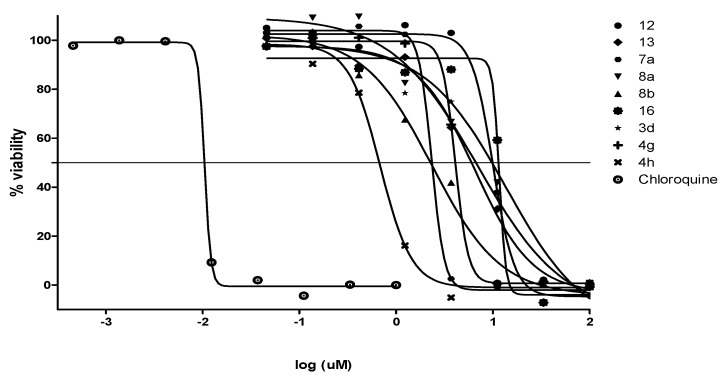
Dose-response curves of compounds active against *P. falciparum* compared with chloroquine.

**Figure 3:**
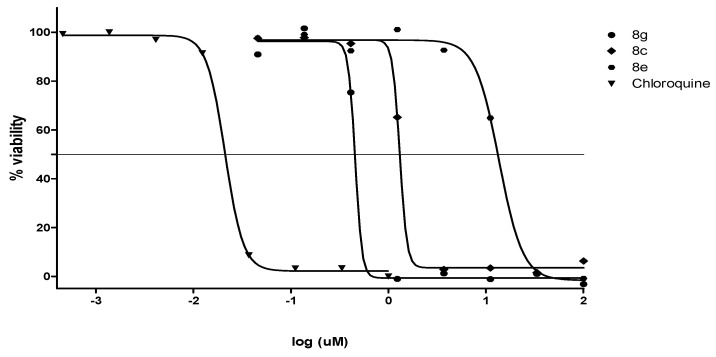
Dose-response curves of compounds active against *P. falciparum* compared with chloroquine.

All target compounds were cross-screened against the protozoan parasites *P. falciparum* and *T. b. brucei*. Chloroquine (CQ) and pentamidine (PE) were used as standard drugs. These compounds demonstrated varied antiplasmodial activity in the range of 0.4–14 µM (Table 1). Compound **4h** and **8g** emerged as the most potent compounds with IC_50_ values of 0.68 µM and 0.4 µM, respectively against the CQ sensitive strain (3D7) of *P. falciparum*. Compounds **4g**, **7a**, **8a**, **8b**, **8c** and **13** exhibited low micro molar activity of <10 µM against 3D7, while compounds **3d**, **8e**, **12** and **16** demonstrated poor antiplasmodial activity in the range of 10–14 µM. The dose-response curves of antiplasmodial active compounds compared against CQ is presented in Figure 2 and Figure 3.

Comparing compound **4g** (3D7 IC_50_, 4.1 µM) against compound **4d** (3D7 IC_50_, ≥25 µM), and compound **4h** (3D7 IC_50_, 0.68 µM) against compound **4e** (3D7 IC_50_, ≥25 µM), which differ only on a Br moiety at the N1 substituent suggest that the presence of a bromine atom at this position enhances antiplasmodial activity. This could probably be due to the increased lipophilicity afforded by the bromine moiety. Compound **12** bearing an ethyl-3-carboxylate moiety at C3 position presented an IC_50_ value of 10 µM against 3D7. Modification of the ethyl-3-carboxylate moiety to generate amide derivatives as exemplified by compound **14** led to complete loss of antiplasmodial activity, while modifying the -NH_2_ group attached to the benzenoid ring generated compound **13** with increased activity (3D7 IC_50_, 6.5 µM). This observation suggests that ethyl-3-carboxylate moiety at C3 position seems to generally promote antiplasmodial activity. This trend is also noted when comparing compound **7a** (3D7 IC_50_, 2.3 µM) against its amide derivative **8a** (3D7 IC_50_, 6.8 µM). The apparent exception to this trend is compound **3d** (3D7 IC_50_, 13.6 µM), which demonstrated lesser antiplasmodial activity than it amide derivatives **4g** (3D7 IC_50_, 4.1 µM) and **4h** (3D7 IC_50_, 0.68 µM).

**Figure 4:**
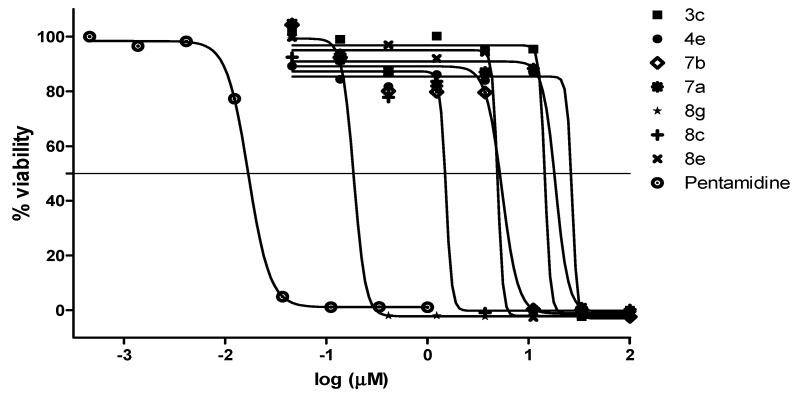
Dose-response curves of compounds active against *T.b. brucei* compared with pentamidine.

**Figure 5:**
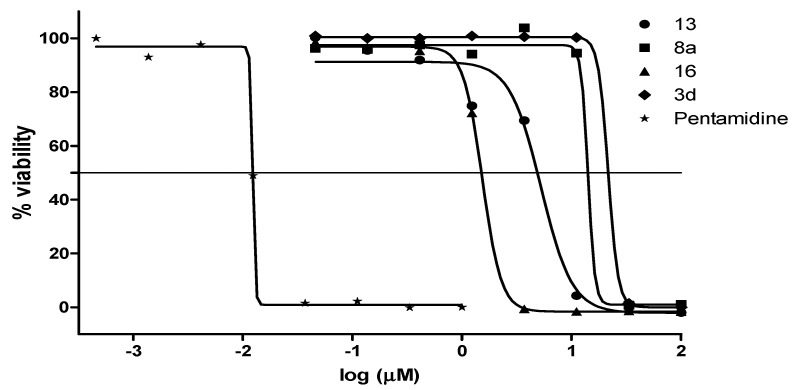
Dose-response curves of compounds active against *T.b. brucei* compared with pentamidine.

With respect to anti-trypanosomal activity, compounds **8c**, **8g** and **16** exhibited potent activity with IC_50_ values below 2 µM. The rest of the compounds in this study generally presented moderate to weak anti-trypanosomal activity with IC_50_ values in the range of 5–27 µM. The dose-response curves of compounds showing anti-trypanosomal activity compared against pentamidine is presented in Figure 4 and Figure 5.

#### 2.2.3. Antimicrobial Activities and Cytotoxicity Evaluation

Target compounds were also evaluated against ESKAPE (*E. faecium*, *A. baumanni*, *K. pneumoniae*, *P. aeruginosa*, *S. aureus*) pathogens for antibacterial activities using a broth microdilution method wherein colistin and vancomycin were used as standards for Gram-negative and Gram-positive bacteria, respectively. Compounds in these study were generally not active against ESKAPE pathogens, with only three compounds (**8c**, **8g**, **13**) showing moderate to weak, narrow spectrum activity against selected bacteria. Compound **8c** exhibited MIC values of 16 µg/mL and 32 µg/mL against *S. aureus* (MRSA) and *A. baumanni*, respectively. Compound **8g** demonstrated a weak activity of 32 µg/mL against *S. aureus* (MRSA), while compound **13** presented a moderated activity of 16 µg/mL against *A. baumanni*. The difference in anti-mycobacterial activity and antibacterial activities observed for these compounds could be attributed to the high lipid content of *Mtb* cell wall which is likely to favour diffusion of this lipophilic molecules into its cell wall- a compartment that host essential drug targets peculiar to *Mtb*.

All compounds were also evaluated against Human Embryonic Kidney cells (HEK293) for cytotoxicity and tamoxifen was included as a reference in the assay. HEK293 cells were incubated with 32 µg/mL of quinolone derivatives and the percent inhibition of the cells measured. By the protocol used, compounds exhibiting ≥ 50% HEK293 cells inhibition were considered cytotoxic and subjected to further dose-response analyses and eventual CC50 value generation. All compounds deployed in this study exhibited < 20% inhibition of HEK293 cells at 32 µg/mL and are hence not cytotoxic at the minimum concentration required to exhibit anti-mycobacterial and/or anti-parasitic activities.

### 2.3. Lipophilicity

Lipophilicity is a central physicochemical parameter in drug design of small drug molecules, and it has direct influence on several other ADMET properties of a compound [51]. For example, high lipophilicity often results in poor aqueous solubility, increased in vitro promiscuity, enhanced drug metabolism and high volume of distribution. Low lipophilicity negatively affects permeability and results in increased renal clearance [52]. Following Lipinski’s rule of five, it is generally accepted that drug-like molecules should exhibit a lipophilicity (*log P*) value in the range of 1 to 5 [53]. However, this rule seems to fall outside the space of anti-*Mtb* agents, most of which demonstrate *log P* values greater than 5 [22,54,55]. Despite their time consuming and labour intensive nature [56], the shake-flask and potentiometric titration methods are commonly deployed experimental techniques for the determination of *log P* values [57]. Considering the importance of *log P* values in drug design and the large number of compounds often involved in hit ‘hunting’, the common and accepted practice has been to generate calculated lipophilicity (*Clog P*) using computational tools [58,59]. Although it has been observed that *Clog P* values generated from different computer programs differ from the experimentally determine *log P*, often with a difference of more than 0.5 units; there however exist a strong correlation (r > 0.6) between computer generated *Clog P* values and experimentally determine *log P*. This evidence makes *Clog P* values a usable guide in early hit finding [60]. Herein, we generated *Clog P* values using ACD/Chemsketch freeware version 12.0. The compounds showed a diversity of *Clog P* values varying in the range of 0.4 to 5.9.

## 3. Materials and Methods

### 3.1. Chemistry: Materials and Instrumentation

Fine chemicals and bulk solvents were purchased from various chemical suppliers and were used as supplied. Melting points were determined with a B-545 melting point instrument (Büchi, Johannesburg, South Africa) and are reported as obtained. Reactions progress was monitored by thin layer chromatography (TLC) using 60 F_254_ silica gel plates (Merck, Johannesburg, South Africa) supported on aluminium and components were visualised under ultraviolet light (254 nm). Flash column chromatography was carried out using Merck Kieselgel 60 Å: 70–230 (0.068–0.2 mm) silica gel mesh as the stationary phase. ^1^H- and ^13^C-NMR spectra were recorded on a Biospin 400 MHz spectrometer (Bruker, Ettlingen, Germany). The chemical shifts (δ) values are given in parts per million (ppm) and are internally referenced to residual solvents peak (DMSO-*d_6_*; 2.50 ppm ^1^H, 39.51 ppm ^13^C, CDCl_3_; 7.2 ppm ^1^H, 77.6 ppm ^13^C). Peak multiplicities are abbreviated as follows: s (singlet), d (doublet), t (triplet), q (quartet) and m (multiplet) while coupling constants (*J*) are reported in Hz. NMR data were processed using MestReNova Software version 5.3.2-4936. Purity was determined by HPLC and all compounds were confirmed to have purity >95%. The chromatographic system consisted of an Agilent HP1100 LC-MSD (Agilent, Santa Clara, CA, USA), which is equipped with a quaternary pump, in-line degasser, DAD detector, 1100 MSD and ChemStation for collection and analysis of data. A ZORBAX Elipse Plus C18 4.6 i.d. × 150 mm × 5 µm column was used for reversed-phase HPLC analysis. The high-resolution mass spectrometry data (HRS-MS) of final compounds was recorded on a Waters (Milford, MA, USA) Synapt G2 quadrupole time-of-flight (QTOF) mass spectrometer (Stellenbosch University, Stellenbosch, South Africa) using electrospray ionization (ESI) in the positive ionization mode.

### 3.2. Synthesis of Target Compounds

#### 3.2.1. *N*-alkylation

To a 200 mL round bottom flask containing 30 mL of CHCl_3_/THF (2:1, *v/v*) mixture was added 1 g of 4-oxo-3-carboxy quinolones, K_2_CO_3_ (5 eq.), alkyl/aryl halide (1.2 eq.) and the resultant mixture was allowed to reflux for 12 h. Upon reaction completion as indicated by TLC, the mixture was filtered off K_2_CO_3_, and the filtrate evaporated to dryness to obtain a crude *N*-alkylated 4-oxo-3-carboxy quinolones, which was purified through silica gel column chromatography using CH_2_Cl_2_/MeOH (10:1) as the mobile phase. *N*-alkylated 4-oxo-3-carboxy quinolones **3a**–**d**, **7a**–**b**, **11**, **12** were obtained in 50–70% yields following this procedure.

#### 3.2.2. Ester Aminolysis

A mixture of *N*-alkylated 4-oxo-3-carboxy quinolones (1 g, 1 eq.), DBU (1.2 eq.), an appropriate amine (5 eq.), and chloroform (15 mL) in a 100 mL round bottom flask was stirred under reflux for 24 h. Upon reaction completion as indicated by TLC, the mixture was evaporated to dryness and resultant crude subjected to silica gel column chromatography eluting with CH_2_Cl_2_/MeOH (10:1). Thereafter, fractions containing the desired product were combined, evaporated to dryness and recrystallised from ethanol to afford *N*-alkylated 4-oxo-1,4-dihydroquinolones-3-carboxamides **4a**–**h**, **8a**–**h**, **14** with yields in the 30–60%.

#### 3.2.3. Imine Formation

A 100 mL round bottom flask was charged with 20 mL of 95 % ethanol, 400 mg (1.2 mmol) of 12, few drops of glacial acetic acid and 263 mg (1.8 mmol, 1.5 eq.) of 5-nitrofurfural. The reaction mixture was stirred under reflux for 12 h. During the course of reaction, the product precipitated out and was filtered, washed twice with 10 mL portions of ethanol and dried to obtain 400 mg of target compound 13 in yield of 72%.

*(Ethyl 6-((1,3-dimethyl-2,6-dioxo-1,2,3,6-tetrahydro-7H-purin-7-yl)methyl)-1-ethyl-4-oxo-1,4-dihydroquinolone-3-carboxylate)*, (**3a**). White powder, yield 50%, m.p. 226–228 °C. ^1^H-NMR (400 MHz, DMSO-*d_6_*) δ 8.68 (s, 1H, Ar-H), 8.37 (s, 1H, Ar-H), 8.15 (s, 1H, Ar-H), 8.04–7.50 (m, 2H, Ar-H), 5.63 (s, 2H, ArCH_2_-), 4.38 (q, *J* = 4.9 Hz, 2H, *N*CH_2_-), 4.22 (q, *J* = 5.2 Hz, 2H, OCH_2_-), 3.43 (s, 3H, *N*CH_3_), 3.19 (s, 3H, *N*CH_3_), 1.35 (t, *J* = 4.9 Hz, 3H, -CH_3_), 1.28 (t, *J* = 5.2 Hz, 3H, -CH_3_). ^13^C-NMR (101 MHz, DMSO-*d_6_*) δ 173.1, 165.0, 155.0, 151.3, 149.3, 148.7, 143.3, 138.9, 134.3, 132.5, 128.9, 125.6, 118.3, 110.9, 106.2, 60.1, 49.4, 48.4, 29.9, 27.9, 15.2, 14.3. HRMS (*m*/*z*, ESI^+^) calcd for C_22_H_24_N_5_O_5_ 438.1777 [M + H]^+^, found 438.1764. HPLC purity: 96% (*t_R_* = 3.2 min).

*(Ethyl 1-butyl-6-((1,3-dimethyl-2,6-dioxo-1,2,3,6-tetrahydro-7H-purin-7-yl)methyl)-4-oxo-1,4-dihydroquinolone-3-carboxylate)*, **3b**. White powder, yield 40%, m.p. 219–221 °C. ^1^H-NMR (400 MHz, CDCl_3_) δ 8.39 (s, 1H, Ar-H), 8.34 (s, 1H, Ar-H), 7.70 (d, *J* = 7.8 Hz, 1H, Ar-H), 7.63 (s, 1H, Ar-H), 7.40 (d, *J* = 7.8 Hz, 1H, Ar-H), 5.56 (s, 2H, ArCH_2_-), 4.32 (t, *J* = 7.2 Hz, 2H, *N*CH_2_-), 4.10 (q, *J* = 5.2 Hz, 2H, -OCH_2_-), 3.51 (s, 3H, *N*CH_3_), 3.32 (s, 3H, *N*CH_3_), 1.88–1.69 (m, 2H, -CH_2_-), 1.41–1.24 (m, 5H, -CH_2_CH_3_), 0.93 (t, *J* = 5.2 Hz, 3H, -CH_3_). ^13^C-NMR (101 MHz, CDCl_3_) δ 173.8, 165.9, 155.3, 151.7, 149.3, 148.7, 141.3, 138.6, 133.3, 132.3, 129.6, 127.2, 116.9, 111.2, 106.9, 61.1, 54.1, 49.7, 30.9, 29.9, 27.9, 19.9, 14.3, 13.2. HRMS (*m/z*, ESI^+^) calcd for C_24_H_28_N_5_O_5_ 466.2090 [M + H]^+^, found 466.2086. HPLC purity: 94% (*t_R_* = 4.4 min).

*(Ethyl 1-benzyl-6-((1,3-dimethyl-2,6-dioxo-1,2,3,6-tetrahydro-7H-purin-7-yl)methyl)-4-oxo-1,4-dihydroquinolone-3-carboxylate)*, **3c.** White powder, yield 70%, m.p. 202–204 °C. ^1^H-NMR (400 MHz, CDCl_3_) δ 8.53 (s, 1H, Ar-H), 8.33 (s, 1H, Ar-H), 7.56–7.47 (m, 2H, Ar-H), 7.34–7.24 (m, 5H, Ar-H), 7.20 (s, 1H, Ar-H), 5.51 (s, 2H, ArCH_2_-), 5.30 (s, 2H, -CH_2_Ar), 4.31 (q, *J* = 5.0 Hz, 2H, -OCH_2_-), 3.50 (s, 3H, *N*CH_3_), 3.30 (s, 3H, *N*CH_3_), 1.33 (t, *J* = 5.0 Hz, 3H, -CH_3_). ^13^C-NMR (101 MHz, CDCl_3_) δ 174.0, 165.7, 155.3, 154.3, 151.7, 150.3, 148.9, 141.3, 139.3, 134.3, 133.3, 132.3, 129.6, 128.6 (2C), 126.9 (2C), 125.9, 117.9, 112.2, 106.9, 61.4, 57.7, 49.7, 29.6, 28.0, 14.3. HRMS (*m*/*z*, ESI^+^) calcd for C_27_H_26_N_5_O_5_ 500.1934 [M + H]^+^, found 500.1936. HPLC purity: 96% (*t_R_* = 4.2 min).

*(Ethyl 1-(4-bromobenzyl)-6-((1,3-dimethyl-2,6-dioxo-1,2,3,6-tetrahydro-7H-purin-7-yl)methyl)-4-oxo-1,4-dihydroquinolone-3-carboxylate)*, **3d**. White off powder, yield 70%, m.p. 237–239 °C. ^1^H-NMR (400 MHz, DMSO-*d_6_*) δ 8.91 (s, 1H, Ar-H), 8.33 (s, 1H, Ar-H), 8.14 (s, 1H, Ar-H), 7.76–7.65 (m, 2H, Ar-H), 7.51 (d, *J* = 9.9 Hz, 2H, Ar-H), 7.21 (d, *J* = 9.9 Hz, 2H, Ar-H), 5.63 (s, 2H, ArCH_2_-), 5.58 (s, 2H, -CH_2_Ar), 4.24 (q, *J* = 5.6 Hz, 2H, -OCH_2_-), 3.41 (s, 3H, NCH_3_), 3.18 (s, 3H, NCH_3_), 1.29 (t, *J* = 5.6 Hz, 3H, -CH_3_). ^13^C-NMR (101 MHz, DMSO-*d_6_*) δ 173.1, 165.4, 155.3, 151.7, 150.6, 148.9, 143.3, 138.9, 135.9, 134.3, 132.4, 132.3, 129.2, 128.6, 125.9, 121.2, 119.2, 111.2, 106.5, 60.4, 55.7, 48.9, 29.9, 28.0, 15.3. HRMS (*m/z*, ESI^+^) calcd for C_27_H_25_BrN_5_O_5_ 578.1039 [M + H]^+^, found 578.1039. HPLC purity: 95% (*t_R_* = 6.4 min).

*(6-((1,3-Dimethyl-2,6-dioxo-1,2,3,6-tetrahydro-7H-purin-7-yl)methyl)-1-ethyl-N-(2-((2-hydroxyethyl)amino)ethyl)-4-oxo-1,4-dihydroquinolone-3-carboxamide)*, **4a**. White powder, yield 35%, m.p. 207–209 °C. ^1^H-NMR (400 MHz, DMSO-*d_6_*) δ 10.06 (t, *J* = 4.8 Hz, 1H, -CONH-), 8.84 (s, 1H, Ar-H), 8.40 (s, 1H, Ar-H), 8.23 (s, 1H, Ar-H), 7.90 (d, *J* = 8.6 Hz, 1H, Ar-H), 7.85 (d, *J* = 8.6 Hz, 1H, Ar-H), 5.65 (s, 2H, ArCH_2_-), 5.01 (s, 1H, OH), 4.49 (q, *J* = 7.7 Hz, 2H, NCH_2_-), 3.67–3.53 (m, 5H, 2 × -CH_2_-, -NH-), 3.42 (s, 3H, NCH_3_), 3.18 (s, 3H, NCH_3_), 2.97 (d, *J* = 9.8 Hz, 2H, -CH_2_-), 2.85 (d, *J* = 9.8 Hz, 2H, -CH_2_-), 1.37 (t, *J* = 7.7 Hz, 3H, -CH_3_). ^13^C-NMR (101 MHz, DMSO-*d_6_*) δ 175.4, 165.0, 154.6, 151.3, 149.3, 148.3, 143.6, 138.9, 134.6, 132.9, 127.9, 125.6, 118.6, 111.9, 106.5, 58.4, 50.7, 49.4, 49.0, 47.7, 37.3, 30.3, 28.3, 15.3. HRMS (*m/z*, ESI^+^) calcd for C_24_H_30_N_7_O_5_ 496.2308 [M + H]^+^, found 496.2303. HPLC purity: 98% (*t_R_* = 1.9 min).

*(6-((1,3-Dimethyl-2,6-dioxo-1,2,3,6-tetrahydro-7H-purin-7-yl)methyl)-1-ethyl-N-(2-(2-hydroxyethoxy)ethyl)-4-oxo-1,4-dihydroquinolone-3-carboxamide)*, **4b**. White off powder, yield 40%, m.p. 211–213 °C. ^1^H-NMR (400 MHz, DMSO-*d_6_*) δ 10.01 (s, 1H, -CONH-), 8.85 (s, 1H, Ar-H), 8.38 (s, 1H, Ar-H), 8.25 (s, 1H, Ar-H), 7.92 (d, *J* = 8.2 Hz, 1H, Ar-H), 7.84 (d, *J* = 8.2 Hz, 1H, Ar-H), 5.65 (s, 2H, ArCH_2_-), 4.61 (s, 1H, OH), 4.49 (q, *J* = 7.9 Hz, 2H, NCH_2_-), 3.67–3.08 (m, 14H, 2 × NCH_3_, 4 × -CH_2_-), 1.36 (t, *J* = 7.9 Hz, 3H, -CH_3_). ^13^C-NMR (101 MHz, DMSO-*d_6_*) δ 175.7, 164.3, 154.9, 151.3, 149.3, 148.3, 143.3, 138.6, 134.3, 133.3, 127.9, 125.6, 118.6, 111.6, 106.5, 72.8, 69.8, 60.7, 49.4, 48.9, 39.4, 29.9, 27.6, 14.9. HRMS (*m/z*, ESI^+^) calcd for C_24_H_29_N_6_O_6_ 479.2149 [M + H]^+^, found 497.2154. HPLC purity: 95% (*t_R_* = 2.7 min).

*(1-Butyl-6-((1,3-dimethyl-2,6-dioxo-1,2,3,6-tetrahydro-7H-purin-7-yl)methyl)-N-(2-(2-hydroxyethoxy)ethyl)-4-oxo-1,4-dihydroquinolone-3-carboxamide)*, **4c**. White powder, yield 30%, m.p. 211–213 °C. ^1^H-NMR (400 MHz, CDCl_3_) δ 10.20 (s, 1H, CONH), 8.71 (s, 1H, Ar-H), 8.36 (s, 1H, Ar-H), 7.75 (d, *J* = 8.0 Hz, 1H, Ar-H), 7.64 (s, 1H, Ar-H), 7.47 (d, *J* = 8.0 Hz, 1H, Ar-H), 5.57 (s, 2H, ArCH_2_-), 4.18 (t, *J* = 7.0 Hz, 2H, NCH_2_-), 3.84–3.70 (m, 4H, 2 × -CH_2_-), 3.70–3.59 (m, 4H, 2 × -CH_2_-), 3.51 (s, 3H, NCH_3_), 3.32 (s, 3H, NCH_3_), 2.69 (s, 1H, OH), 1.88–1.71 (m, 2H, -CH_2_-), 1.43–1.24 (m, 2H, -CH_2_-), 0.92 (t, *J* = 6.9 Hz, 3H, -CH_3_). ^13^C-NMR (101 MHz, CDCl_3_) δ 176.1, 165.4, 155.0, 151.7, 149.3, 148.7, 148.0, 141.3, 140.9, 138.9, 133.3, 132.9, 126.6, 116.9, 107.2, 72.8, 69.7, 62.1, 54.3, 49.7, 38.9, 31.3, 29.6, 27.9, 19.9, 13.6. HRMS (*m/z*, ESI^+^) calcd for C_26_H_33_N_6_O_6_ 525.2462 [M + H]^+^, found 525.2460. HPLC purity: 95% (*t_R_* = 3.4 min).

*(1-Benzyl-6-((1,3-dimethyl-2,6-dioxo-1,2,3,6-tetrahydro-7H-purin-7-yl)methyl)-N-(2-((2-hydroxyethyl)amino)ethyl)-4-oxo-1,4-dihydroquinolone-3-carboxamide)*, **4d**. White powder, yield 55%, m.p. 188–190 °C. ^1^H-NMR (400 MHz, DMSO-*d_6_*) δ 9.98 (t, *J* = 5.0 Hz, 1H, CONH), 9.05 (s, 1H, Ar-H), 8.33 (s, 1H, Ar-H), 8.24 (s, 1H, Ar-H), 7.81–7.67 (m, 2H, Ar-H), 7.42–7.14 (m, 5H, Ar-H), 5.75 (s, 2H, ArCH_2_-), 5.60 (s, 2H, -CH_2_Ar), 4.48 (s, 1H, OH), 3.53–3.33 (m, 8H, NCH_3_, -NH-, 2 × -CH_2_-), 3.17 (s, 3H, NCH_3_), 2.71 (t, *J* = 9.6 Hz, 2H, -CH_2_-), 2.62 (s, *J* = 9.6 Hz, 2H, -CH_2_-). ^13^C-NMR (101 MHz, DMSO-*d_6_*) δ 176.1, 165.0, 155.0, 151.0, 149.3, 148.9, 143.3, 139.3, 136.6, 134.3, 132.6, 129.2, 128.3, 127.9, 127.2, 125.6, 118.9, 112.2, 106.5, 61.0, 56.4, 51.9, 49.4, 48.9, 39.4, 29.9, 28.3. HRMS (*m/z*, ESI^+^) calcd for C_29_H_32_N_7_O_5_ 558.2465 [M + H]^+^, found 558.2465. HPLC purity: 95% (*t_R_* = 2.0 min).

*(1-Benzyl-6-((1,3-dimethyl-2,6-dioxo-1,2,3,6-tetrahydro-7H-purin-7-yl)methyl)-N-(2-(2-hydroxyethoxy)ethyl)-4-oxo-1,4-dihydroquinolone-3-carboxamide)*, **4e**. White powder, yield 60%, m.p. 193–195 °C. ^1^H-NMR (400 MHz, DMSO-*d_6_*) δ 10.00 (s, 1H, CONH), 9.05 (s, 1H, Ar-H), 8.37–8.18 (m, 2H, Ar-H), 7.84–7.62 (m, 2H, Ar-H), 7.40–7.11 (m, 5H, Ar-H), 5.74 (s, 2H, ArCH_2_-), 5.59 (s, 2H, -CH_2_Ar), 4.63 (s, 1H, OH), 3.75–3.27 (m, 11H, NCH_3_, 4 × -CH_2_-), 3.16 (s, 3H, NCH_3_). ^13^C-NMR (101 MHz, DMSO-*d_6_*) δ 176.1, 164.7, 155.0, 151.3, 149.7, 148.7, 142.9, 138.9, 136.6, 134.6, 132.6, 129.6, 128.6, 128.3, 126.9, 125.6, 119.2, 111.9, 106.2, 72.8, 69.8, 60.7, 56.4, 48.9, 39.3, 29.9, 27.9. HRMS (*m/z*, ESI^+^) calcd for C_29_H_31_N_6_O_6_ 559.2305 [M + H]^+^, found 559.2313. HPLC purity: 95% (*t_R_* = 3.2 min).

*(1-Benzyl-6-((1,3-dimethyl-2,6-dioxo-1,2,3,6-tetrahydro-7H-purin-7-yl)methyl)-N-(2-methoxyethyl)-4-oxo-1,4-dihydroquinolone-3-carboxamide)*, **4f**. Brown powder, yield 60%, m.p. 191–193 °C. ^1^H-NMR (400 MHz, CDCl_3_) δ 10.05 (s, 1H, CONH), 8.89 (s, 1H, Ar-H), 8.33 (s, 1H, Ar-H), 7.67–7.51 (m, 2H, Ar-H), 7.37 (s, 1H, Ar-H), 7.31–7.14 (m, 5H, Ar-H), 5.52 (s, 2H, ArCH_2_-), 5.38 (s, 2H, -CH_2_Ar), 3.60 (t, *J* = 8.6 Hz, 2H, -CH_2_-), 3.54 (t, *J* = 8.6 Hz, 2H, -CH_2_-), 3.50 (s, 3H, -OCH_3_), 3.35 (s, 3H, NCH_3_), 3.29 (s, 3H, NCH_3_). ^13^C-NMR (101 MHz, CDCl_3_) δ 176.8, 165.0, 155.7, 151.7, 148.9, 148.3, 140.9, 139.6, 134.3, 133.9, 132.9, 132.6, 129.6, 128.6, 126.6, 126.2, 118.3, 111.0, 106.9, 71.4, 59.1, 57.7, 49.4, 39.7, 29.9, 27.6. HRMS (*m/z*, ESI^+^) calcd for C_28_H_29_N_6_O_5_ 529.2199 [M + H]^+^, found 529.2202. HPLC purity 94% (*t_R_* = 4.2 min).

*(1-(4-Bromobenzyl)-6-((1,3-dimethyl-2,6-dioxo-1,2,3,6-tetrahydro-7H-purin-7-yl)methyl)-N-(2-((2-hydroxyethyl)amino)ethyl)-4-oxo-1,4-dihydroquinolone-3-carboxamide)*, **4g**. White powder, yield 60%, m.p. 222–224 °C. ^1^H-NMR (400 MHz, CDCl_3_) δ 10.18 (s, 1H, CONH), 8.89 (s, 1H, Ar-H), 8.39 (s, 1H, Ar-H), 7.68 (d, *J* = 9.6 Hz, 2H, Ar-H), 7.54–7.20 (m, 5H, Ar-H), 5.59 (s, 2H, ArCH_2_-), 5.40 (s, 2H, -CH_2_Ar), 4.36 (s, 1H, OH), 3.86–3.47 (m, 8H, -NH-, NCH_3_, 2 × -CH_2_-), 3.37 (s, 3H, NCH_3_), 3.08–2.57 (m, 4H, 2 × -CH_2_-), 2.69 (s, 1H, OH). ^13^C-NMR (101 MHz, CDCl_3_) δ 176.4, 165.4, 155.3, 151.7, 149.3, 148.9, 141.6, 138.9, 133.2, 132.6, 128.6, 127.9, 126.9, 126.6, 122.6, 117.9, 112.8, 107.2, 97.9, 60.7, 57.0, 50.7, 49.4, 48.4, 39.4, 30.3, 27.9. HRMS (*m/z*, ESI^+^) calcd for C_29_H_31_BrN_7_O_5_ 636.1570 [M + H]^+^, found 636.1570. HPLC purity 96% (*t_R_* = 2.0 min).

*(1-(4-Bromobenzyl)-6-((1,3-dimethyl-2,6-dioxo-1,2,3,6-tetrahydro-7H-purin-7-yl)methyl)-N-(2-(2-hydroxyethoxy)ethyl)-4-oxo-1,4-dihydroquinolone-3-carboxamide)*, **4h**. White powder, yield 60%, m.p. 217–219 °C, ^1^H-NMR (400 MHz, CDCl_3_) δ 10.15 (s, 1H, CONH), 8.83 (s, 1H, Ar-H), 8.34 (s, 1H, Ar-H), 7.67–7.51 (m, 2H, Ar-H), 7.48–7.34 (m, 2H, Ar-H), 7.31–7.12 (m, 3H, Ar-H), 5.52 (s, 2H, ArCH_2_-), 5.33 (s, 2H, -CH_2_Ar), 3.78–3.70 (m, 4H, 2 × -CH_2_-), 3.70–3.56 (m, 4H, 2 × -CH_2_-), 3.49 (s, 3H, NCH_3_), 3.30 (s, 3H, NCH_3_). ^13^C-NMR (101 MHz, CDCl_3_) δ 176.1, 166.0, 155.0, 151.7, 148.9, 148.7, 141.6, 140.9, 138.9, 132.9, 132.6, 128.3, 127.6, 126.6, 122.9, 122.5, 117.9, 112.6, 107.2, 72.8, 69.7, 61.7, 57.7, 49.4, 39.0, 29.9, 27.9. HRMS (*m/z*, ESI^+^) calcd for C_29_H_30_BrN_6_O_6_ 637.1410 [M + H]^+^, found 637.1395. HPLC purity 96% (*t_R_* = 4.0 min).

*(Ethyl 1-ethyl-4-oxo-6-((4-(trifluoromethyl)benzyl)oxy)-1,4-dihydroquinolone-3-carboxylate)*, **7a**. White powder, yield 50%, m.p. 203–205 °C. ^1^H-NMR (400 MHz, CDCl_3_) δ 8.43 (s, 1H, Ar-H), 7.95 (s, 1H, Ar-H), 7.70–7.45 (m, 4H, Ar-H), 7.48–7.25 (m, 2H, Ar-H), 5.17 (s, 2H, ArCH_2_O-), 4.32 (q, *J* = 6.5 Hz, 2H, *N*CH_2_-), 4.20 (q, *J* = 5.6 Hz, 2H, -OCH_2_-), 1.47 (t, *J* = 6.5 Hz, 3H, -CH_3_), 1.34 (t, *J* = 5.6 Hz, 3H, -CH_3_). ^13^C-NMR (101 MHz, CDCl_3_) δ 173.8, 166.0, 156.4, 147.9, 139.9, 133.2, 130.6, 130.2, 128.3, 127.6, 125.6, 123.2, 122.5, 110.2, 108.8, 69.4, 61.0, 49.4, 14.9, 14.3. HRMS (*m/z*, ESI^+^) calcd for C_22_H_21_F_3_NO_4_ 420.1423 [M + H]^+^, found 420.1419. HPLC purity 95% (*t_R_* = 8.2 min).

*(Methyl 1-benzyl-7-chloro-4-oxo-6-((4-(trifluoromethyl)benzyl)oxy)-1,4-dihydroquinolone-3-carboxylate)*, **7b**. White powder, yield 60%, m.p. 248–250 °C. ^1^H-NMR (400 MHz, DMSO-*d_6_*) δ 8.90 (s, 1H, Ar-H), 7.87 (s, 1H, Ar-H), 7.80 (d, *J* = 7.7 Hz, 2H, Ar-H), 7.68 (d, *J* = 7.7 Hz, 2H, Ar-H), 7.48–7.11 (m, 6H, Ar-H), 5.70 (s, 2H, ArCH_2_O-), 5.44 (s, 2H, CH_2_Ar), 3.77 (s, 3H, -OCH_3_). ^13^C-NMR (101 MHz, DMSO-*d_6_*) δ 172.4, 165.4, 151.3, 150.3, 141.6, 136.6, 134.3, 129.3, 128.9, 128.4, 128.3, 127.9, 126.9, 125.9, 125.9, 120.2, 110.2, 109.9, 109.2, 70.1, 56.1, 51.7. HRMS (*m*/*z*, ESI^+^) calcd for C_26_H_20_ClF_3_NO_4_ 502.1026 [M + H]^+^, found 502.1026. HPLC purity 95% (*t_R_* = 4.7 min).

*(1-Ethyl-N-(2-(2-hydroxyethoxy)ethyl)-4-oxo-6-((4-(trifluoromethyl)benzyl)oxy)-1,4-dihydroquinolone-3-carboxamide)*, **8a**. White powder, yield 30%, m.p. 185–187 °C. ^1^H-NMR (400 MHz, CDCl_3_) δ 10.37 (s, 1H, CONH), 8.72 (s, 1H, Ar-H), 7.96 (s, 1H,Ar-H), 7.73–7.56 (m, 4H, Ar-H), 7.51–7.28 (m, 2H, Ar-H), 5.19 (s, 2H, ArCH_2_O-), 4.28 (q, *J* = 6.6 Hz, 2H, -*N*CH_2_-), 3.92–3.50 (m, 8H, 4 × -CH_2_-), 2.74 (s, 1H, OH), 1.48 (t, *J* = 6.6 Hz, 3H, -CH_3_). ^13^C-NMR (101 MHz, CDCl_3_) δ 176.4, 165.4, 155.9, 146.3, 140.7, 133.9, 130.9, 128.3, 127.9, 125.6, 123.9, 122.6, 118.6, 117.6, 107.9, 73.1, 70.1, 69.8, 62.1, 49.4, 39.4, 14.6. HRMS (*m*/*z*, ESI^+^) calcd for C_24_H_26_F_3_N_2_O_5_ 479.1794 [M + H]^+^, found 479.1788. HPLC purity 95% (*t_R_* = 9.6 min).

*(1-Butyl-N-(2-(2-hydroxyethoxy)ethyl)-4-oxo-6-((4-(trifluoromethyl)benzyl)oxy)-1,4-dihydroquinolone-3-carboxamide)*, **8b**. White powder, yield 36%, m.p. 190–192 °C. ^1^H-NMR (400 MHz, CDCl_3_) δ 10.37 (s, 1H, CONH), 8.73 (s, 1H, Ar-H), 7.92 (s, 1H, Ar-H), 7.73–7.53 (m, 4H, Ar-H), 7.47–7.34 (m, 2H, Ar-H), 5.19 (s, 2H, ArCH_2_O-), 4.21 (t, *J* = 7.2 Hz, 2H, *N*CH_2_-), 3.91–3.42 (m, 8H, 2 × -CH_2_-), 2.46 (s, 1H, OH), 1.48–1.28 (m, 2H, -CH_2_-), 1.28–1.09 (m, 2H, -CH_2_-), 0.91 (t, *J* = 6.8 Hz, 3H, -CH_3_). ^13^C-NMR (101 MHz, CDCl_3_) δ 175.4, 156.7, 146.9, 140.6, 134.3, 130.9, 129.3, 128.6, 125.9, 125.6, 123.9, 122.5, 117.9, 111.2, 107.5, 73.1, 70.1, 69.8, 61.7, 38.7, 31.2, 29.6, 19.9, 12.9. HRMS (*m*/*z*, ESI^+^) calcd for C_26_H_30_F_3_N_2_O_5_ 507.2107 [M + H]^+^, found 507.2105. HPLC purity 94% (*t_R_* = 4.7 min).

*(7-Chloro-1-ethyl-N-(2-((2-hydroxyethyl)amino)ethyl)-4-oxo-6-((4-(trifluoromethyl)benzyl)oxy)-1,4-dihydroquinolone-3-carboxamide)*, **8c**. White powder, yield 40%, m.p. 209–211 °C. ^1^H-NMR (400 MHz, DMSO-*d_6_*) δ 10.00 (s, 1H, CONH), 8.81 (s, 1H, Ar-H), 8.16 (s, 1H, Ar-H), 7.95 (s, 1H, Ar-H), 7.90–7.65 (m, 4H, Ar-H), 5.50 (s, 2H, ArCH_2_O-), 4.51 (q, *J* = 7.6 Hz, 2H, NCH_2_-), 3.78 (s, 1H, OH), 3.64–3.41 (m, 5H, -NH-, 2 × -CH_2_-), 2.85–2.60 (m, 4H, 2 × -CH_2_-), 1.36 (t, *J* = 7.6 Hz, 3H, -CH_3_). ^13^C-NMR (101 MHz, DMSO-*d_6_*) δ 174.8, 164.7, 151.3, 148.0, 144.3, 141.6, 134.3, 129.3, 128.2, 127.6, 125.9, 123.2, 119.9, 111.12, 108.8, 70.1, 60.7, 52.0, 48.9, 48.7, 39.4, 15.5. HRMS (*m/z*, ESI^+^) calcd for C_24_H_26_ClF_3_N_3_O_4_ 512.1564 [M + H]^+^, found 512.1563. HPLC purity 95% (*t_R_* = 1.9 min).

*(7-Chloro-1-ethyl-N-(2-(2-hydroxyethoxy)ethyl)-4-oxo-6-((4-(trifluoromethyl)benzyl)oxy)-1,4-dihydroquinolone-3-carboxamide)*, **8d**. White powder, yield 30%, m.p. 226–228 °C. ^1^H-NMR (400 MHz, CDCl_3_) δ 10.22 (s, 1H, CONH), 8.68 (s, 1H, Ar-H), 7.92 (s, 1H, Ar-H), 7.72–7.41 (m, 5H, Ar-H), 5.26 (s, 2H, ArCH_2_O), 4.22 (q, *J* = 6.9 Hz, 2H, *N*CH_2_-), 3.83–3.72 (m, 4H, 2 × -CH_2_-), 3.66–3.50 (m, 4H, 2 × -CH_2_-), 3.19 (s, 1H, OH), 1.45 (t, *J* = 6.9 Hz, 3H, -CH_3_). ^13^C-NMR (101 MHz, CDCl_3_) δ 175.4, 164.9, 151.7, 146.7, 139.6, 135.3, 133.6, 130.6, 128.6, 127.9, 125.6, 122.5, 117.9, 112.2, 108.8, 72.1, 69.8, 69.4, 61.7, 49.7, 39.4, 14.6. HRMS (*m*/*z*, ESI^+^) calcd for C_24_H_25_ClF_3_N_2_O_5_ 513.1404 [M + H]^+^, found 513.1404. HPLC purity: 96% (*t_R_* = 4.7 min).

*(1-Butyl-7-chloro-N-(2-(2-hydroxyethoxy)ethyl)-4-oxo-6-((4-(trifluoromethyl)benzyl)oxy)-1,4-dihydroquinolone-3-carboxamide)*, **8e**. White powder, yield 30%, m.p. 218–220 °C. ^1^H-NMR (400 MHz, DMSO-*d_6_*) δ 10.03 (s, 1H, CONH), 8.78 (s, 1H, Ar-H), 8.15 (s, 1H, Ar-H), 7.95 (s, 1H, Ar-H), 7.90–7.62 (m, 4H, Ar-H), 5.48 (s, 2H, ArCH_2_O), 4.62 (s, 1H, OH), 4.47 (t, *J* = 7.2 Hz, 2H, *N*CH_2_-), 3.75–3.45 (m, 8H, 4 × -CH_2_-), 1.93–1.56 (m, 2H, -CH_2_-), 1.43–1.20 (m, 2H, -CH_2_-), 0.89 (t, *J* = 6.9 Hz, 3H, -CH_3_). ^13^C-NMR (101 MHz, DMSO-*d_6_*) δ 174.7, 164.7, 151.3, 148.3, 141.9, 134.3, 132.3, 131.9, 129.3, 128.2, 127.6, 125.9, 111.6, 110.5, 108.6, 72.8, 69.8, 61.0, 53.4, 49.7, 39.4, 31.3, 19.6, 14.3. HRMS (*m*/*z*, ESI^+^) calcd for C_26_H_29_ClF_3_N_2_O_5_ 541.1717 [M + H]^+^, found 541.1713. HPLC purity 96% pure (*t_R_* = 6.4 min).

*(1-Butyl-7-chloro-N-(2-methoxyethyl)-4-oxo-6-((4-(trifluoromethyl)benzyl)oxy)-1,4-dihydroquinolone-3-carboxamide)*, **8f**. White powder, yield 40%, m.p. 224–226 °C. ^1^H-NMR (400 MHz, CDCl_3_) δ 10.07 (s, 1H, CONH), 8.65 (s, 1H, Ar-H), 7.96 (s, 1H, Ar-H), 7.70–7.40 (m, 5H, Ar-H), 5.25 (s, 2H, ArCH_2_O-), 4.14 (t, *J* = 6.8 Hz, 2H, *N*CH_2_-), 3.67–3.44 (m, 4H, 2 × -CH_2_-), 3.35 (s, 3H, -OCH_3_), 1.96–1.73 (m, 2H, -CH_2_-), 1.56–1.15 (m, 2H, -CH_2_-), 0.92 (t, *J* = 7.0 Hz, 3H, -CH_3_). ^13^C-NMR (101 MHz, CDCl_3_) δ 175.4, 165.0, 151.3, 147.3, 139.9, 133.6, 130.9, 129.5, 127.9, 127.2, 125.6, 122.3, 118.3, 111.9, 108.8, 71.7, 70.1, 59.1, 54.3, 39.4, 31.0, 19.9, 13.6. HRMS (*m*/*z*, ESI^+^) calcd for C_25_H_27_ClF_3_N_2_O_4_ 511.1611 [M + H]^+^, found 511.1615. HPLC purity 97% (*t_R_* = 5.2 min).

*(1-Benzyl-7-chloro-N-(2-((2-hydroxyethyl)amino)ethyl)-4-oxo-6-((4-(trifluoromethyl)benzyl)oxy)-1,4-dihydroquinolone-3-carboxamide)*, **8g**. White powder, yield 30%, m.p. 225–227 °C. ^1^H-NMR (400 MHz, DMSO-*d_6_*) δ 10.01 (s, 1H, CONH), 9.00 (s, 1H, Ar-H), 7.98 (s, 1H, Ar-H), 7.95 (s, 1H, Ar-H), 7.79 (d, *J* = 7.6 Hz, 2H, Ar-H), 7.73 (d, *J* = 7.6 Hz, 2H, Ar-H), 7.48–7.09 (m, Hz, 5H, Ar-H), 5.79 (s, 2H, ArCH_2_O-), 5.46 (s, 2H, -CH_2_Ar), 4.49 (s, 1H, OH), 3.67–3.45 (m, 5H, -NH-, 2 × -CH_2_-), 2.73 (t, *J* = 8.3 Hz, 2H, -CH_2_-), 2.63 (t, *J* = 8.3 Hz, 2H, -CH_2_-). ^13^C-NMR (101 MHz, DMSO-*d_6_*) δ 175.0, 164.3, 151.3, 148.7, 141.9, 136.3, 134.3, 129.5, 129.1, 128.6, 128.5, 128.2, 127.9, 126.9, 125.9, 123.2, 120.2, 111.6, 108.8, 70.1, 60.7, 56.4, 52.0, 49.4, 39.7. HRMS (*m/z*, ESI^+^) calcd for C_29_H_28_ClF_3_N_3_O_4_ 574.1720 [M + H]^+^, found 574.1724. HPLC purity 96% (*t_R_* = 4.3 min).

*(1-Benzyl-7-chloro-N-(2-(2-hydroxyethoxy)ethyl)-4-oxo-6-((4-(trifluoromethyl)benzyl)oxy)-1,4-dihydroquinolone-3-carboxamide)*, **8h**. White powder, yield 40%, m.p. 229–231 °C. ^1^H-NMR (400 MHz, CDCl_3_) δ 10.22 (s, 1H, CONH), 8.80 (s, 1H, Ar-H), 7.93 (s, 1H, Ar-H), 7.60 (d, *J* = 8.2 Hz, 2H, Ar-H), 7.56 (d, *J* = 8.2 Hz, 2H, Ar-H), 7.40–7.19 (m, 6H, Ar-H), 5.35 (s, 2H, ArCH_2_O), 5.22 (s, 2H, -CH_2_Ar), 4.32 (s, 1H, OH), 3.80–3.71 (m, 4H, 2 × -CH_2_-), 3.66–3.55 (m, 4H, 2 × -CH_2_-). ^13^C-NMR (101 MHz, CDCl_3_) δ 175.4, 165.0, 151.7, 147.6, 139.9, 133.9, 133.7, 130.0, 129.5, 128.9, 127.9, 127.8, 127.3, 126.1, 126.1, 125.6, 118.9, 111.9, 108.5, 72.8, 70.1, 69.4, 62.1, 58.4, 38.9. HRMS (*m*/*z*, ESI^+^) calcd for C_29_H_27_ClF_3_N_2_O_5_ 575.1561 [M + H]^+^, found 575.1563. HPLC purity 96% (*t_R_* = 2.0 min).

*(Ethyl 1-ethyl-6-nitro-4-oxo-1,4-dihydroquinolone-3-carboxylate)*, **11**. Yellow powder, yield 70%, m.p. 189–191 °C. ^1^H-NMR (400 MHz, CDCl_3_) δ 9.23 (s, 1H, Ar-H), 8.49 (d, *J* = 8.2 Hz, 1H, Ar-H), 8.39 (dd, *J* = 8.2, 2.3 Hz, 1H, Ar-H), 7.54 (s, *J* = 2.3 Hz, 1H, Ar-H), 4.35 (q, *J* = 6.9 Hz, 2H, NCH_2_-), 4.25 (q, *J* = 6.7 Hz, 2H, OCH_2_-), 1.53 (t, *J* = 6.7 Hz, 3H, -CH_3_), 1.35 (t, *J* = 6.9 Hz, 3H, -CH_3_). ^13^C-NMR (101 MHz, CDCl_3_) δ 173.1, 164.7, 149.9, 144.3, 142.3, 128.9, 126.6, 124.2, 116.9, 112.8, 61.0, 48.9, 14.9, 14.6. HRMS (*m*/*z*, ESI^+^) calcd for C_14_H_15_N_2_O_5_ 291.0981 [M + H]^+^, found 291.0972. HPLC purity 94% (*t_R_* = 4.9 min).

*(Ethyl 6-amino-1-benzyl-4-oxo-1,4-dihydroquinolone-3-carboxylate)*, **12**. Red powder, yield 65%, m.p. 187–189 °C. ^1^H-NMR (400 MHz, DMSO-*d_6_*) δ 8.71 (s, 1H, Ar-H), 7.45–7.28 (m, 5H, Ar-H), 7.28–7.14 (m, 3H, Ar-H), 5.59 (s, 2H, -CH_2_Ar), 5.53 (s, 2H, -NH_2_), 4.22 (q, *J* = 6.2 Hz, 2H, OCH_2_-), 1.26 (t, *J* = 6.2 Hz, 3H, -CH_3_). ^13^C-NMR (101 MHz, DMSO-*d_6_*) δ 173.4, 165.7, 147.6, 146.9, 136.9, 131.3, 130.2, 129.3, 128.3, 126.9, 120.6, 119.2, 108.5, 107.5, 59.8, 55.7, 14.9. HRMS (*m*/*z*, ESI^+^) calcd for C_19_H_19_N_2_O_3_ 323.1396 [M + H]^+^, found 323.1393. HPLC purity 95% (*t_R_* = 3.6 min).

*(Ethyl -1-benzyl-6-(((5-nitrofuran-2-yl)methylene)amino)-4-oxo-1,4-dihydroquinolone-3-carboxylate)*, **13**. Brown powder, yield 72%, m.p. 193–195 °C. ^1^H-NMR (400 MHz, DMSO-*d_6_*) δ 8.93 (s, 1H, Ar-H), 8.74 (s, 1H,N=CH-), 8.14 (d, *J* = 7.8 Hz, 1H, Ar-H), 7.84 (d, *J* = 7.8 Hz, 1H, Ar-H), 7.73 (s, 1H, Ar-H), 7.56–7.17 (m, 7H, Ar-H), 5.73 (s, 2H, -CH_2_Ar), 4.25 (q, *J* = 6.6 Hz, 2H, -OCH_2_-), 1.31 (t, *J* = 6.6 Hz, 3H, -CH_3_). ^13^C-NMR (101 MHz, DMSO-*d_6_*) δ 173.1, 164.7, 153.4, 150.6, 149.7, 146.9, 138.9, 136.6, 131.9, 129.5, 128.6, 127.6, 126.9, 120.2, 119.5, 118.6, 117.9, 114.9, 111.2, 60.4, 56.4, 14.6. HRMS (*m*/*z*, ESI^+^) calcd for C_24_H_20_N_3_O_6_ 446.1352 [M + H]^+^, found 446.1353. HPLC purity 95% (*t_R_* = 3.6 min).

*(6-Amino-1-benzyl-N-(2-(2-hydroxyethoxy)ethyl)-4-oxo-1,4-dihydroquinolone-3-carboxamide)*, **14**. White powder, yield 40%, m.p. 169–171 °C. ^1^H-NMR (400 MHz, DMSO-*d_6_*) δ 10.28 (s, 1H, CONH), 8.85 (s, 1H, Ar-H), 7.50–7.40 (m, 1H, Ar-H), 7.42–7.25 (m, 2H, Ar-H), 7.25–7.09 (m, 5H, Ar-H), 5.72 (s, 2H, -CH_2_Ar), 5.60 (s, 2H, -NH_2_), 4.35 (s, 1H, OH), 3.70–3.53 (m, 4H, 2 × -CH_2_-), 3.50–3.45 (m, 4H, 2 × -CH_2_-). ^13^C-NMR (101 MHz, DMSO-*d_6_*) δ 175.7, 165.7, 147.6, 146.6, 137.3, 131.3, 129.6, 129.3, 128.2, 126.9, 121.9, 119.2, 109.9, 107.2, 73.1, 70.1, 61.0, 56.4, 39.7. HRMS (*m*/*z*, ESI^+^) calcd for C_21_H_24_N_3_O_4_ 382.1767 [M + H]^+^, found 382.1764. HPLC purity 96% (*t_R_* = 2.8 min).

*(Methyl (E)-Methyl-6-(3-(2,6-dichlorophenyl)acryloyl)-1-ethyl-4-oxo-1,4-dihydroquinolone-3-carboxylate)*, **16**. A 100 mL round bottom flask was charged with 20 mL of MeOH, **15** (500 mg, 1.7 mmol), NaOH (200 mg, 5 mmol, 3eq.) and 2,6-dichlorobenzaldehyde (365 mg, 2 mmol, 1.2 eq.). The resultant reaction mixture was allowed to stir at room temperature for 12 h. The product precipitated out during the course of reaction, and was filtered, washed twice with 10 mL portions of distilled water and dried to obtain target compound **16**. White off powder, yield 40%, m.p. 247–249 °C. ^1^H-NMR (400 MHz, CDCl_3_) δ 9.14 (s, 1H, Ar-H), 8.55 (s, 1H, Ar-H), 8.39 (d, *J* = 11.0 Hz, 1H, =CH), 8.12–7.79 (m, 3H, Ar-H), 7.59 (d, *J* = 11.0 Hz, 1H, =CH), 7.40 (d, *J* = 8.2 Hz, 1H, Ar-H), 7.28 (m, 2H, overlap with CDCl_3_), 4.34 (q, *J* = 6.5 Hz, 2H, NCH_2_-), 3.95 (s, 3H, -OCH_3_), 1.61 (t, *J* = 6.5 Hz, 3H, -CH_3_). ^13^C-NMR (101 MHz, CDCl_3_) δ 188.8, 174.0, 166.4, 149.3, 141.6, 138.3, 135.3, 134.3, 132.6, 130.1, 129.6, 129.3, 128.9, 128.7, 116.9, 112.9, 111.9, 52.4, 49.4, 14.6. HRMS (*m*/*z*, ESI^+^) calcd for C_22_H_18_NO_4_ 430.0600 [M + H]^+^, found 430.0600. HPLC purity 97% (*t_R_* = 8.4 min).

### 3.3. In Vitro Antimycobacterial Assay

The minimum inhibitory concentration (MIC) was determined using the standard broth micro-dilution method. Briefly, a 10 mL culture of *Mycobacterium tuberculosis* pMSp12::GFP (REF) [61,62,63] was grown to an optical density (OD600) of 0.6–0.7 in Middlebrook 7H9 supplemented with 0.03% casitone, 0.4% glucose, and 0.05% tyloxpol [64]. Cultures were diluted 1:500 prior to inoculation into the MIC assay. The compounds to be tested were reconstituted to a concentration of 10 mM in DMSO. Two-fold serial dilutions of the test compound were prepared across a 96-well micro-titre plate, after which 50 μL of the diluted *M. tuberculosis* cultures were added to each well in the serial dilution. The plate layout was a modification of the method previously described [65]. Assay controls used were a minimum growth control (Rifampicin at 2× MIC), and a maximum growth control (5% DMSO). The micro-titre plates were sealed in a secondary container and incubated at 37 °C with 5% CO_2_ and humidification. Relative fluorescence (excitation 485 nM; emission 520 nM) was measured using a plate reader (FLUOstar OPTIMA, BMG LABTECH, Ortenberg, Germany) at day 14. The raw fluorescence data were archived and analysed using the CDD Vault from Collaborative Drug Discovery, in which data were normalised to the minimum and maximum inhibition controls to generate a dose response curve (% inhibition) using the Levenberg-Marquardt damped least squares method, from which the MIC_90_ was calculated (www.collaborativedrug.com; Burlingame, CA, USA). The lowest concentration of drug that inhibited growth of more than 90% of the bacterial population was considered the MIC_90_.

### 3.4. In Vitro Antiplasmodial Assay

The 3D7 strain of *Plasmodium falciparum* was routinely cultured in medium consisting of RPMI1640 containing 25 mM Hepes (Lonza, Basel, Switzerland), supplemented with 0.5% (*w*/*v*) Albumax II (Thermo Fisher Scientific, Waltham, MA, USA), 22 mM glucose, 0.65 mM hypoxanthine, 0.05 mg/mL gentamicin and 2–4% (*v*/*v*) human erythrocytes. Cultures were maintained at 37 °C under an atmosphere of 5% CO_2_, 5% O_2_, 90% N_2_. To assess antiplasmodial activity, three-fold serial dilutions of test compounds in culture medium were added to parasite cultures (adjusted to 2% parasitaemia, 1% haematocrit) in 96-well plates and incubated for 48 h. Duplicate wells per compound concentration were used. Parasite lactate dehydrogenase (pLDH) enzyme activity in the individual wells was determined in a similar manner as previously described [66].

### 3.5. In Vitro Cytotoxicity Assay

HEK293 cells (Cellonex, Johannesburg, South Africa) seeded in 96-well plates were incubated with 20 µM test compounds for 24 h and cell viability assessed using a resazurin fluorescence assay as previously described [67].

### 3.6. In Vitro Anti-Trypanosomal Assay

*Trypanosoma brucei brucei* 427 trypomastigotes were cultured in iscove’s modified Dulbecco’s medium (IMDM; Lonza, Basel, Switzerland) supplemented with 10% fetal calf serum, HMI-9 supplement [68], hypoxanthine and penicillin/streptomycin at 37 °C in a 5% CO_2_ incubator. Serial dilutions of test compounds were incubated with the parasites in 96-well plates for 24 h and residual parasite viability in the wells determined as previously reported [60].

## 4. Conclusions

Herein, several quinolone derivatives of varied lipophilicity were conceptualised and easily accessed through cheap synthetic transformation in moderate to good yields. The resulting compounds demonstrated MIC_90_ values in the range of 0.24–31 µM in a whole cell assay against the pMSp12::GFP strain of *Mtb*. Anti-*Mtb* activity in some instances correlated with increased lipophilicity and in some instances structural features other than increased lipophilicity could also be observed to influence anti-*Mtb* activity. For example, compounds **8c** and **8g** bearing an amino alcohol demonstrated potent activity of 1.9 µM and 0.9 µM, respectively. On the other hand, these compounds also demonstrated potent to moderate activity against the chloroquine susceptible strain (3D7) of *P. falciparum* (IC_50_: 0.4–13.6 µM). Anti-malarial activity was noted to increase with increasing lipophilicity of benzenoid and/or N1 substituents. Compounds **4h** and **8g**, demonstrated IC_50_ values of 0.68 µM and 0.46 µM, respectively against 3D7. Against *T. b. brucei*, these compounds demonstrated moderate to weak activity (IC_50_: 5–26 µM). More importantly, several compounds demonstrated activities against the etiological agents of TB, malaria and sleeping sickness without any cytotoxicity on Human Embryonic Kidney cells (HEK293). The compounds were generally not active against Gram-negative and Gram-positive bacteria.

## Figures and Tables

**Table 1:** ClogP, In vitro anti-*Mtb*, anti-plasmodial, anti-trypanosomal and cytotoxic activities.

Compound	^a^ MIC_90_ (µM)	IC_50_ (µM)			^b^*C*logP
		*Pf* 3D7	*T. b. brucei*	HEK293	
**3a**	>125	>20	>20	>32	1.2
**3b**	>125	>20	>20	>32	2.2
**3c**	>125	>20	14.5	>32	2.5
**3d**	15.6	13.6	21.6	>32	3.3
**4a**	>125	>20	>20	>32	0.9
**4b**	>125	>20	>20	>32	0.7
**4c**	>125	>20	>20	>32	0.3
**4d**	7.9	>20	>20	>32	0.4
**4e**	>125	>20	26.8	>32	0.6
**4f**	>125	>20	>20	>32	1.3
**4g**	7.9	4.1	>20	>32	1.2
**4h**	>125	0.68	>20	>32	1.3
**7a**	>125	2.3	18.5	>32	4.0
**7b**	>125	>20	5.4	>32	5.9
**8a**	7.8	6.8	14.1	>32	2.0
**8b**	>125	2.4	>20	>32	3.1
**8c**	1.9	1.3	1.5	>32	2.4
**8d**	>125	>20	>20	>32	2.6
**8e**	15.6	13.6	4.9	>32	3.7
**8f**	>125	>20	>20	>32	4.3
**8g**	0.9	0.46	0.18	>32	3.7
**8h**	16	>20	>20	>32	3.9
**11**	31.3	>20	>20	>32	1.6
**12**	>125	10	>20	>32	1.9
**13**	8.0	6.5	5.2	>32	2.3
**14**	>125	>20	>20	>32	0.03
**16**	0.9	11.7	1.5	>32	3.8
**RIF**	0.001	-	-	-	1.0
**CQ**	-	0.021	-	-	-
**PE**	-	-	0.017	-	-
**TMF**	-	-	-	9	-

^a^ 14-day Alamar Blue readout against *Mtb* H37Rv in GAST/Fe medium. ^b^ Calculated using ACD Chemsketch freeware version 12.0. RIF = rifampicin, CQ = chloroquine, PE = pentamidine, TMF = tamoxifen.

## Data Availability

The data presented in this study are available in the article and supplementary material file provided.

## Supplementary Materials

The following are available online, Synthetic procedures, ^1^H and ^13^C NMR spectra, Mass spectra and dose response curves of compounds.

## Sample Availability

Samples of the compounds are available from the authors upon reasonable request.
