# Supplementary material for: Easy-To-Access Quinolone Derivatives Exhibiting Antibacterial and Anti-Parasitic Activities
Source: Molecules. 2021 Feb 20;26(4):1141. doi: 10.3390/molecules26041141 (PMC7931078; doi:10.3390/molecules26041141)
Supplement: Supplementary file 1 [file molecules-26-01141-s001.pdf]

Easy-to-access quinolone derivatives exhibiting antibacteria and anti-parasitic activities

Richard M. Beteck[1]*, Audrey Jordaan[2], Ronnett Seldon[3], Dustin Laming[4], Heinrich C. Hoppe[4,5], Digby F. Warner[2,6] and Setshaba D. Khanye[1,4]*

[1] Centre of Excellence for Pharmaceutical Sciences, North-West University, Potchefstroom 2520, South Africa.

[2] SAMRC/NHLS/UCT Molecular Mycobacteriology Research Unit, Department of Pathology and Institute of Infectious Disease and Molecular Medicine, Faculty of Health Sciences, University of Cape Town 7925, South Africa

[3] SAMRC Drug Discovery and Development Research Unit, University of Cape Town, Cape Town 7700, South Africa

[4] Centre for Chemico- and Biomedicinal Research, Rhodes University, Makhanda 6140, South Africa

[5] Department of Biochemistry and Microbiology, Faculty of Science, Rhodes University, Makhanda 6140, South Africa

[6] Wellcome Centre for Infectious Diseases Research in Africa, University of Cape Town, Cape Town 7925, South Africa

[7] Division of Pharmaceutical Chemistry, Faculty of Pharmacy, Rhodes University, Makhanda 6140, South Africa

*****Correspondence: richmb1@yahoo.com, 25159194@nwu.ac.za (RMB); s.khanye@ru.ac.za (SDK); Tel.: +27-46-603-8397

4.1 Synthesis of compounds

4.1.1. Nitro reduction using reduce Fe powder. A 1 L round bottom flask was charged with 10 g of aryl nitro, 60 mL of ethanol, followed by 3 molar equivalence of reduced iron powder, and 3 molar equivalence of NH_4Cl. The resulting mixture was refluxed while stirring until reaction completion as indicated by TLC analysis (12 hours). The resulting mixture was concentrated *in vacuo*, dry loaded on to a silica gel column chromatography, and the compound eluted using DCM/MeOH (10:1) as the mobile phase. Collected fractions were combined and the solvents removed *in vacuo* to afford substituted anilines in 70-80 % yield.

4.1.2. Condensation of substituted anilines with diethyl ethoxyethylenemalonate. To a 100 ml flask containing 5g of substituted aniline, was added 30 ml of acetonitrile followed by diethyl ethoxymethylenemalonate (1 eq.). The resulting mixture was stirred under reflux for 10-12 h, after which the mixture was evaporated to dryness to give a crude malonate ester product. This was used in the subsequent step without further purification.

4.1.3. Cyclisation. 2g of malonate ester was added in one portion to 14 ml of pre-heated Dowtherm A and the resultant mixture heated at 245-250 °C for five minutes while stirring. Upon adding petroleum ether to the cooled mixture, a white solid precipitated out. The solid was filtered and washed twice with 20 ml of petroleum ether to afford 1.5 g of 4-oxo-3-carboxy quinolones.

4.1.4. N-alkylation. To a 200 ml round bottom flask containing 30 ml of $CHCl_3$/THF (2:1) mixture was added 1g of 4-oxo-3-carboxy quinolones, K_2CO_3 (5eq.), alkyl/aryl halide (1.2 eq.) and the mixture refluxed for 12 hours. Upon reaction completion as indicated by TLC, the mixture was filtered off K_2CO_3, and the filtrate evaporated to dryness to obtain a crude *N*-alkylated 4-oxo-3-carboxy quinolones, which was purified through silica gel column

chromatography using CH_2Cl_2/MeOH (10:1) as the mobile phase. *N*-alkylated 4-oxo-3-carboxy quinolones were obtained in 50-70 % yield following this procedure.

4.1.5. Ester aminolysis. A mixture of *N*-alkylated 4-oxo-3-carboxy quinolones (1 g, 1 eq.), DBU (1.2 eq.), an appropriate amine (5 eq.), and chloroform (15 mL) in a 100 mL round bottom flask was stirred under reflux for 24h. Upon reaction completion as indicated by TLC, the mixture was evaporated to dryness and resultant crude subjected to silica gel column chromatography eluting with CH_2Cl_2/MeOH (10:1). Fractions containing the desired product were combined, evaporated to dryness and recrystallised from ethanol to afford *N*-alkylated 4-oxo-1,4-dihydroquinolones-3-carboxamides in 30-60 % yield.

4.1.6. Imine formation. A 100mL round bottom flask was charged with 20ml of 95 % ethanol, 400 mg (1.2 mmol) of **12**, few drops of glacial acetic acid and 263mg (1.8 mmol, 1.5 eq.) of 5-nitrofurfural. The mixture was stirred under reflux for 12 hours. The products precipitated out during the course of reaction, and were filtered, washed twice with 10 ml portions of ethanol and dried to obtain 400 mg of target compound **13** in 72 % yield.

Compound 3a

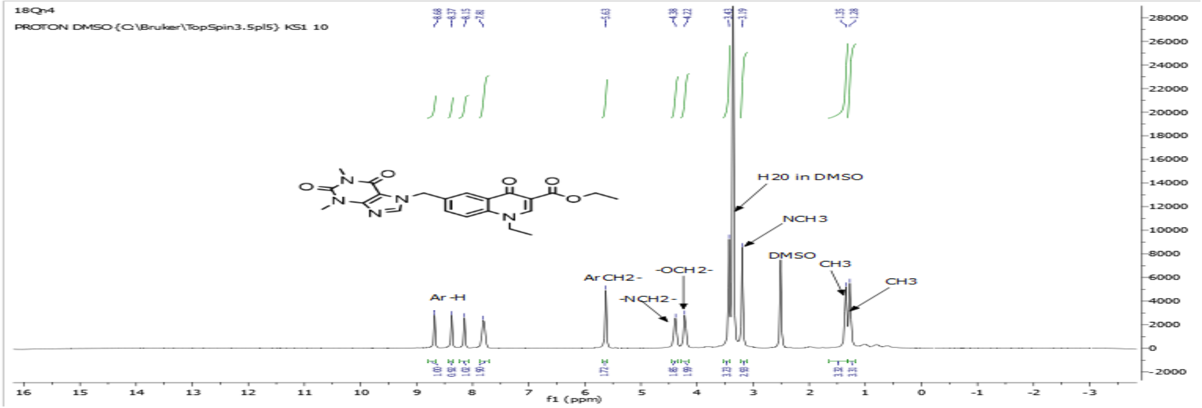

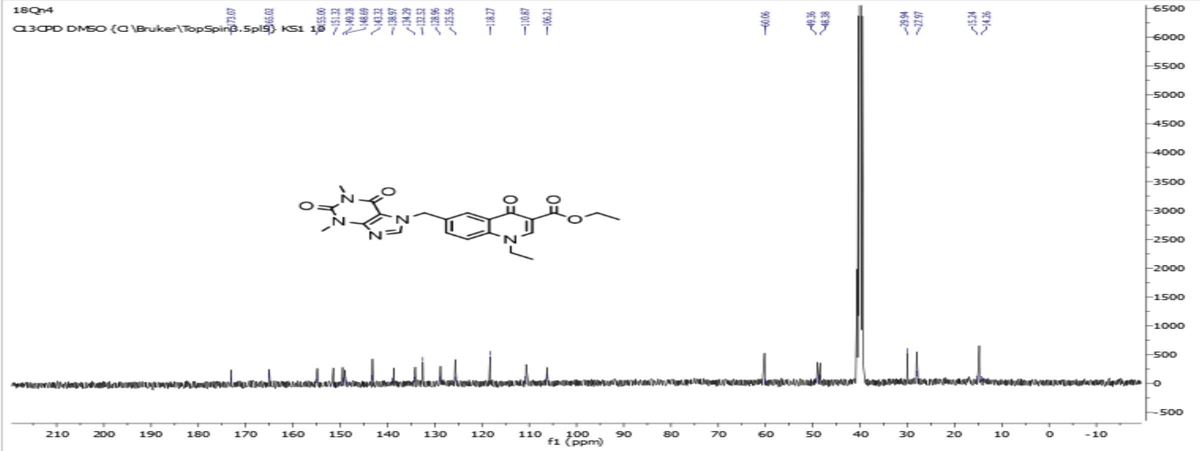

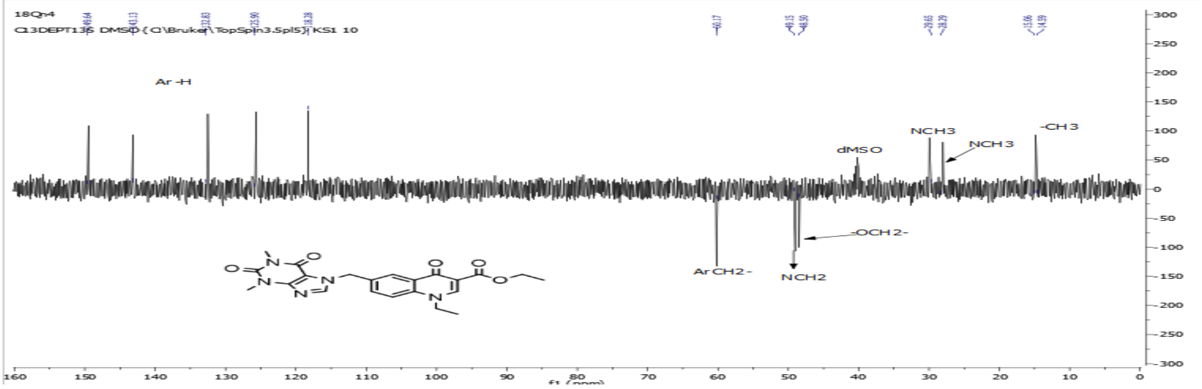

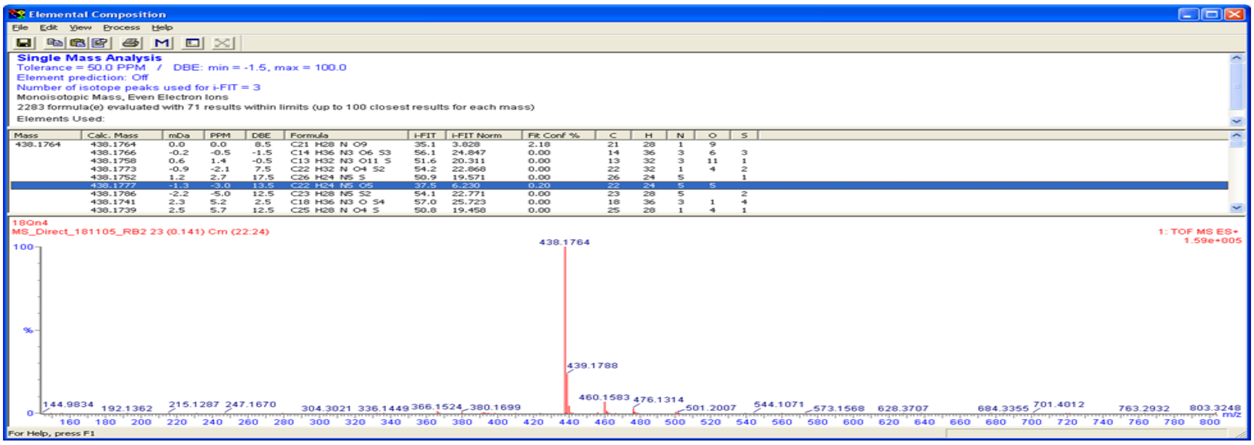
Elemental Composition

Single Mass Analysis
Tolerance = 50.0 PPM / DBE: min = -1.5, max = 100.0
Element prediction: Off
Number of isotope peaks used for i-FIT = 3
Monoisotopic Mass, Even Electron Ions
2283 formula(e) evaluated with 71 results within limits (up to 100 closest results for each mass)
Elements Used:

Mass	Calc. Mass	mDa	PPM	DBE	Formula	i-FIT	i-FIT Norm	Fit Conf %	C	H	N	O	S
438.1764	438.1764	0.0	0.0	8.5	C21 H28 N O9	35.1	3.828	2.18	21	28	1	9	
	438.1766	-0.2	-0.5	-1.5	C14 H36 N3 O6 S3	56.1	24.847	0.00	14	36	3	6	3
	438.1758	0.6	1.4	-0.5	C13 H32 N3 O11 S	51.6	20.311	0.00	13	32	3	11	1
	438.1773	-0.9	-2.1	7.5	C22 H32 N O4 S2	54.2	22.868	0.00	22	32	1	4	2
	438.1752	1.2	2.7	17.5	C26 H24 N5 S	50.9	19.571	0.00	26	24	5		1
	438.1777	-1.3	-3.0	13.5	C22 H24 N5 O5	37.5	6.230	0.20	22	24	5	5	
	438.1786	-2.2	-5.0	12.5	C23 H28 N5 S2	54.1	22.771	0.00	23	28	5		2
	438.1741	2.3	5.2	2.5	C18 H36 N3 O S4	57.0	25.723	0.00	18	36	3	1	4
	438.1739	2.5	5.7	12.5	C25 H28 N O4 S	50.8	19.458	0.00	25	28	1	4	1

Dose-response against Mtb

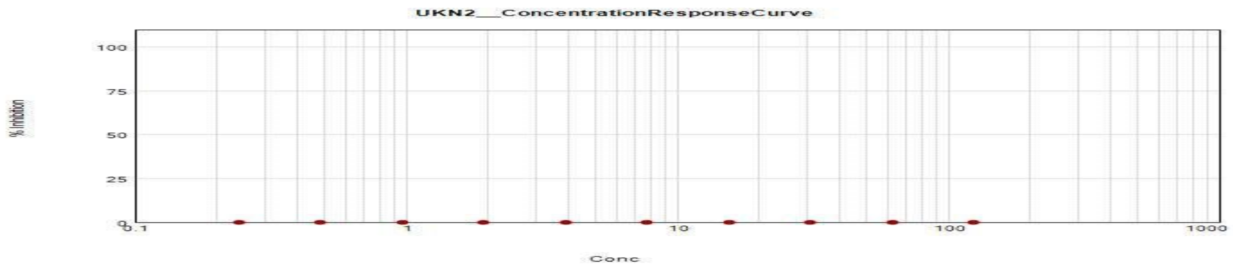

Curve Fit Results

Curve Fit : 4-Parameter $y = D + \frac{A - D}{1 + \left(\frac{x}{C}\right)^B}$

	Parameter	Estimated Value	Std. Error	Confidence Interval
Unknown2	A	No fit		
R^2 =	B	No fit		
EC50 =	C	No fit		
	D	No fit		

Compound 3b

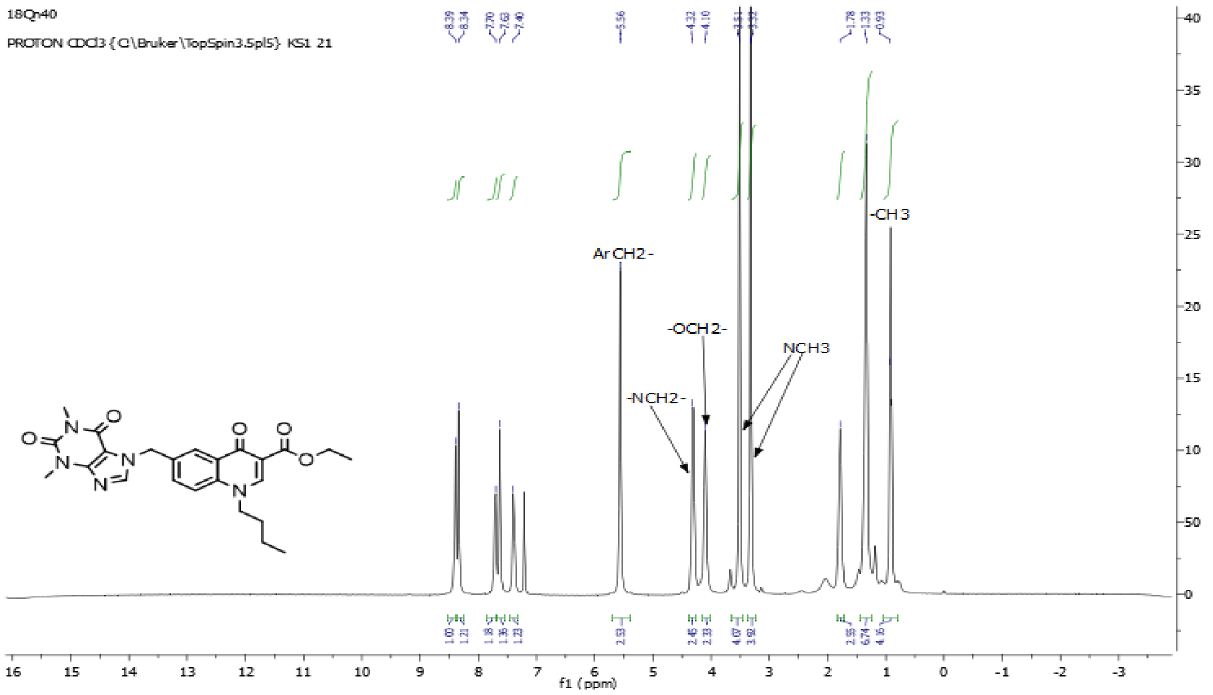

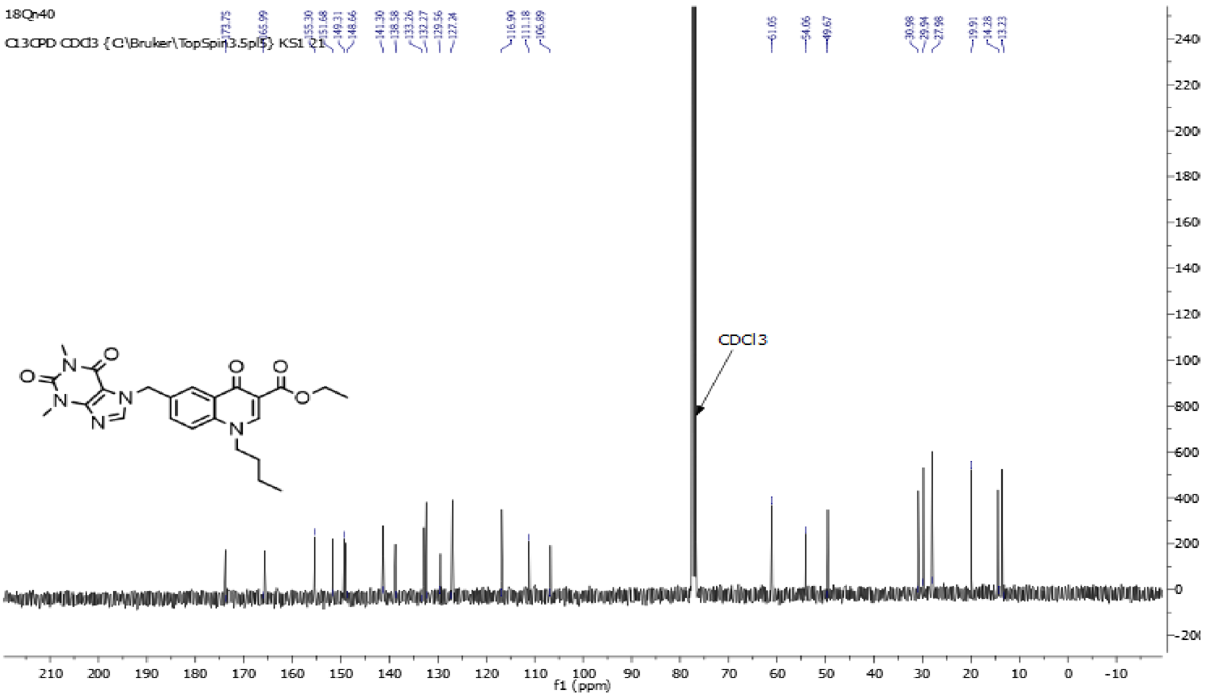
18Qn40
C13CPD CDCl3 {C:\Bruker\TopSpin3.5pl5} KS1 21
173.75
165.99
155.30
151.68
149.31
148.66
141.30
138.58
133.26
132.27
129.56
127.24
116.90
111.18
106.89
61.05
54.06
49.67
30.98
29.94
27.98
19.91
14.28
13.23
CDCl3
f1 (ppm)

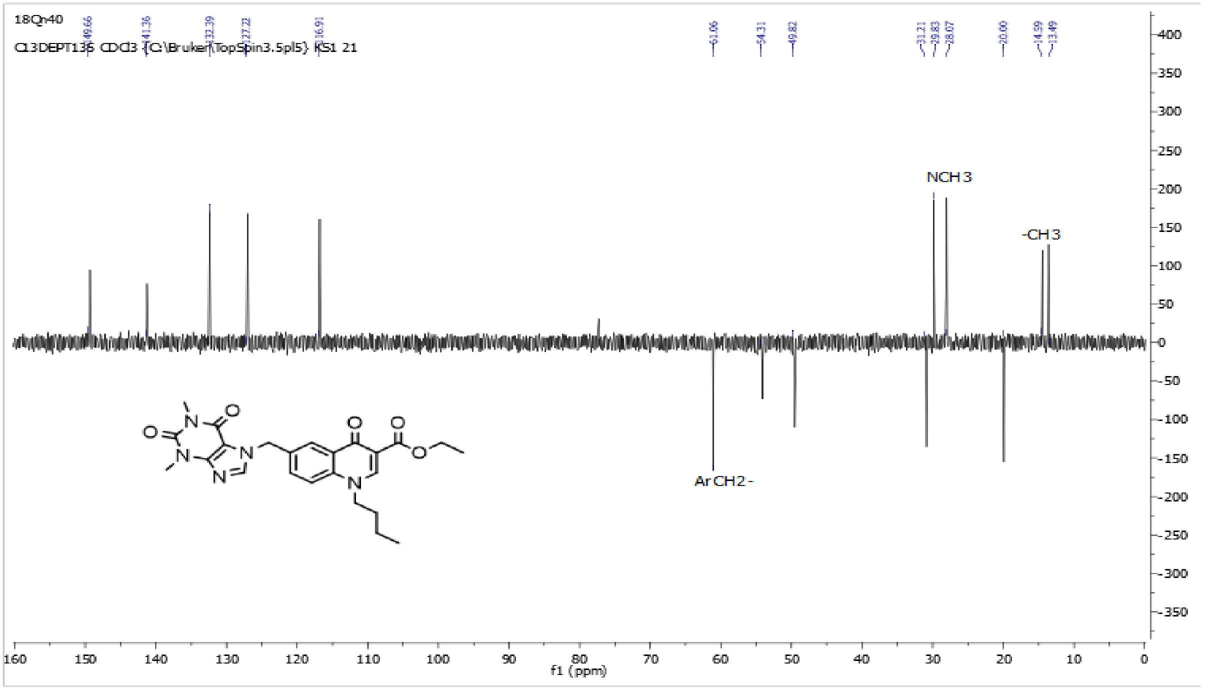
18Qn40
C13DEPT135 CDCl3 {C:\Bruker\TopSpin3.5pl5} KS1 21
149.66
141.36
132.39
127.22
116.91
61.06
54.31
49.82
31.21
29.83
28.07
20.00
14.59
13.49
NCH3
-CH3
ArCH2-
f1 (ppm)

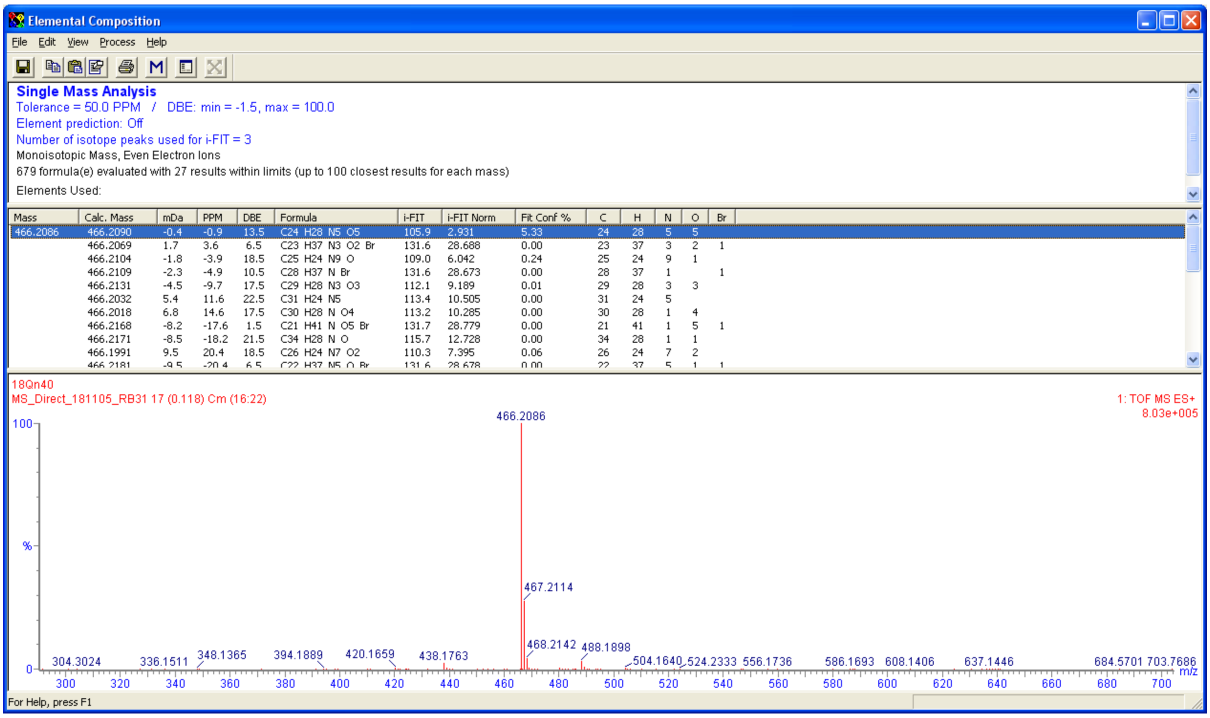

Dose-response against Mtb

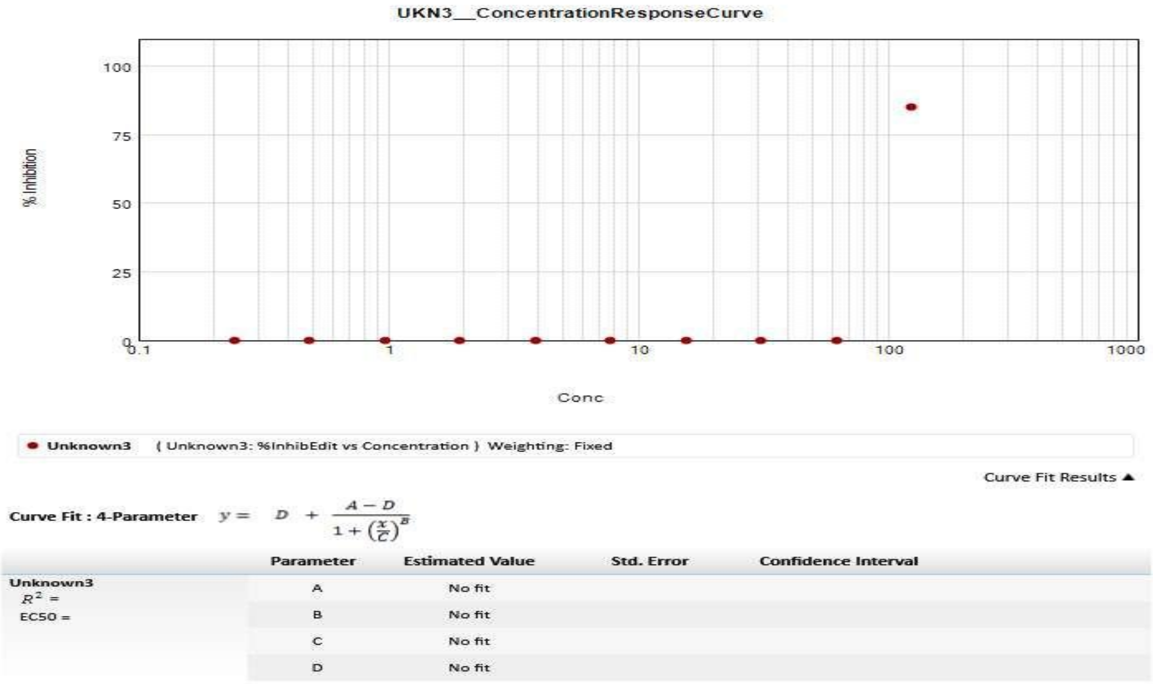

Compound 3c

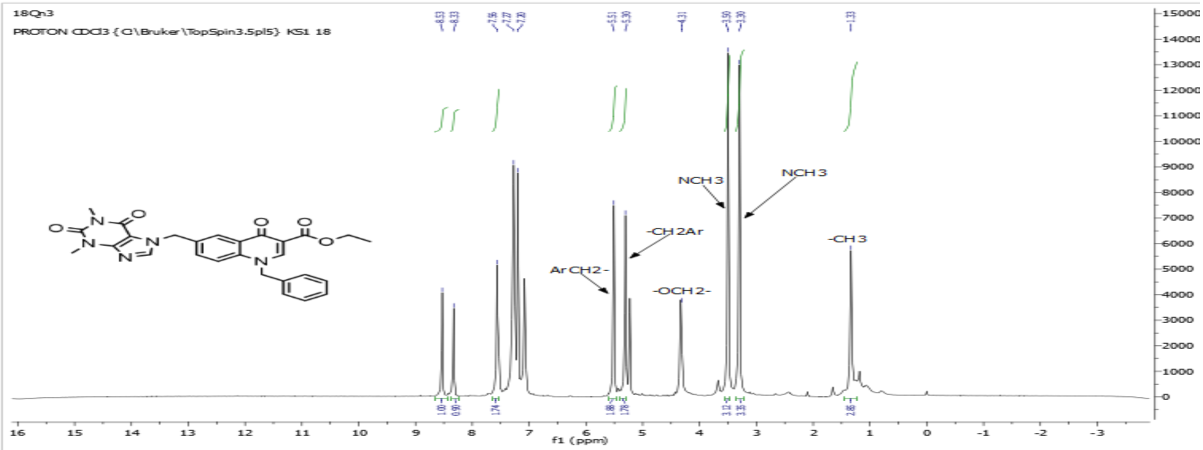
18Qn3
PROTON CDCl3 {C:\Bruker\TopSpin3.5pl5} KS1 18
NCH3
NCH3
-CH2Ar
-CH3
ArCH2-
-OCH2-
f1 (ppm)

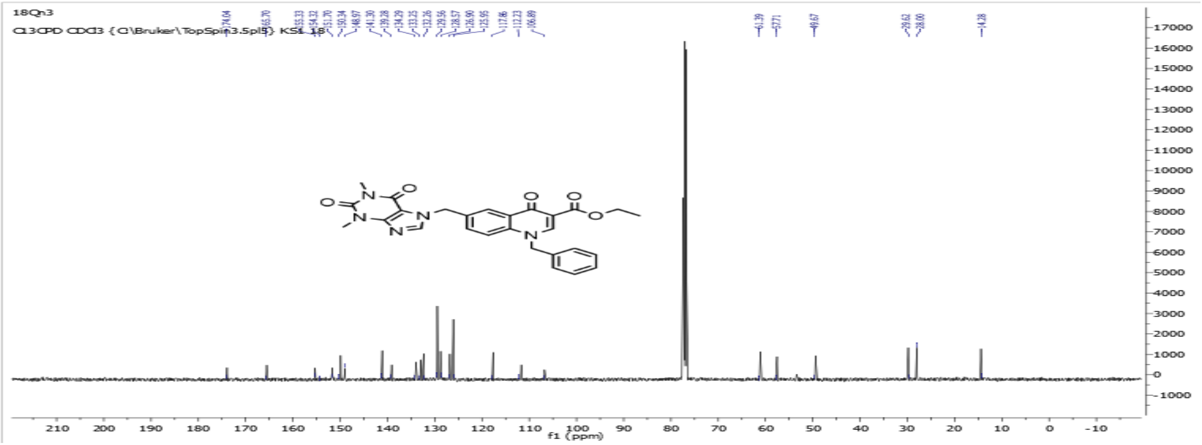
18Qn3
C13CPD CDCl3 {C:\Bruker\TopSpin3.5pl5} KS1 18
f1 (ppm)

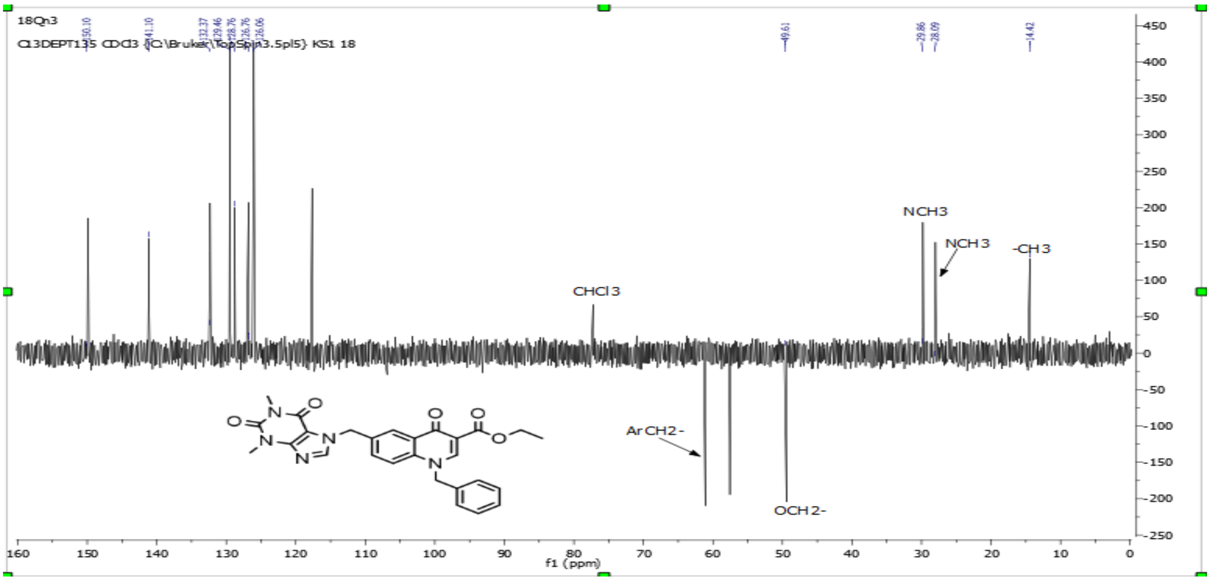
18Qn3
C13DEPT135 CDCl3 {C:\Bruker\TopSpin3.5pl5} KS1 18
NCH3
NCH3
-CH3
CHCl3
ArCH2-
OCH2-
f1 (ppm)

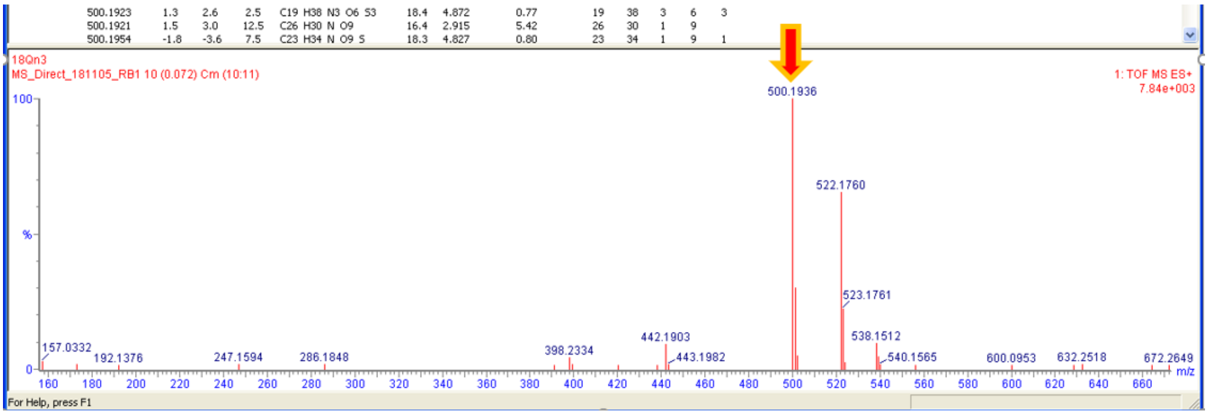

Dose-response against Mtb

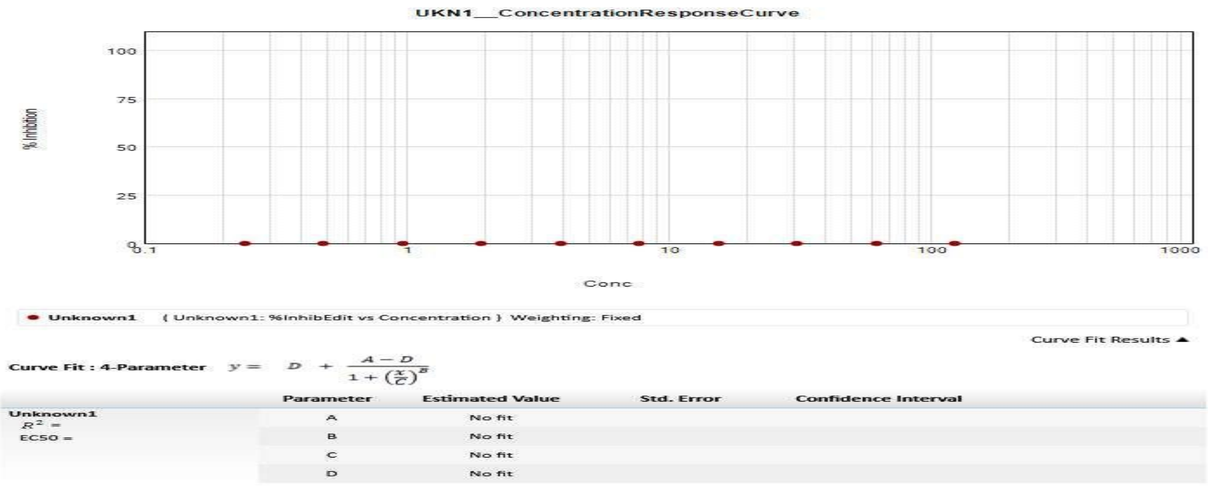

Compound 3d

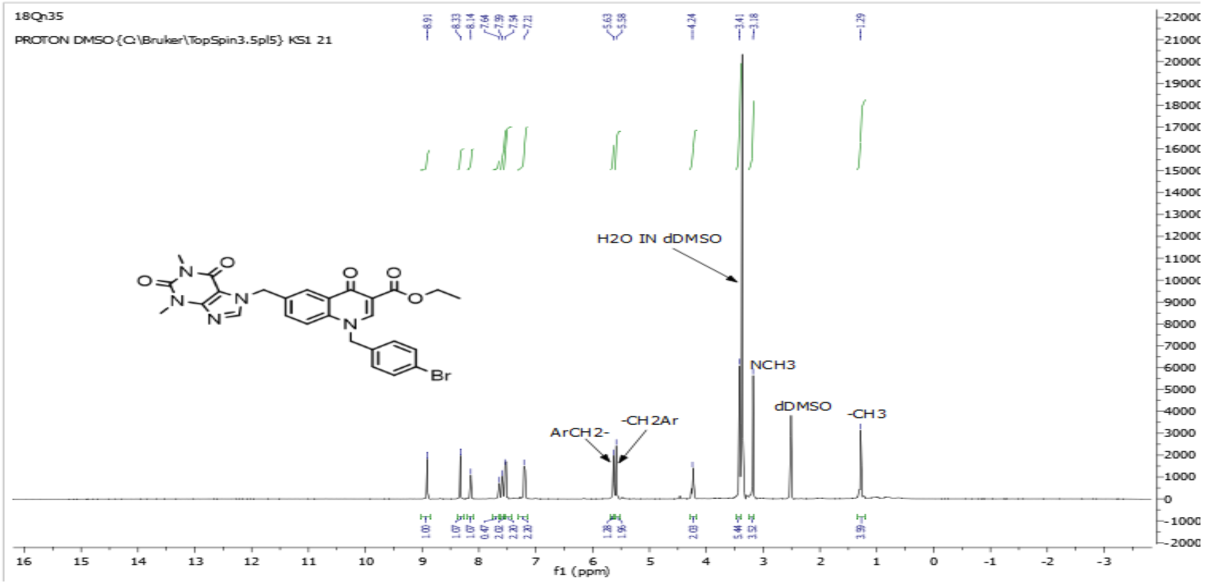

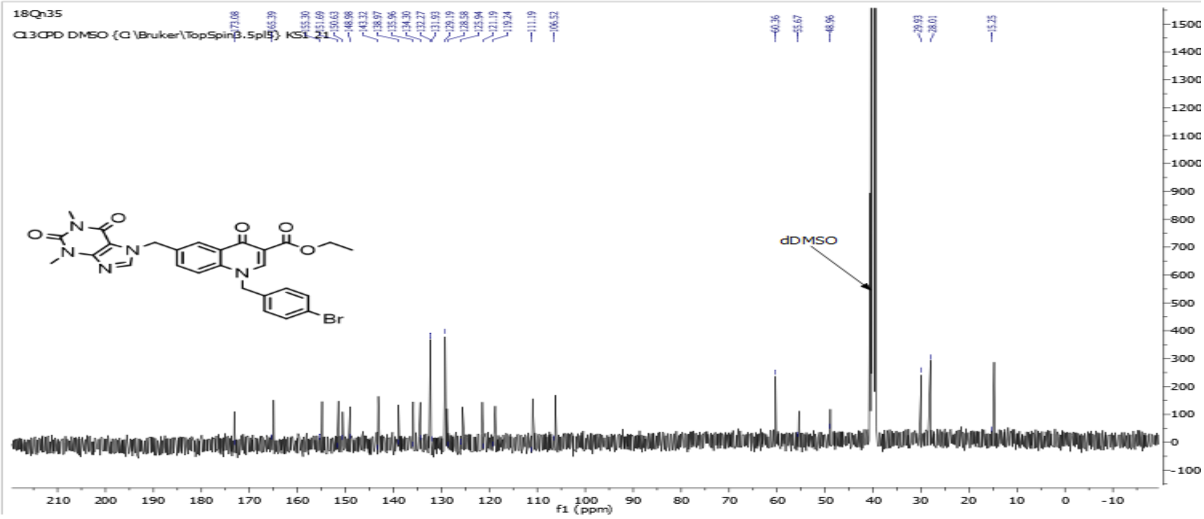

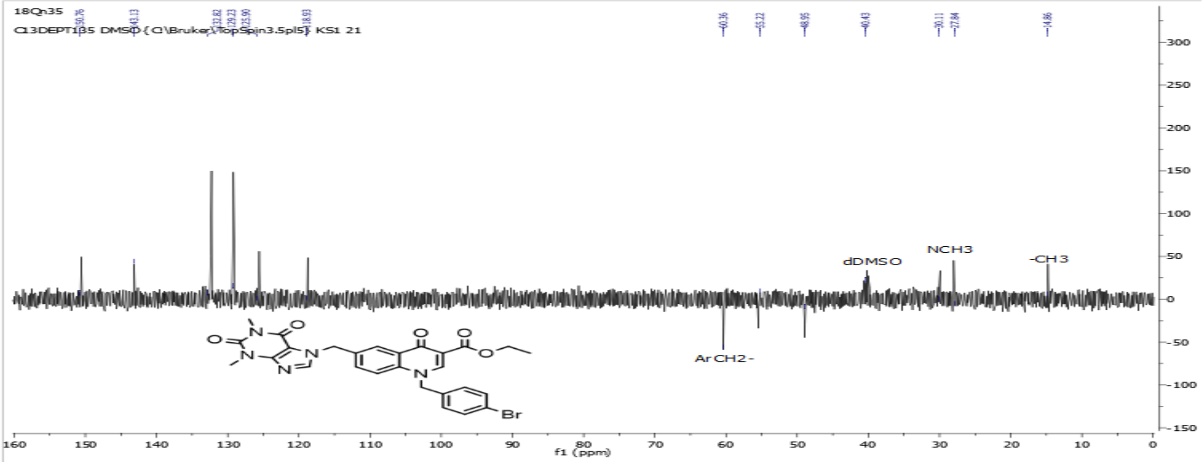

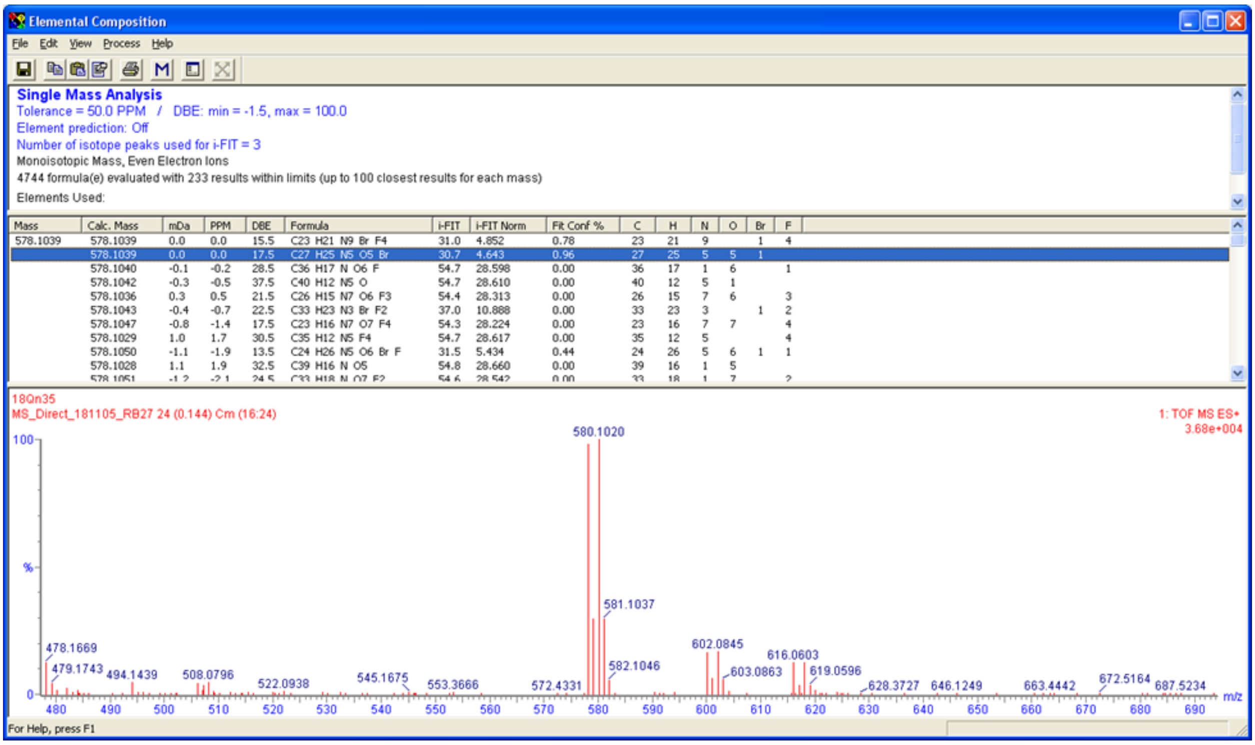

Elemental Composition

File Edit View Process Help

Single Mass Analysis
Tolerance = 50.0 PPM / DBE: min = -1.5, max = 100.0
Element prediction: Off
Number of isotope peaks used for i-FIT = 3
Monoisotopic Mass, Even Electron Ions
4744 formula(e) evaluated with 233 results within limits (up to 100 closest results for each mass)
Elements Used:

Mass	Calc. Mass	mDa	PPM	DBE	Formula	i-FIT	i-FIT Norm	Fit Conf %	C	H	N	O	Br	F
578.1039	578.1039	0.0	0.0	15.5	C23 H21 N9 Br F4	31.0	4.852	0.78	23	21	9		1	4
	578.1039	0.0	0.0	17.5	C27 H25 N5 O5 Br	30.7	4.643	0.96	27	25	5	5	1	
	578.1040	-0.1	-0.2	28.5	C36 H17 N O6 F	54.7	28.598	0.00	36	17	1	6		1
	578.1042	-0.3	-0.5	37.5	C40 H12 N5 O	54.7	28.610	0.00	40	12	5	1		
	578.1036	0.3	0.5	21.5	C26 H15 N7 O6 F3	54.4	28.313	0.00	26	15	7	6		3
	578.1043	-0.4	-0.7	22.5	C33 H23 N3 Br F2	37.0	10.888	0.00	33	23	3		1	2
	578.1047	-0.8	-1.4	17.5	C23 H16 N7 O7 F4	54.3	28.224	0.00	23	16	7	7		4
	578.1029	1.0	1.7	30.5	C35 H12 N5 F4	54.7	28.617	0.00	35	12	5			4
	578.1050	-1.1	-1.9	13.5	C24 H26 N5 O6 Br F	31.5	5.434	0.44	24	26	5	6	1	1
	578.1028	1.1	1.9	32.5	C39 H16 N O5	54.8	28.660	0.00	39	16	1	5		
	578.1051	-1.2	-2.1	24.5	C33 H18 N O7 F2	54.6	28.542	0.00	33	18	1	7		2

Dose-response against Mtb

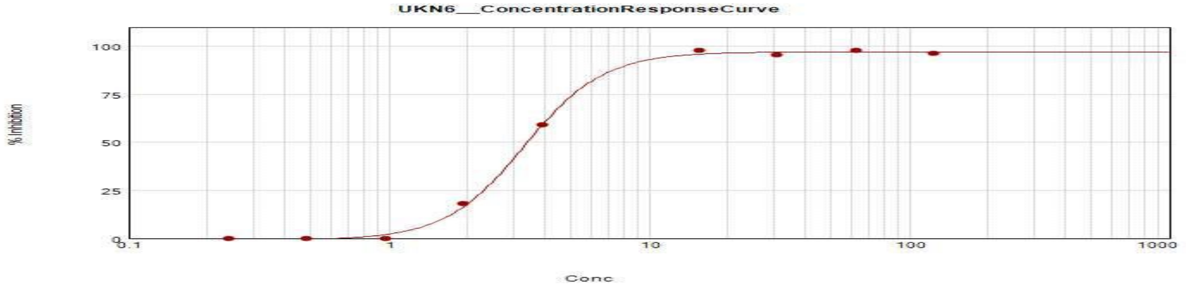

● Unknown6 (Unknown6: %InhibEdit vs Concentration) Weighting: Fixed

Curve Fit Results ▲

Curve Fit : 4-Parameter $y = D + \frac{A - D}{1 + \left(\frac{x}{C}\right)^B}$

	Parameter	Estimated Value	Std. Error	Confidence Interval
Unknown6 $R^2 = 0.999$ EC50 = 3.313	A	-0.896	1.099	[-3.720, 1.928]
	B	2.852	0.206	[2.322, 3.382]
	C	3.313	0.082	[3.104, 3.523]
	D	97.11	0.846	[94.93, 99.29]

Compound 4a

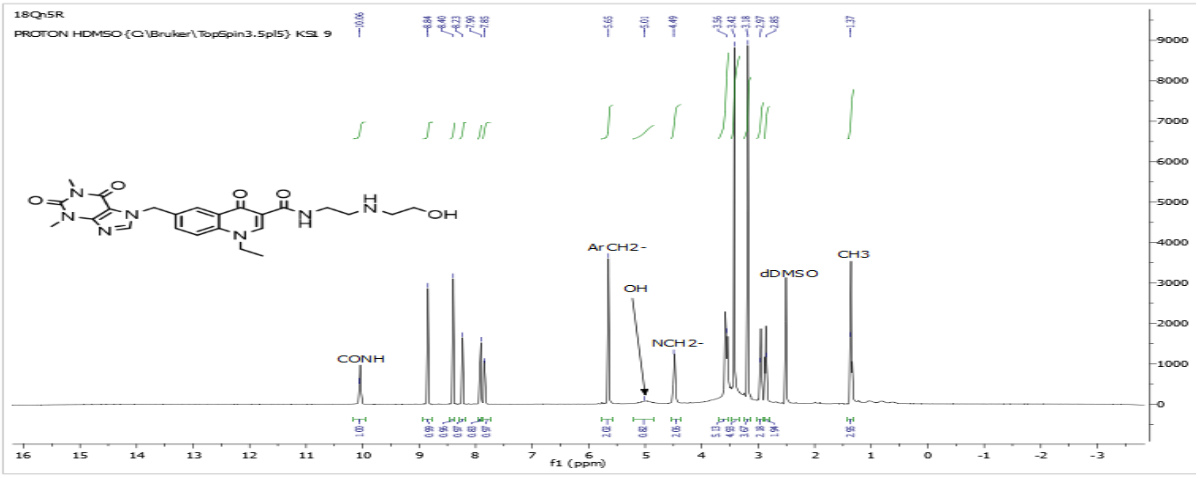

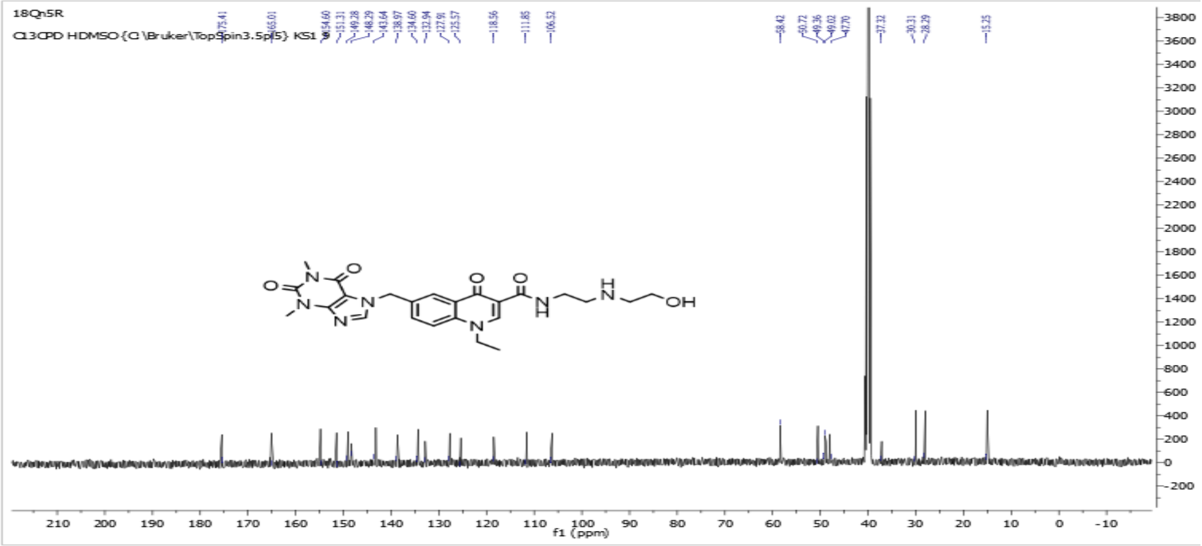

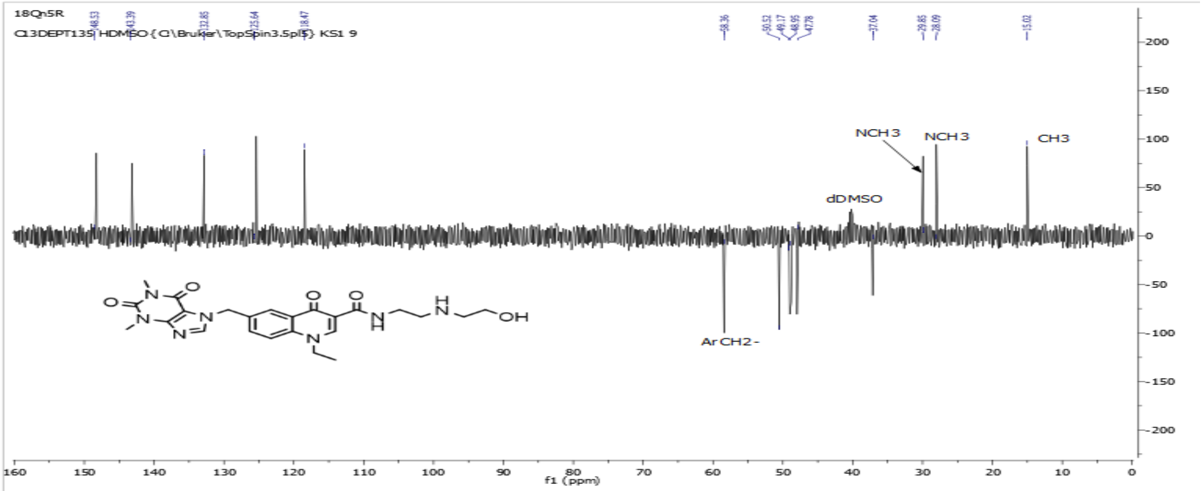

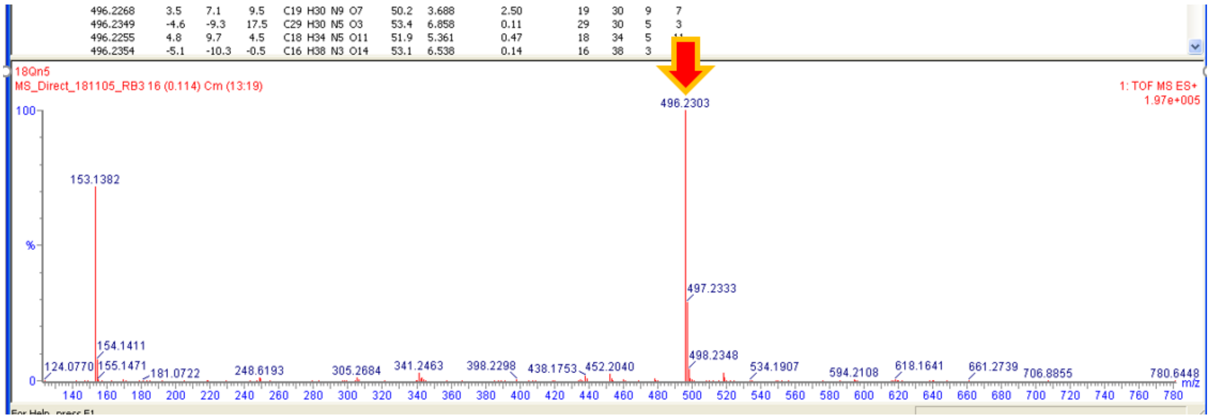

Dose-response against Mtb

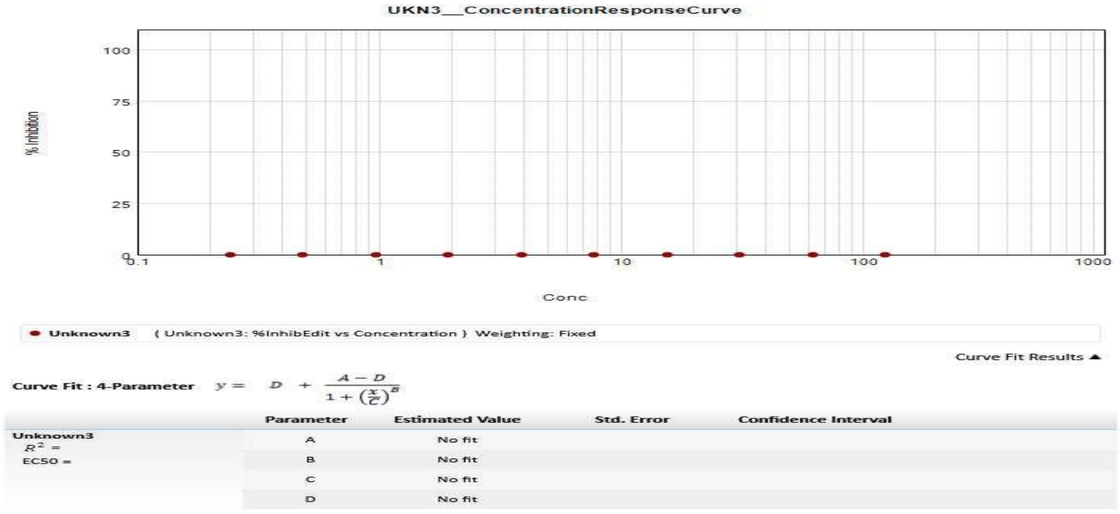

Compound 4b

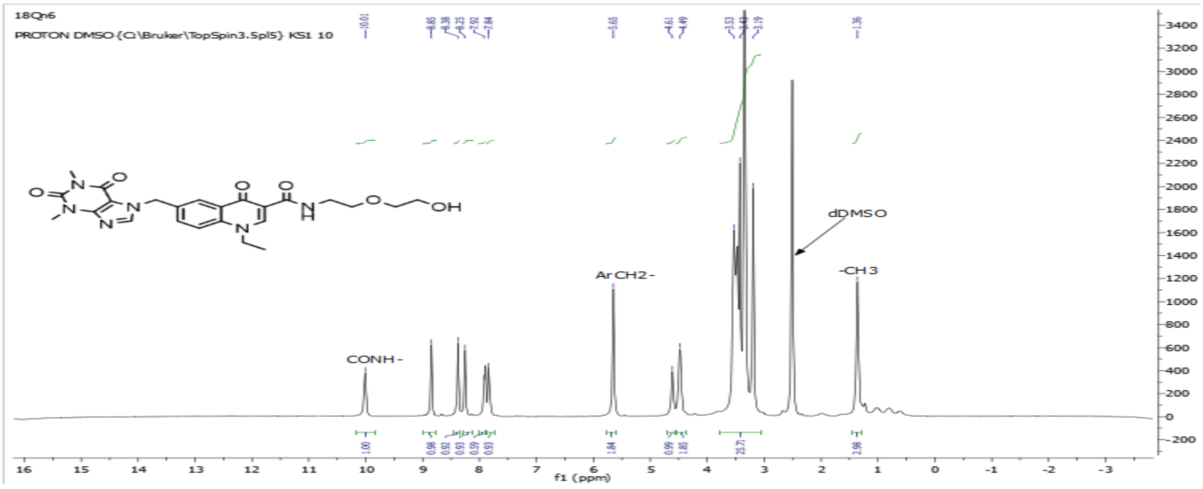
18Qn6
PROTON DMSO {C:\Bruker\TopSpin3.5pl5} KS1 10
CONH-
ArCH2-
dDMSO
-CH3
f1 (ppm)

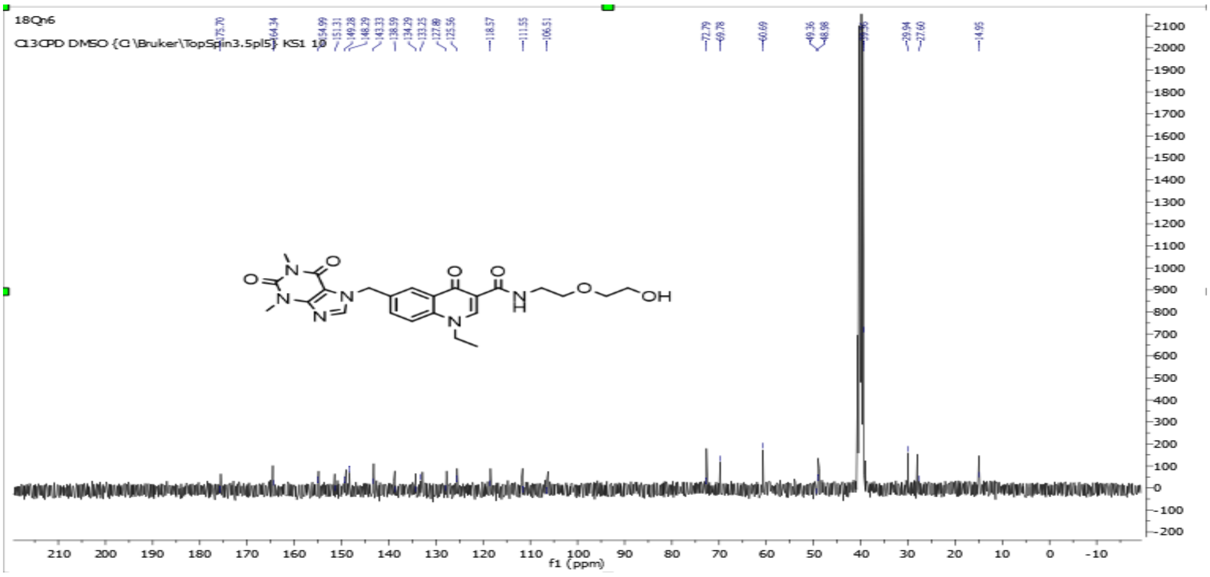
18Qn6
C13CPD DMSO {C:\Bruker\TopSpin3.5pl5} KS1 10
f1 (ppm)

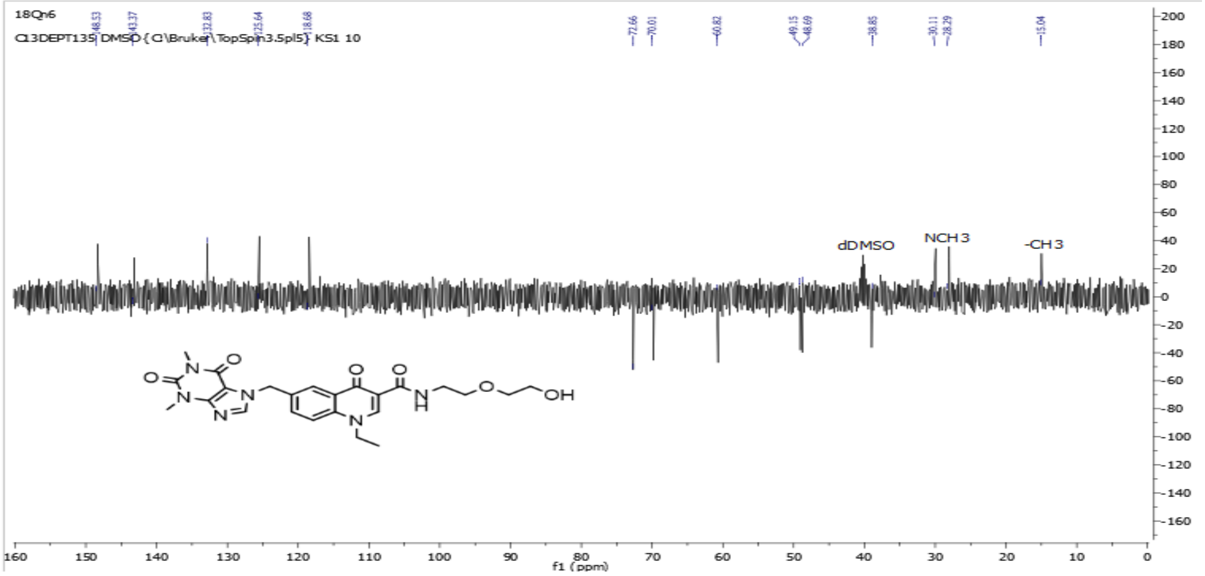
18Qn6
C13DEPT135 DMSO {C:\Bruker\TopSpin3.5pl5} KS1 10
dDMSO
NCH3
-CH3
f1 (ppm)

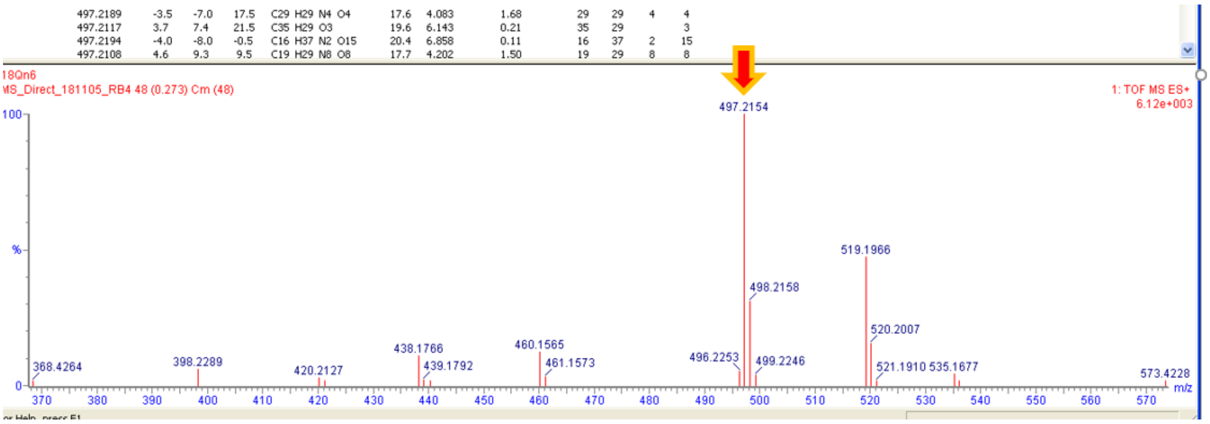

Dose-response against Mtb

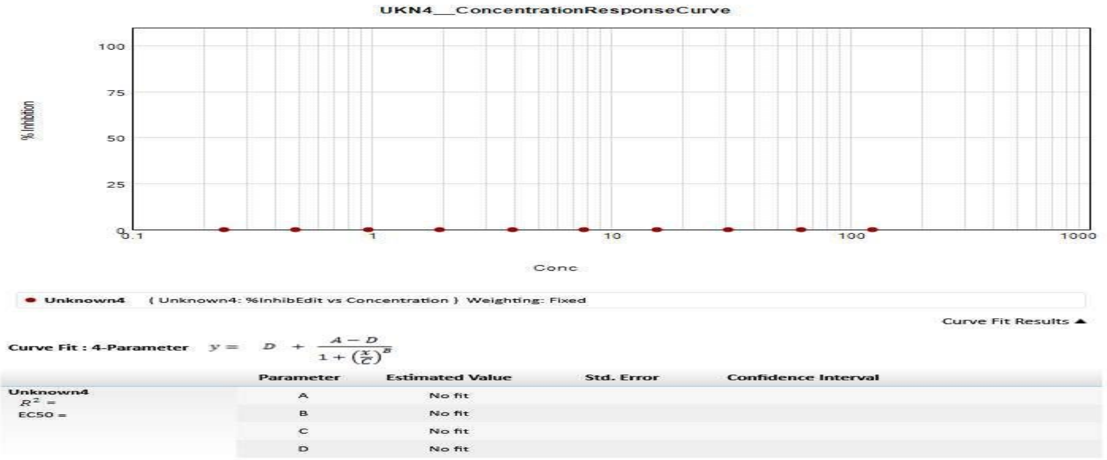

Compound 4c

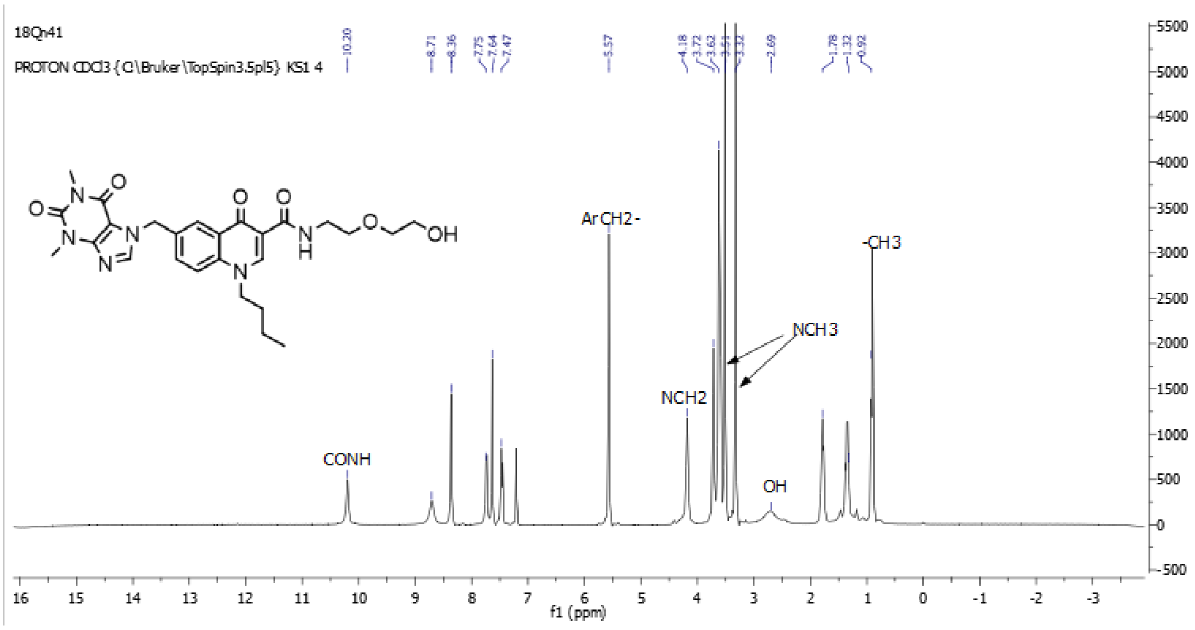

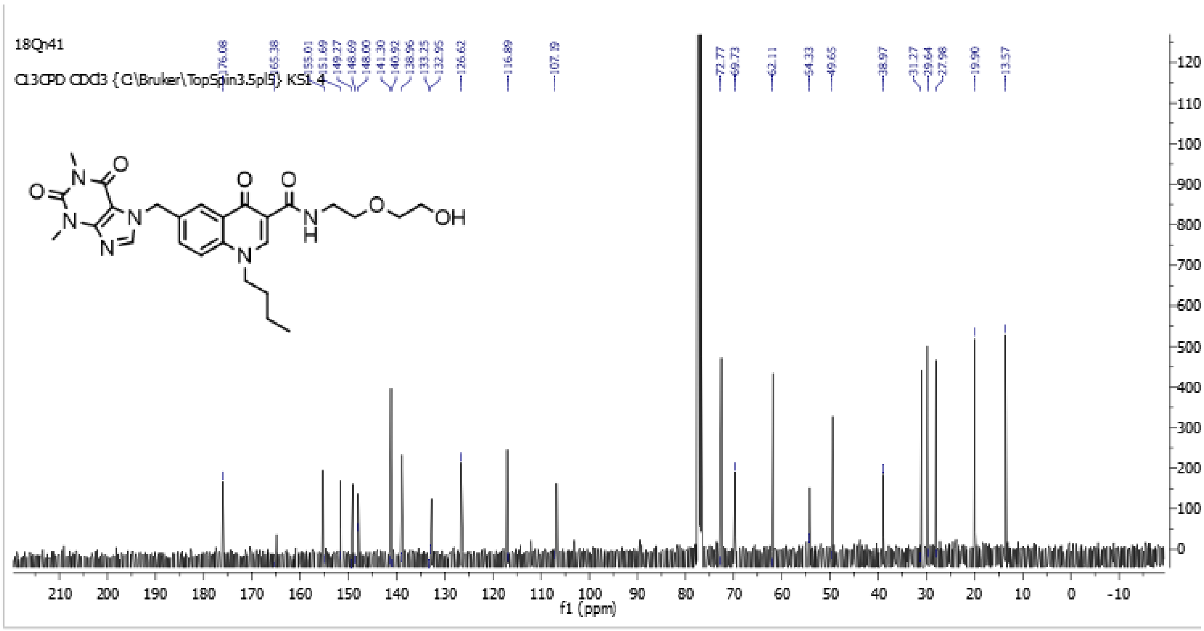
18Qn41
C13CPD CDCl3 {C:\Bruker\TopSpin3.5pl5} KS1 4
176.08
165.38
155.01
151.69
149.27
148.69
148.00
141.30
140.92
138.96
133.25
132.95
126.62
116.89
107.19
72.77
69.73
62.11
54.33
49.65
38.97
31.27
29.64
27.98
19.90
13.57
f1 (ppm)

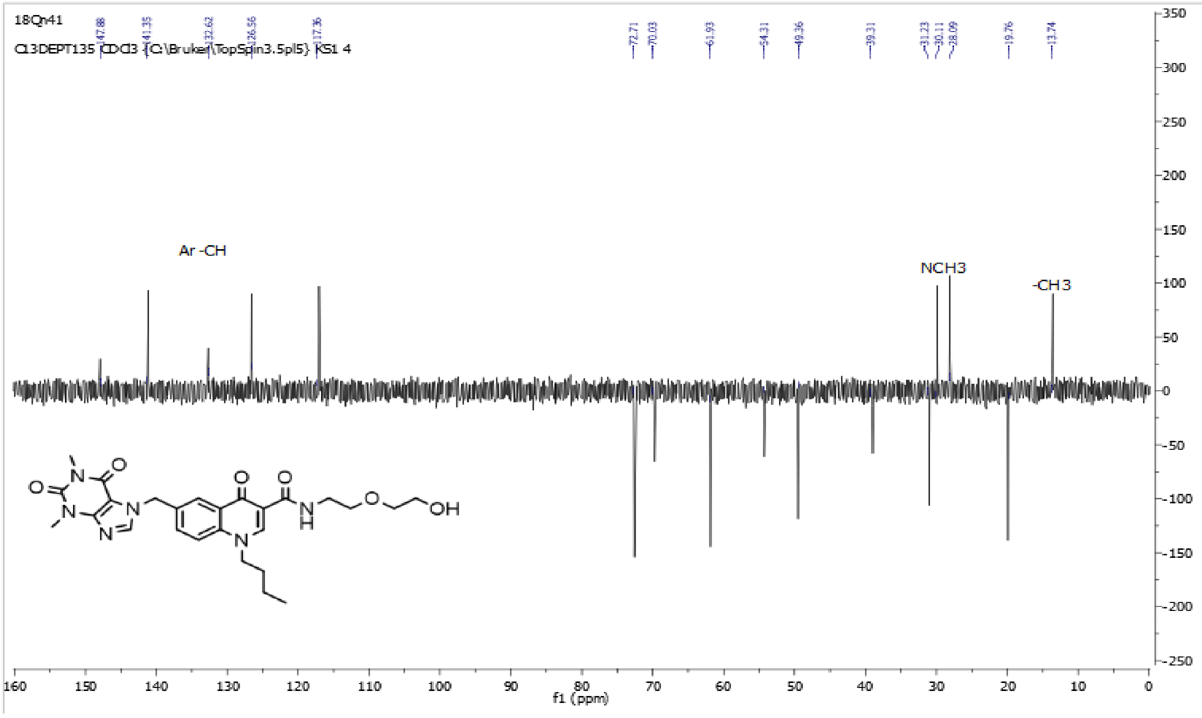
18Qn41
C13DEPT135 CDCl3 {C:\Bruker\TopSpin3.5pl5} KS1 4
147.88
141.35
132.62
126.56
117.36
72.71
70.03
61.93
54.31
49.36
39.31
31.23
30.11
28.09
19.76
13.74
Ar -CH
NCH3
-CH3
f1 (ppm)

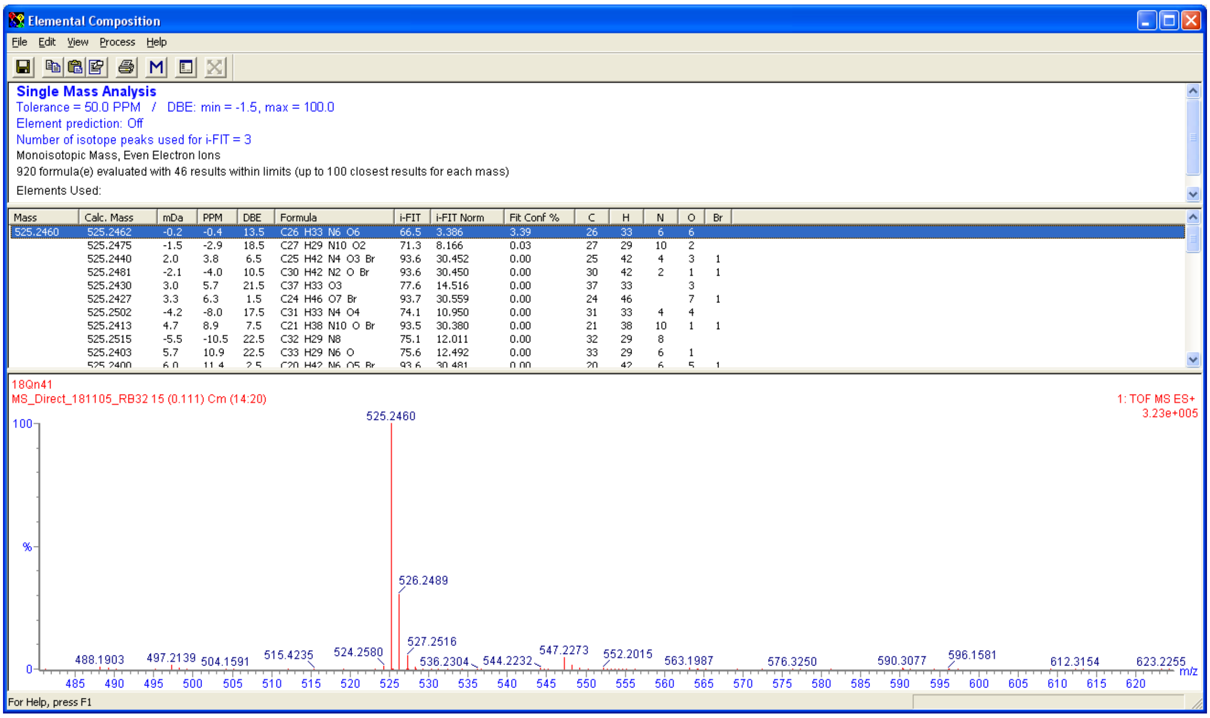

Dose-response against Mtb

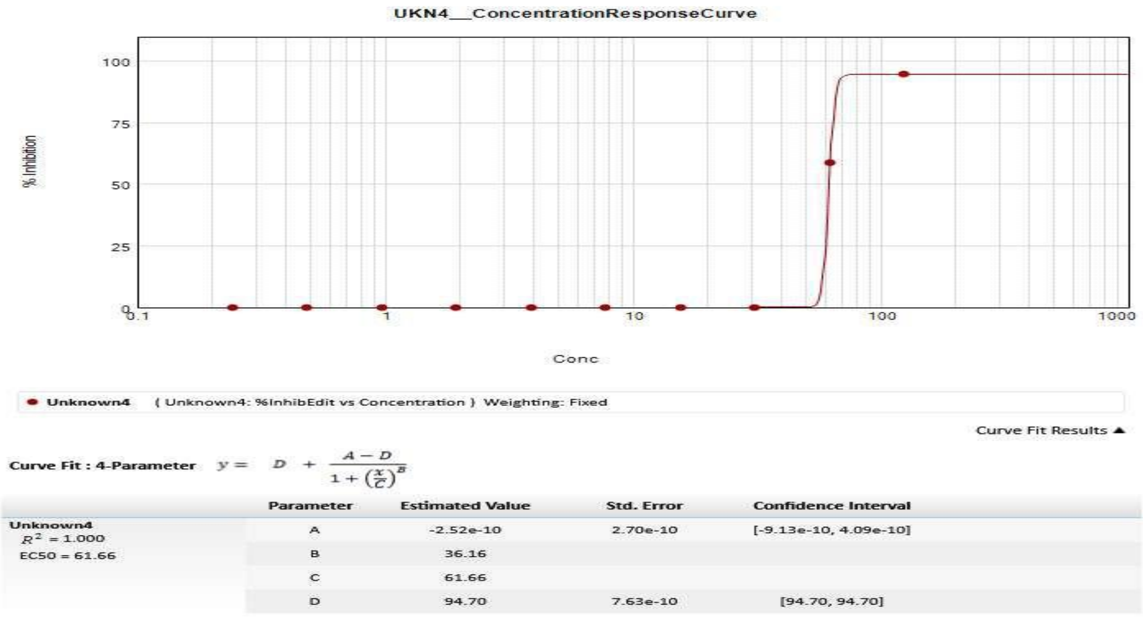

Compound 4d

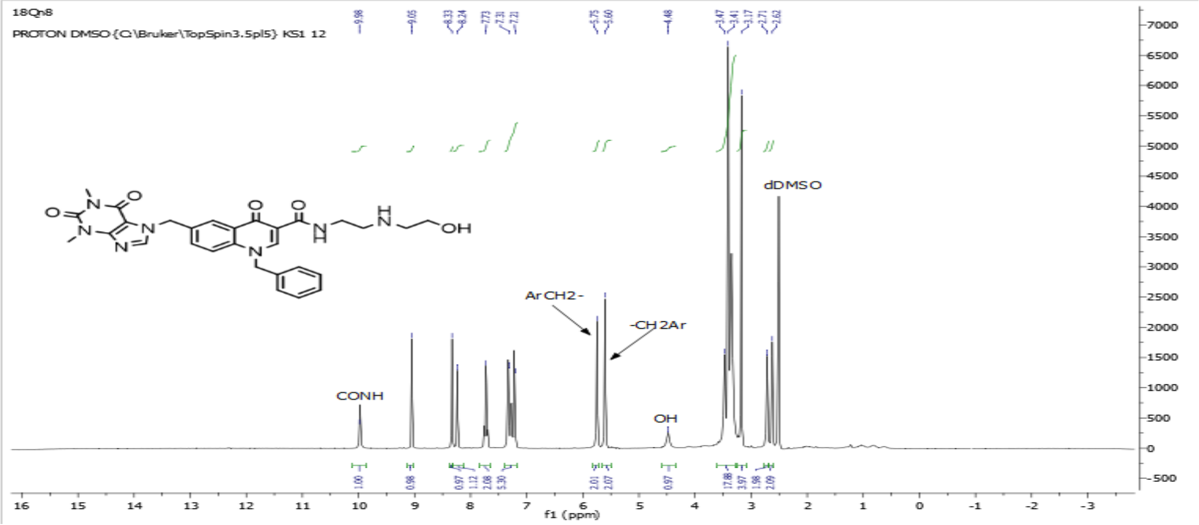
18Qn8
PROTON DMSO {C:\Bruker\TopSpin3.5pl5} KS1 12
dDMSO
ArCH2-
-CH2Ar
CONH
OH
f1 (ppm)

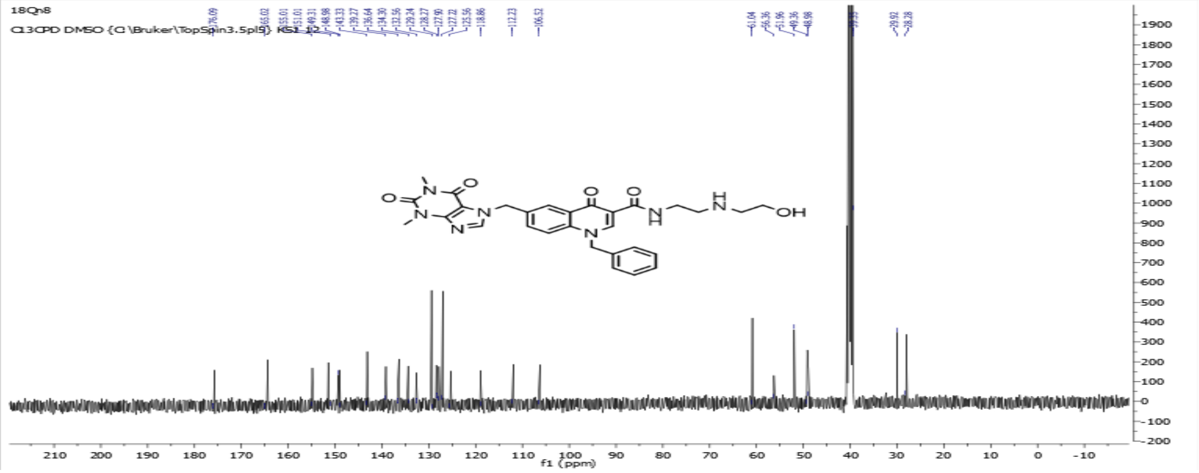
18Qn8
C13CPD DMSO {C:\Bruker\TopSpin3.5pl5} KS1 12
f1 (ppm)

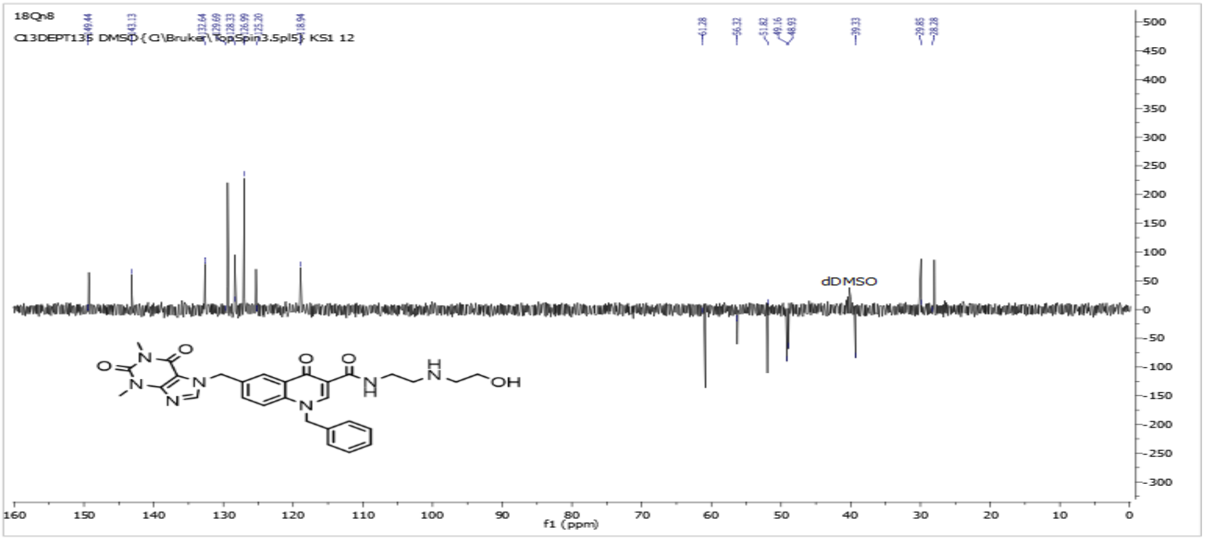
18Qn8
C13DEPT135 DMSO {C:\Bruker\TopSpin3.5pl5} KS1 12
dDMSO
f1 (ppm)

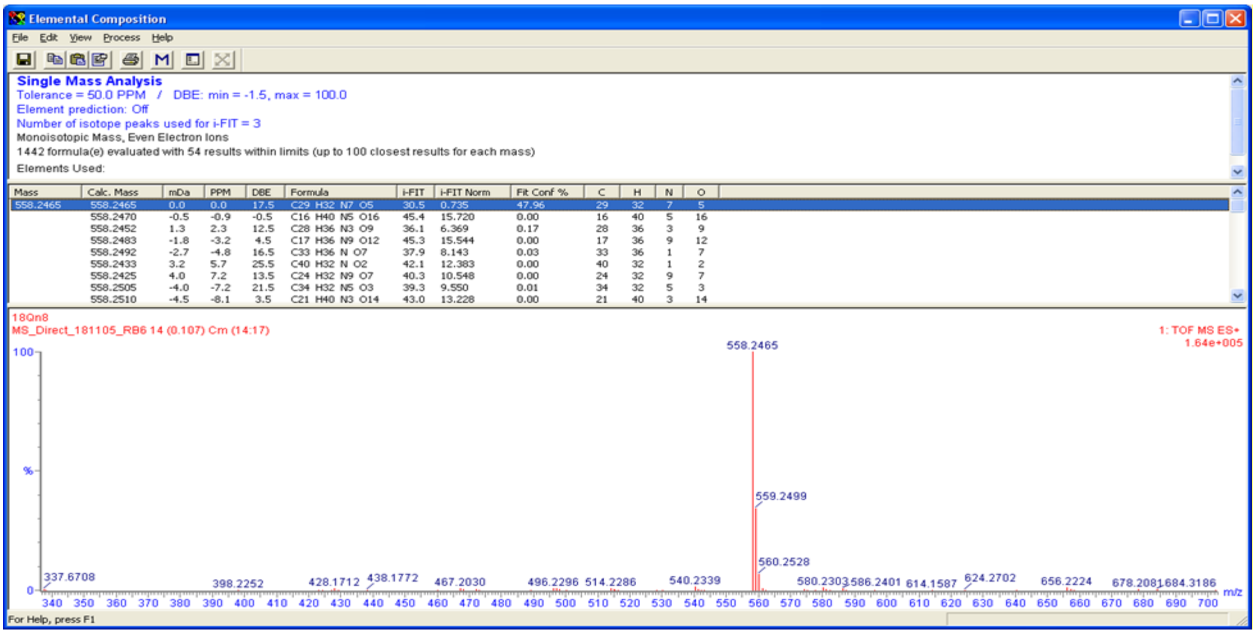

Dose-response against Mtb

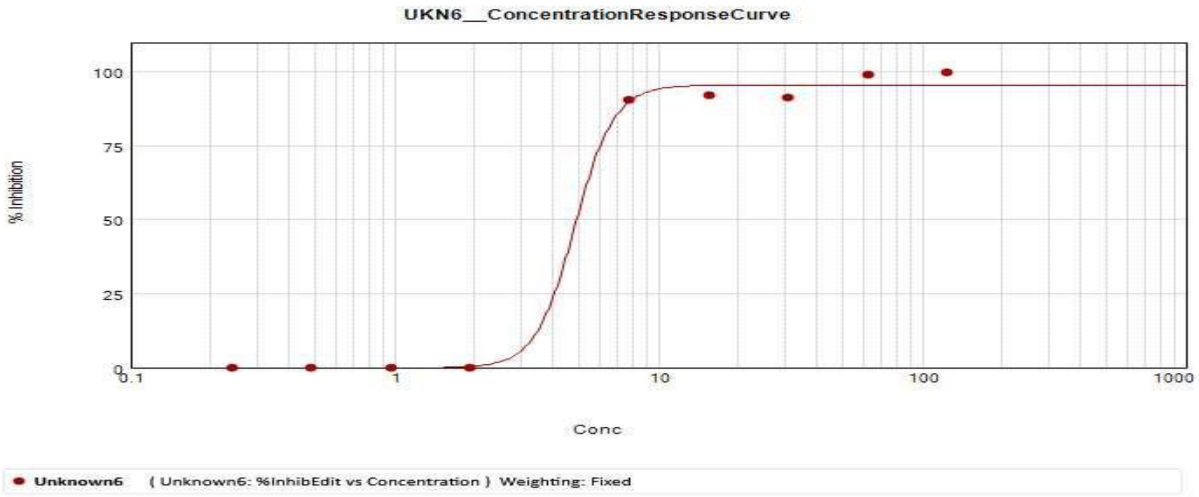

Curve Fit Results ▲

Curve Fit : 4-Parameter $y = D + \frac{A - D}{1 + \left(\frac{x}{C}\right)^B}$

	Parameter	Estimated Value	Std. Error	Confidence Interval
**Unknown6** R^2 = 0.997 EC50 = 4.812	A	-0.124	2.066	[-5.436, 5.188]
	B	5.839	6.087	[-9.808, 21.49]
	C	4.812	2.452	[-1.491, 11.12]
	D	95.58	1.792	[90.97, 100.2]

Compound 4e

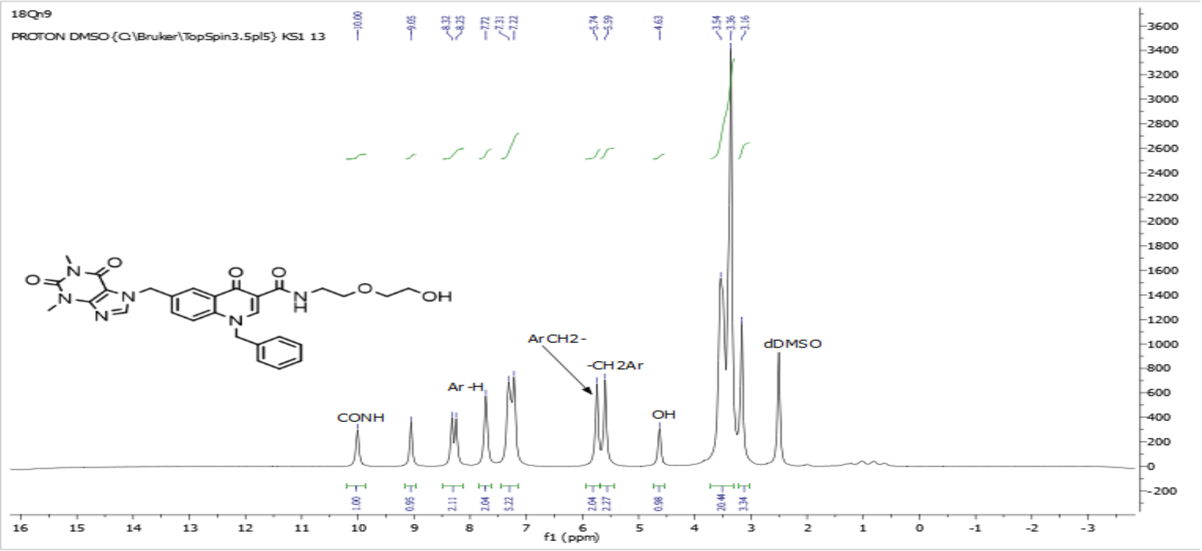
18Qn9
PROTON DMSO {C:\Bruker\TopSpin3.5pl5} KS1 13
CONH
Ar-H
ArCH2-
-CH2Ar
OH
dDMSO
OH
f1 (ppm)

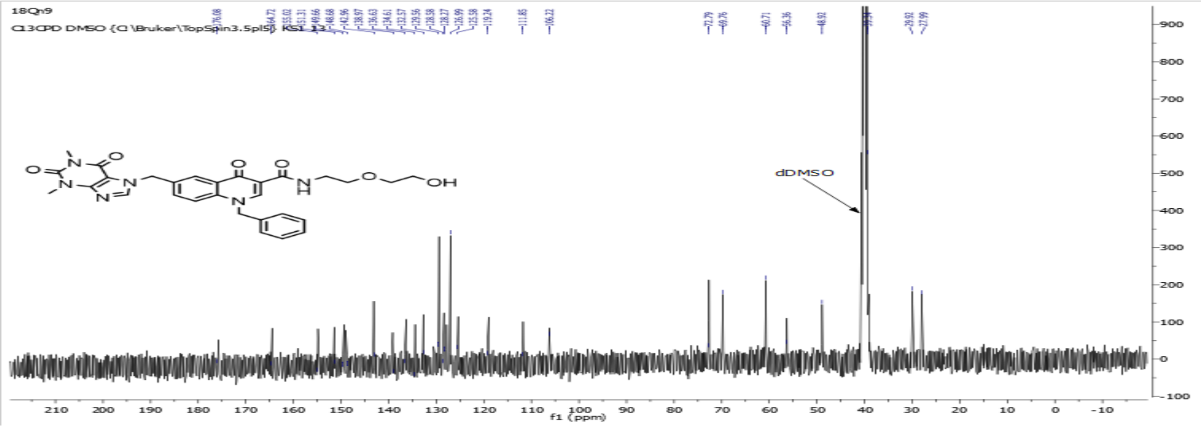
18Qn9
C13CPD DMSO {C:\Bruker\TopSpin3.5pl5} KS1 13
dDMSO
OH
f1 (ppm)

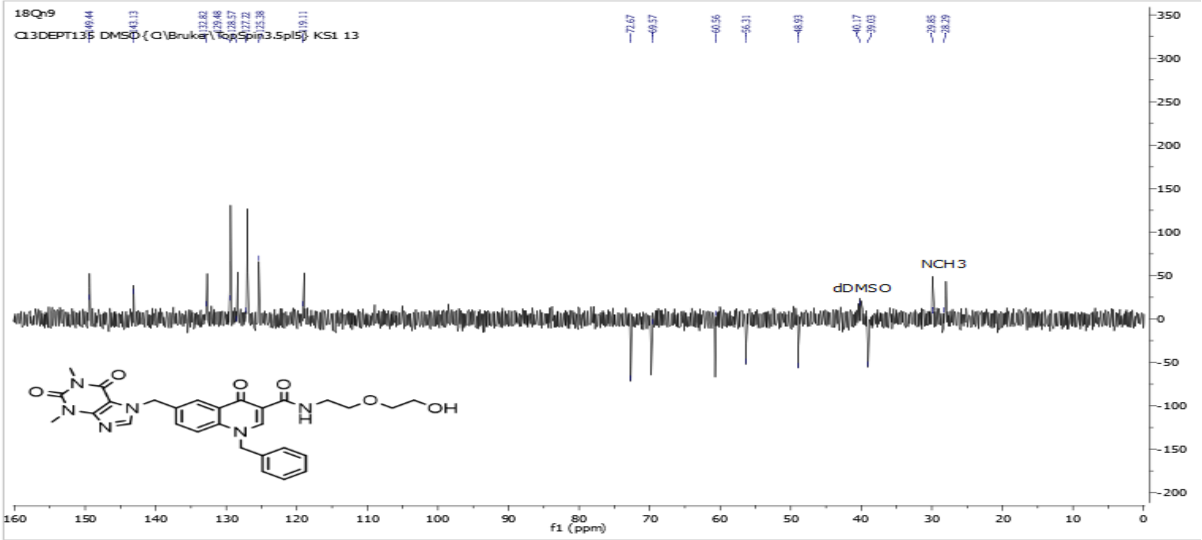
18Qn9
C13DEPT135 DMSO {C:\Bruker\TopSpin3.5pl5} KS1 13
NCH3
dDMSO
OH
f1 (ppm)

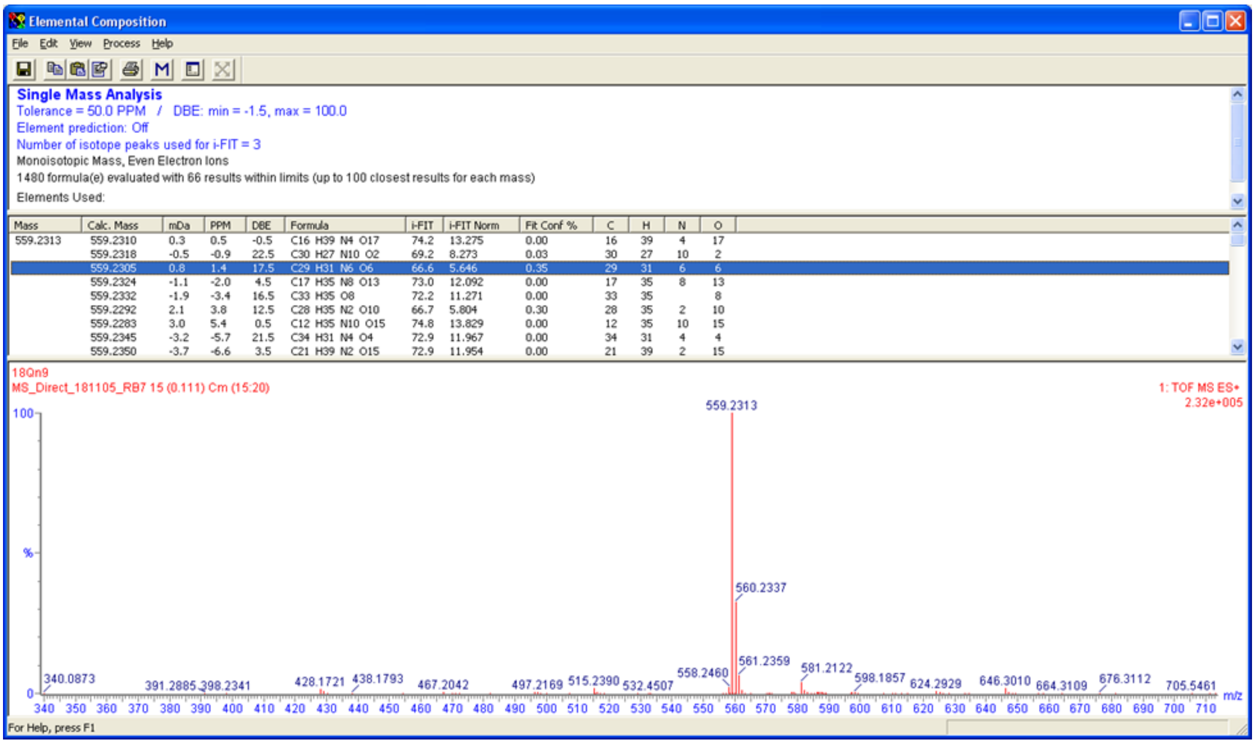

Dose-response against Mtb

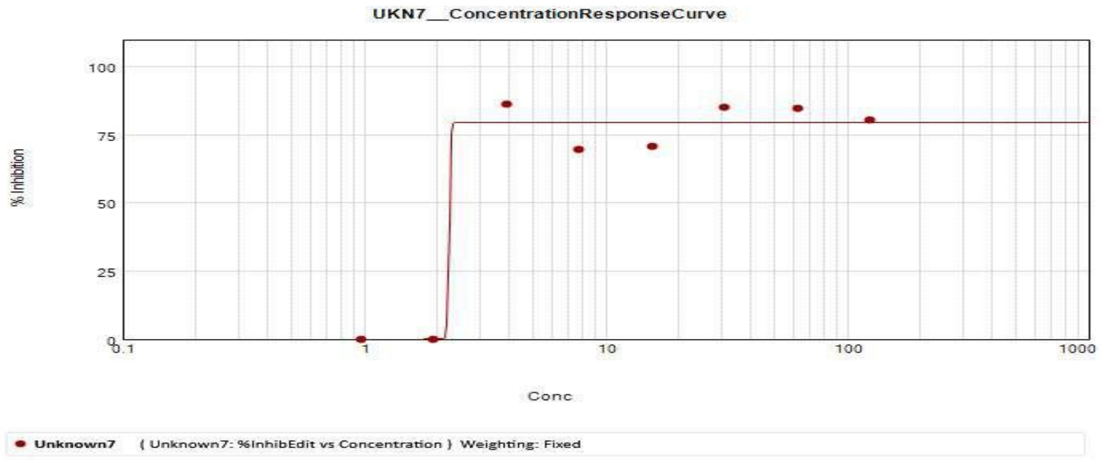

Curve Fit Results ▲

Curve Fit : 4-Parameter $y = D + \frac{A - D}{1 + \left(\frac{x}{C}\right)^B}$

	Parameter	Estimated Value	Std. Error	Confidence Interval
Unknown7 R^2 = 0.971 EC50 = 2.246	A	5.80e-15	8.456	[-23.48, 23.48]
	B	147.1		
	C	2.246		
	D	79.43	3.452	[69.84, 89.01]

Compound 4f

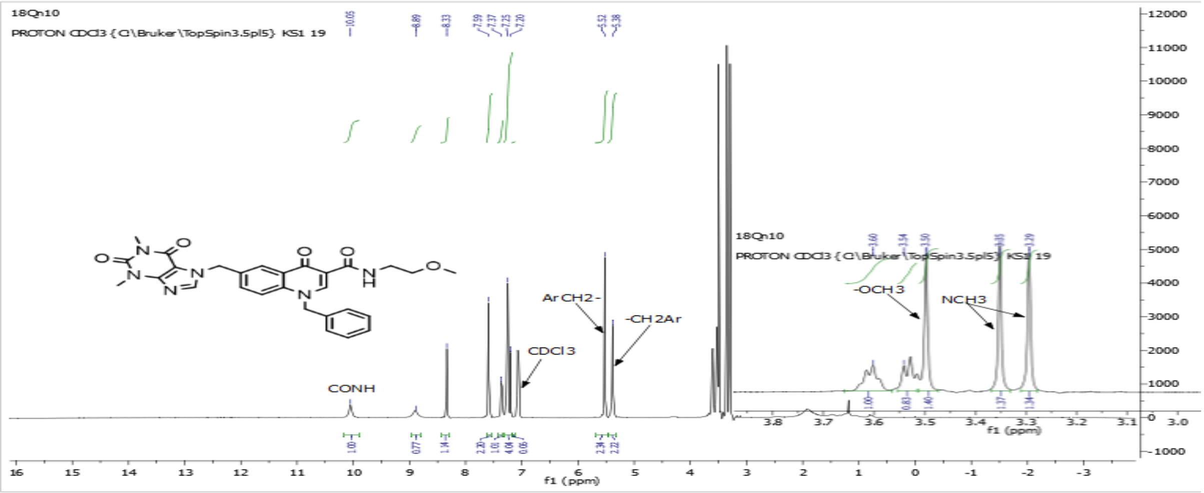
18Qn10
PROTON CDCl3 {C:\Bruker\TopSpin3.5pl5} KS1 19
CONH
CDCl3
ArCH2-
-CH2Ar
-OCH3
NCH3
f1 (ppm)

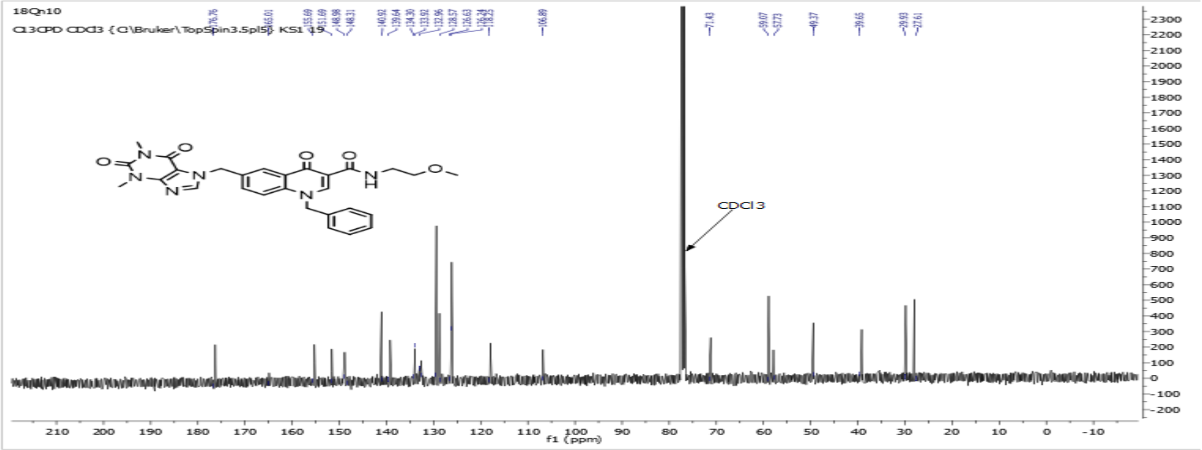
18Qn10
C13CPD CDCl3 {C:\Bruker\TopSpin3.5pl5} KS1 19
CDCl3
f1 (ppm)

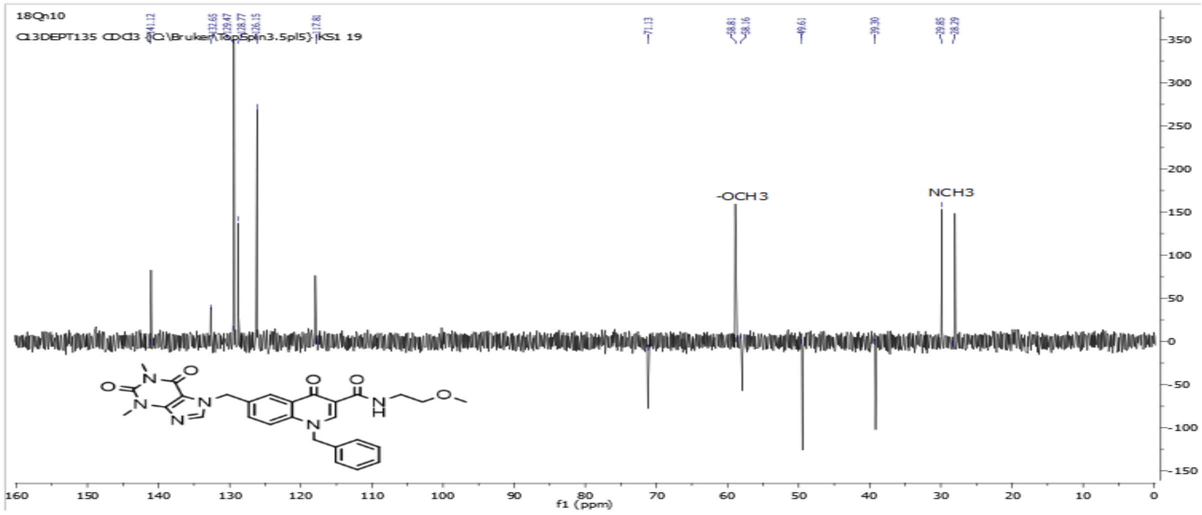
18Qn10
C13DEPT135 CDCl3 {C:\Bruker\TopSpin3.5pl5} KS1 19
-OCH3
NCH3
f1 (ppm)

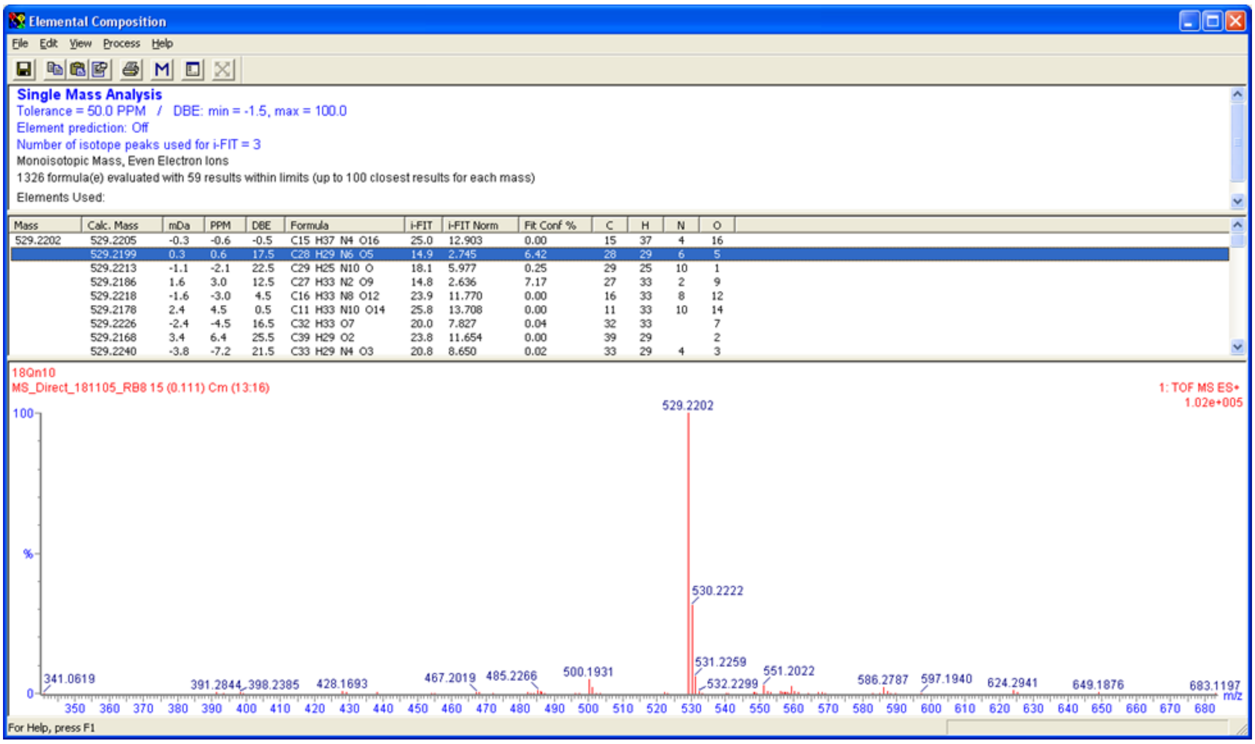

Dose-response against Mtb

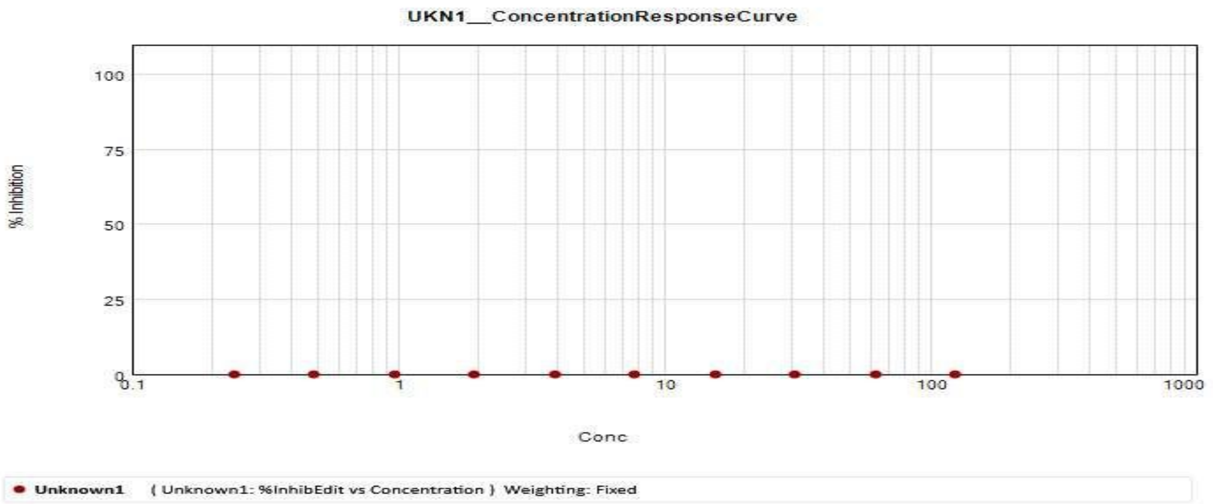

Curve Fit Results ▲

Curve Fit : 4-Parameter $y = D + \frac{A - D}{1 + \left(\frac{x}{C}\right)^B}$

	Parameter	Estimated Value	Std. Error	Confidence Interval
Unknown1 R^2 = EC50 =	A	No fit		
	B	No fit		
	C	No fit		
	D	No fit		

Compound 4g

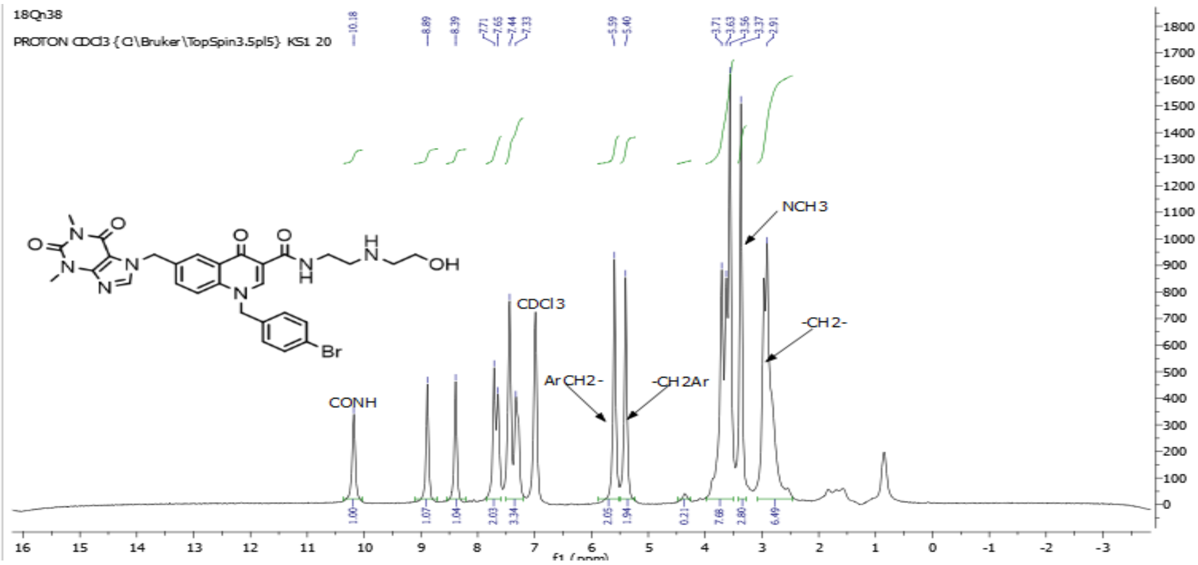
18Qn38
PROTON CDCl3 {C:\Bruker\TopSpin3.5pl5} KS1 20
CONH
CDCl3
ArCH2-
-CH2Ar
NCH3
-CH2-
Br
OH
f1 (ppm)

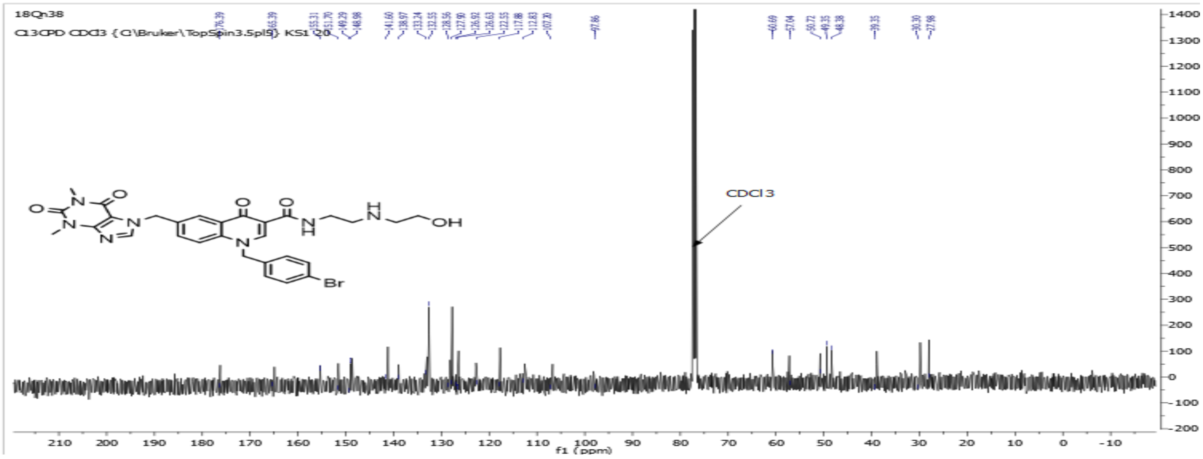
18Qn38
C13CPD CDCl3 {C:\Bruker\TopSpin3.5pl5} KS1 20
CDCl3
Br
OH
f1 (ppm)

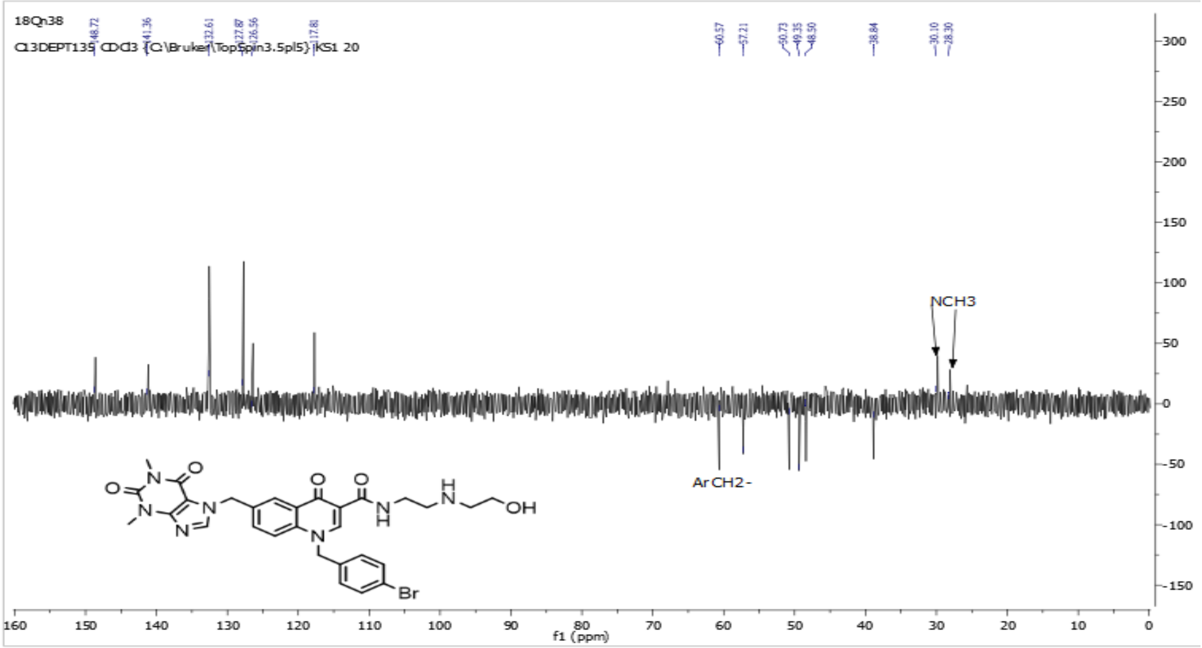
18Qn38
C13DEPT135 CDCl3 {C:\Bruker\TopSpin3.5pl5} KS1 20
NCH3
ArCH2-
Br
OH
f1 (ppm)

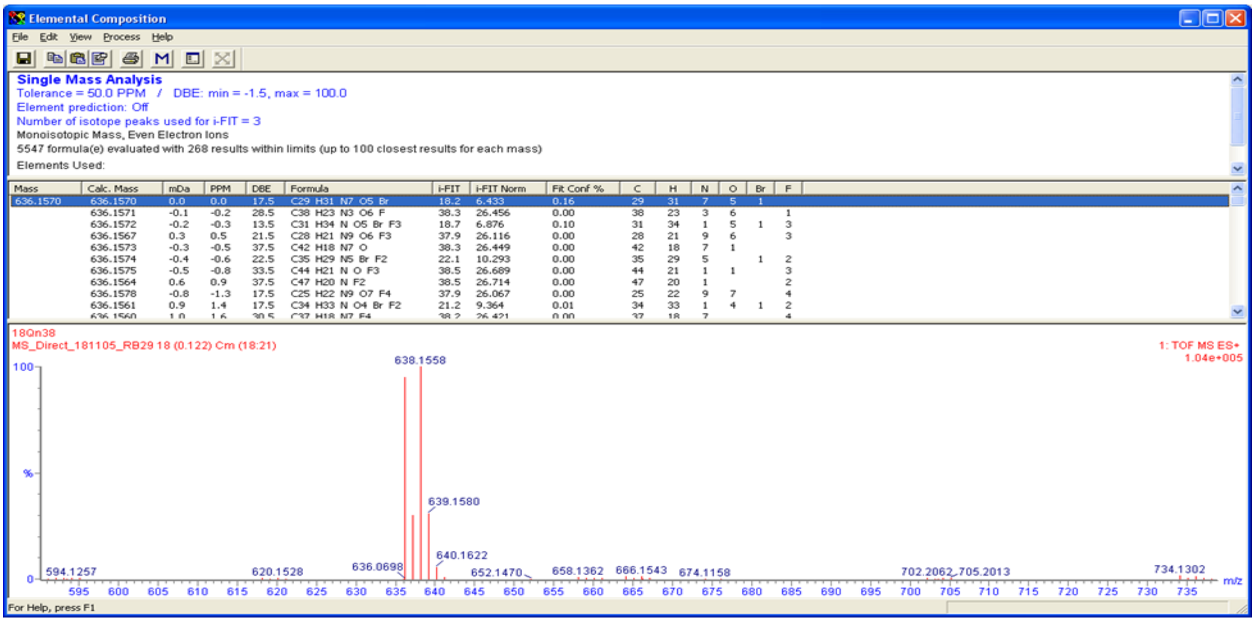

Dose-response against Mtb

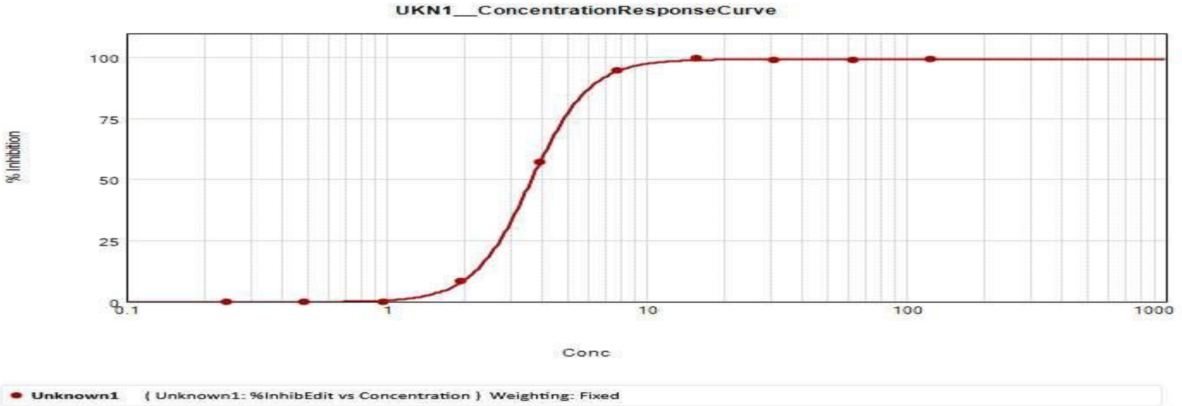

Curve Fit : 4-Parameter $y = D + \frac{A - D}{1 + \left(\frac{x}{C}\right)^{B}}$

	Parameter	Estimated Value	Std. Error	Confidence Interval
Unknown1<br>R^2 = 1.000<br>EC50 = 3.604	A	-0.155	0.248	[-0.761, 0.451]
	B	3.892	0.079	[3.699, 4.085]
	C	3.604	0.017	[3.562, 3.647]
	D	99.34	0.202	[98.84, 99.83]

Compound 4h

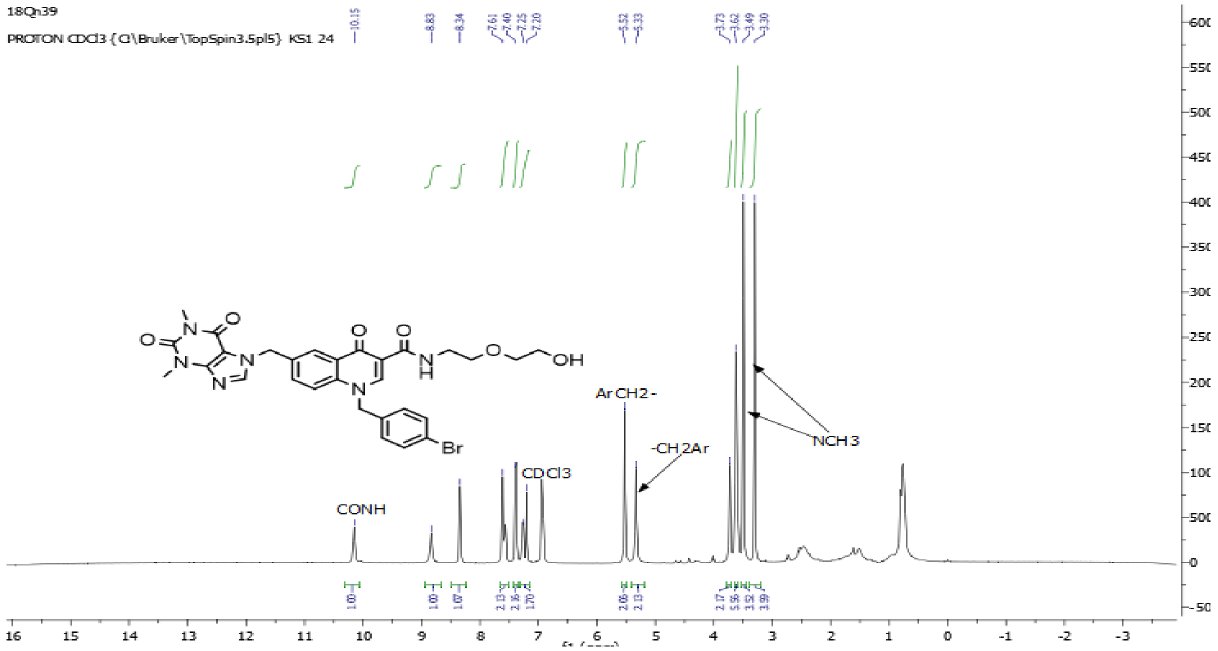
18Qn39
PROTON CDCl3 {C:\Bruker\TopSpin3.5pl5} KS1 24
CONH
ArCH2-
-CH2Ar
CDCl3
NCH3
Br
OH

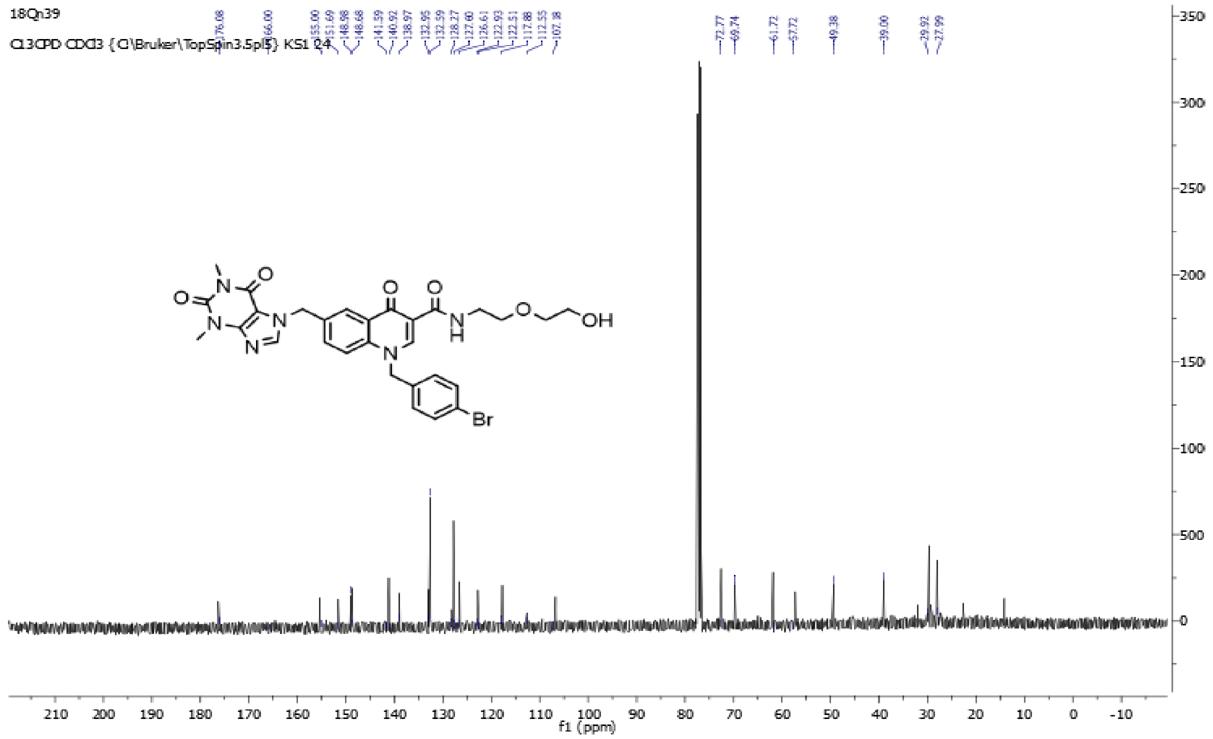
18Qn39
C13CPD CDCl3 {C:\Bruker\TopSpin3.5pl5} KS1 24
Br
OH
f1 (ppm)

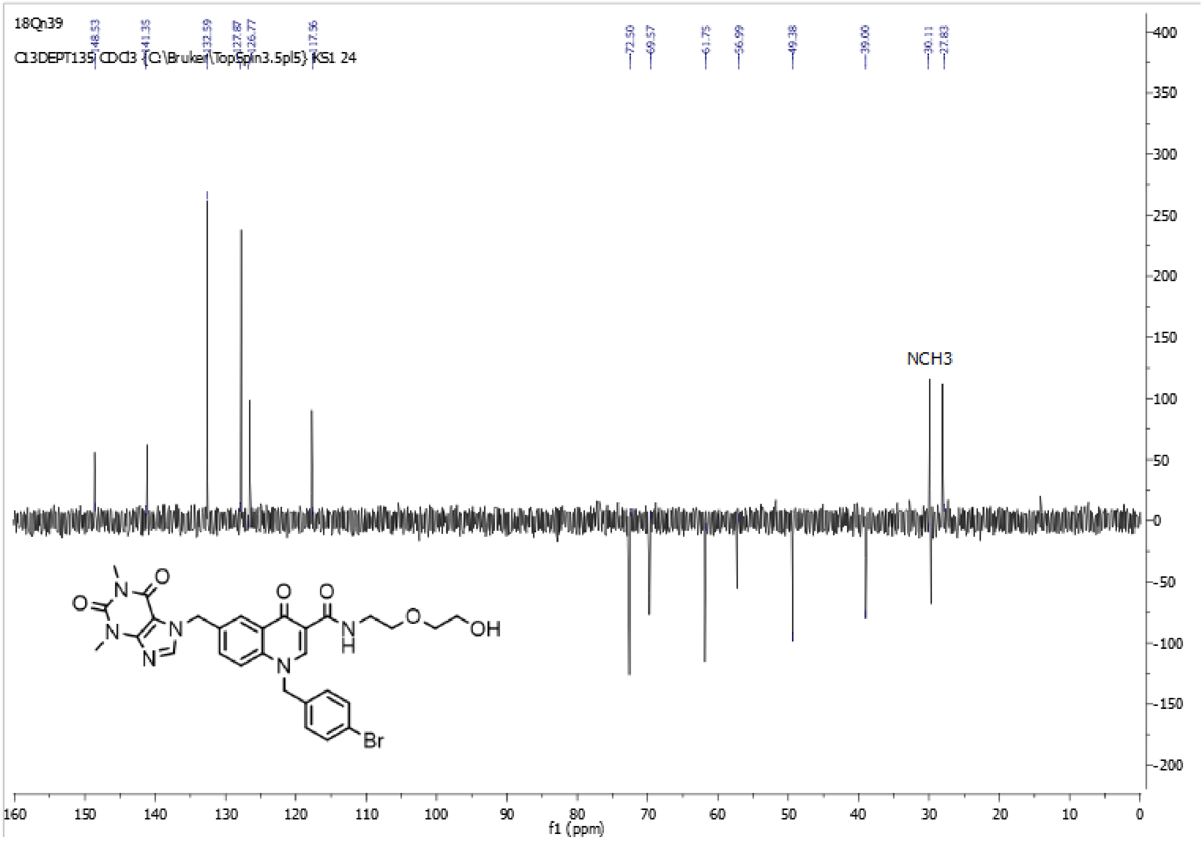

Elemental Composition

File Edit View Process Help

Single Mass Analysis

Tolerance = 50.0 PPM / DBE: min = -1.5, max = 100.0

Element prediction: Off

Number of isotope peaks used for i-FIT = 3

Monoisotopic Mass, Even Electron Ions

1216 formula(e) evaluated with 64 results within limits (up to 100 closest results for each mass)

Elements Used:

Mass	Calc. Mass	mDa	PPM	DBE	Formula	i-FIT	i-FIT Norm	Fit Conf %	C	H	N	O	Br
637.1395	637.1400	-0.5	-0.8	32.5	C41 H21 N2 O6	35.8	21.519	0.00	41	21	2	6	
	637.1410	-1.5	-2.4	17.5	C29 H30 N6 O6 Br	19.6	5.305	0.50	29	30	6	6	1
	637.1378	1.7	2.7	25.5	C40 H30 O3 Br	20.6	6.404	0.17	40	30		3	1
	637.1413	-1.8	-2.8	37.5	C42 H17 N6 O2	35.8	21.533	0.00	42	17	6	2	
	637.1373	2.2	3.5	33.5	C37 H17 N8 O4	35.7	21.437	0.00	37	17	8	4	
	637.1424	-2.9	-4.6	22.5	C30 H26 N10 O2 Br	16.7	2.414	8.94	30	26	10	2	1
	637.1351	4.4	6.9	26.5	C36 H26 N6 O Br	19.5	5.293	0.50	36	26	6	1	1
	637.1440	-4.5	-7.1	36.5	C46 H21 O4	35.9	21.616	0.00	46	21		4	
	637.1341	5.4	8.5	41.5	C48 H17 N2 O	35.9	21.625	0.00	48	17	2	1	
	637.1450	-5.5	-8.6	21.5	C34 H30 N4 O4 Br	16.4	2.186	11.23	34	30	4	4	1
	637.1338	5.7	8.9	21.5	C35 H30 N2 O5 Br	16.9	2.622	7.27	35	30	2	5	1

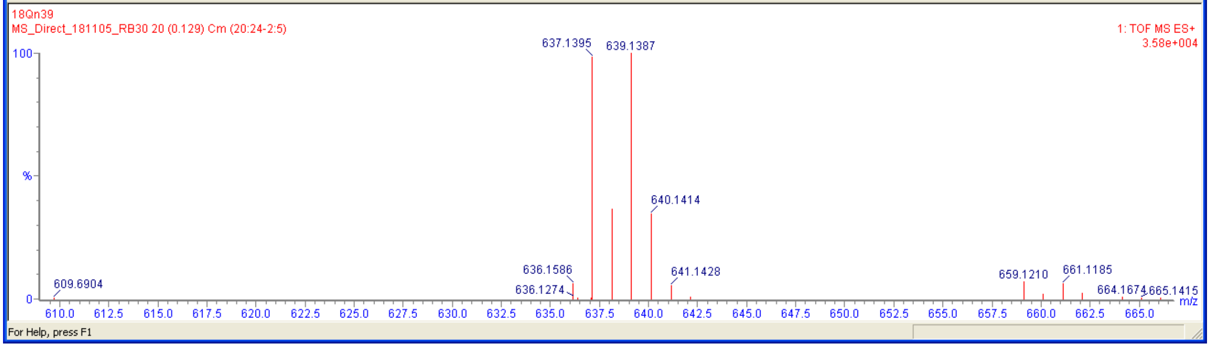

For Help, press F1

Dose-response against Mtb

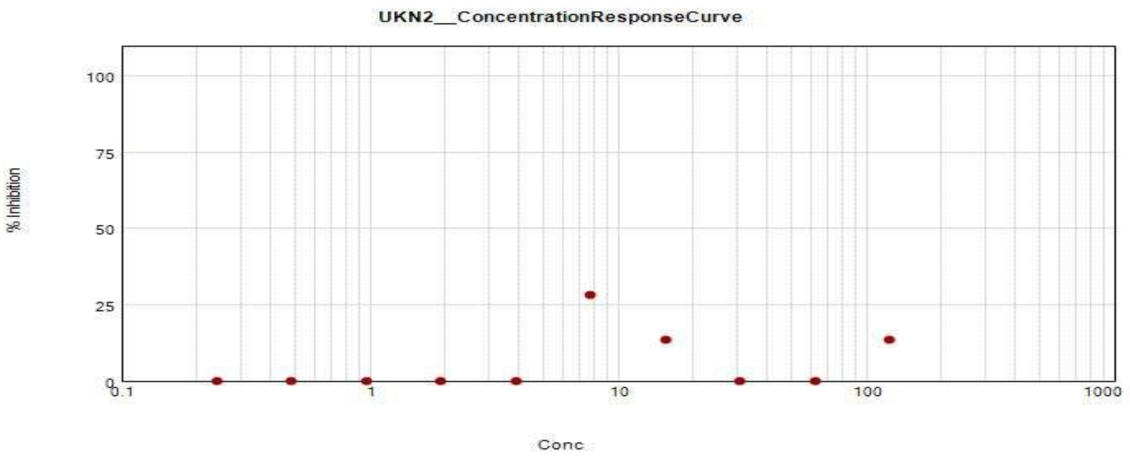

● **Unknown2** (Unknown2: %InhibEdit vs Concentration) Weighting: Fixed

Curve Fit Results ▲

Curve Fit : 4-Parameter $y = D + \frac{A - D}{1 + \left(\frac{x}{C}\right)^B}$

	Parameter	Estimated Value	Std. Error	Confidence Interval
**Unknown2** R^2 = EC50 =	A	No fit		
	B	No fit		
	C	No fit		
	D	No fit		

Compound 7a

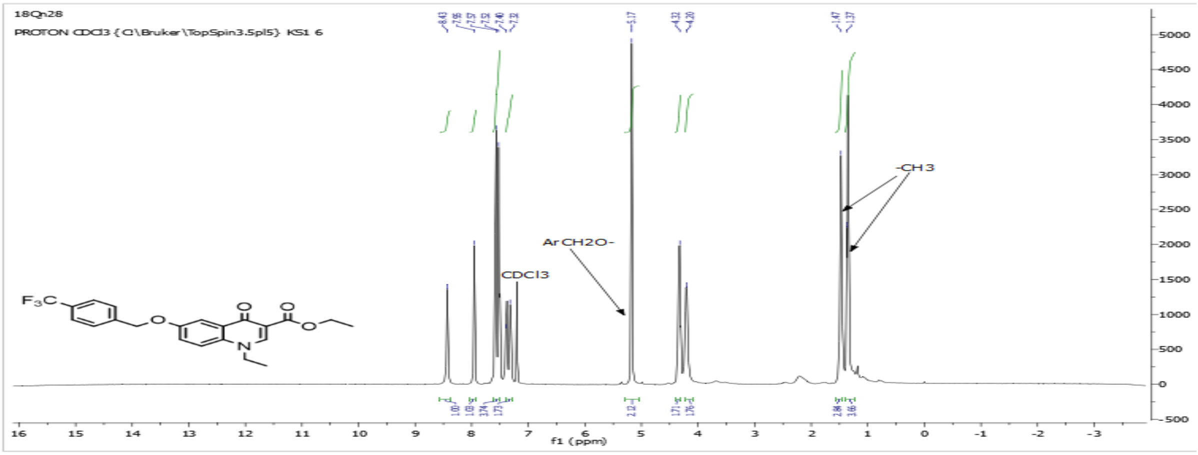

18Qn28
PROTON CDCl3 {C:\Bruker\TopSpin3.5pl5} KS1 6
ArCH2O-
CDCl3
-CH3
f1 (ppm)

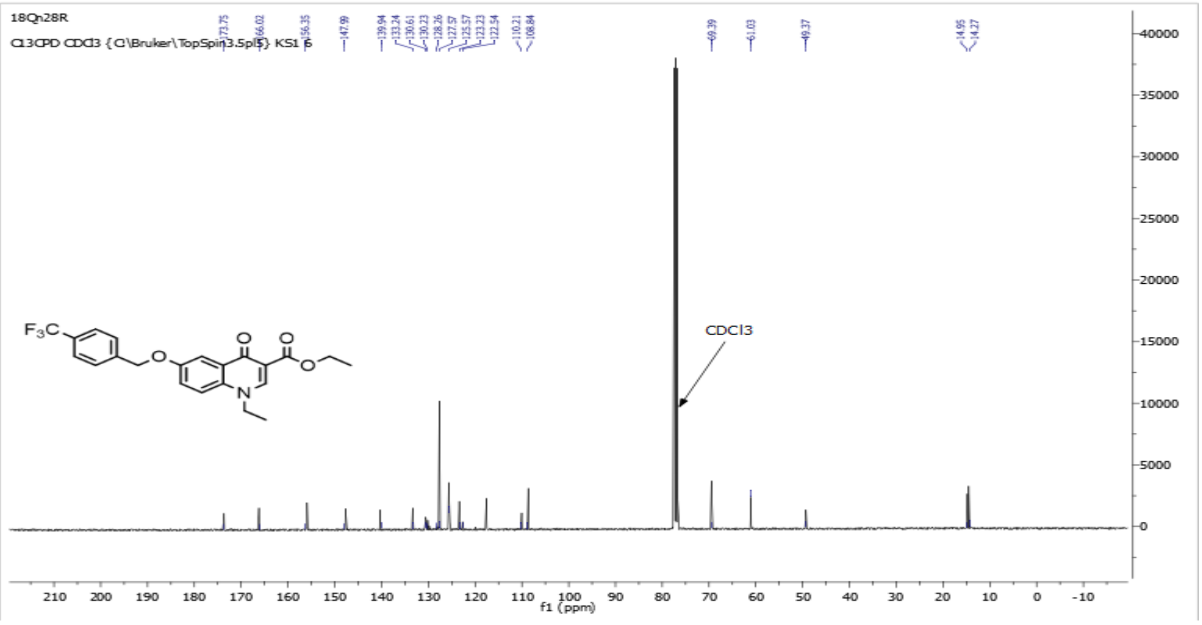

18Qn28R
C13CPD CDCl3 {C:\Bruker\TopSpin3.5pl5} KS1 6
CDCl3
f1 (ppm)

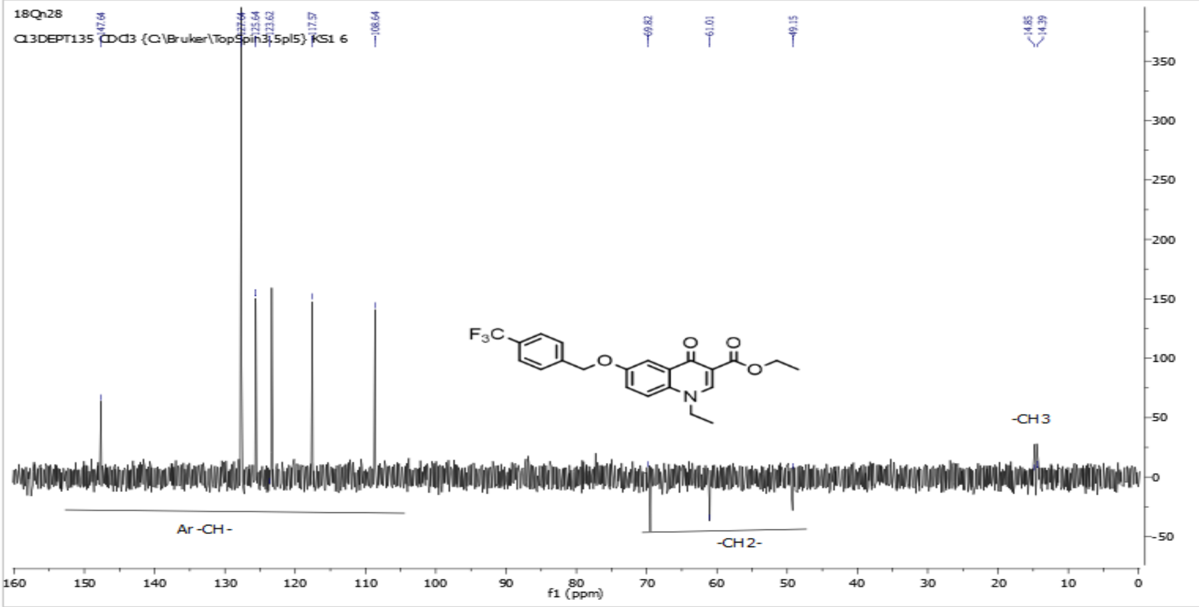

18Qn28
C13DEPT135 CDCl3 {C:\Bruker\TopSpin3.5pl5} KS1 6
-CH3
Ar-CH-
-CH2-
f1 (ppm)

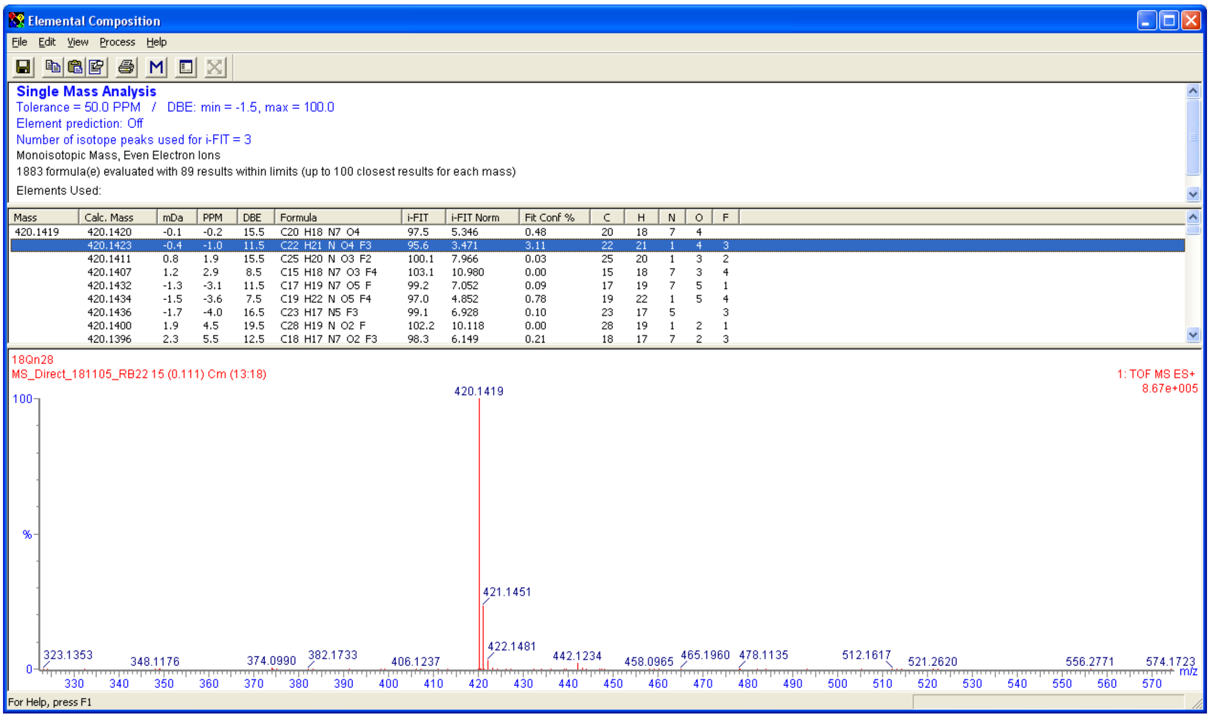

Dose-response against Mtb

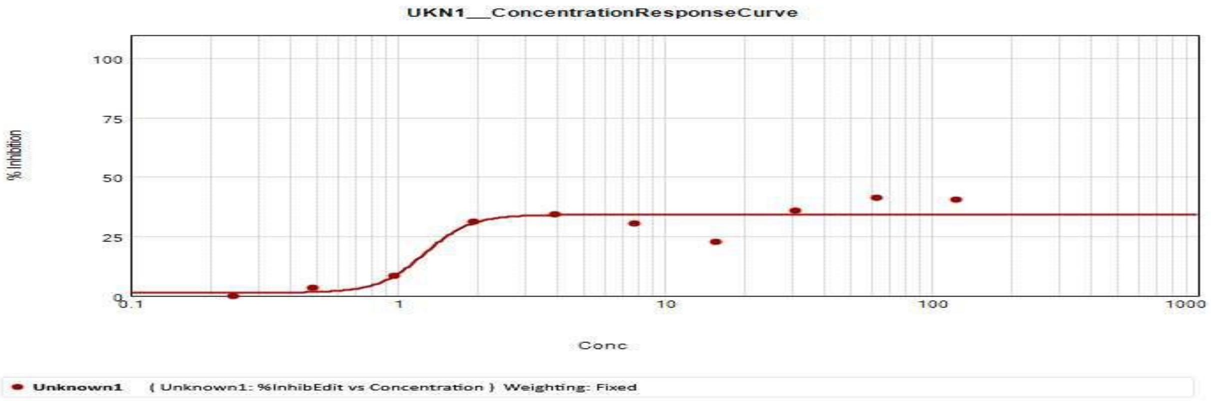

Curve Fit Results ▲

Curve Fit : 4-Parameter $y = D + \frac{A - D}{1 + \left(\frac{x}{C}\right)^B}$

	Parameter	Estimated Value	Std. Error	Confidence Interval
Unknown1<br>R^2 = 0.888<br>EC50 = 1.260	A	1.354	4.692	[-10.13, 12.84]
	B	4.992	3.757	[-4.201, 14.18]
	C	1.260	0.306	[0.512, 2.007]
	D	34.22	2.618	[27.81, 40.62]

Compound 7b

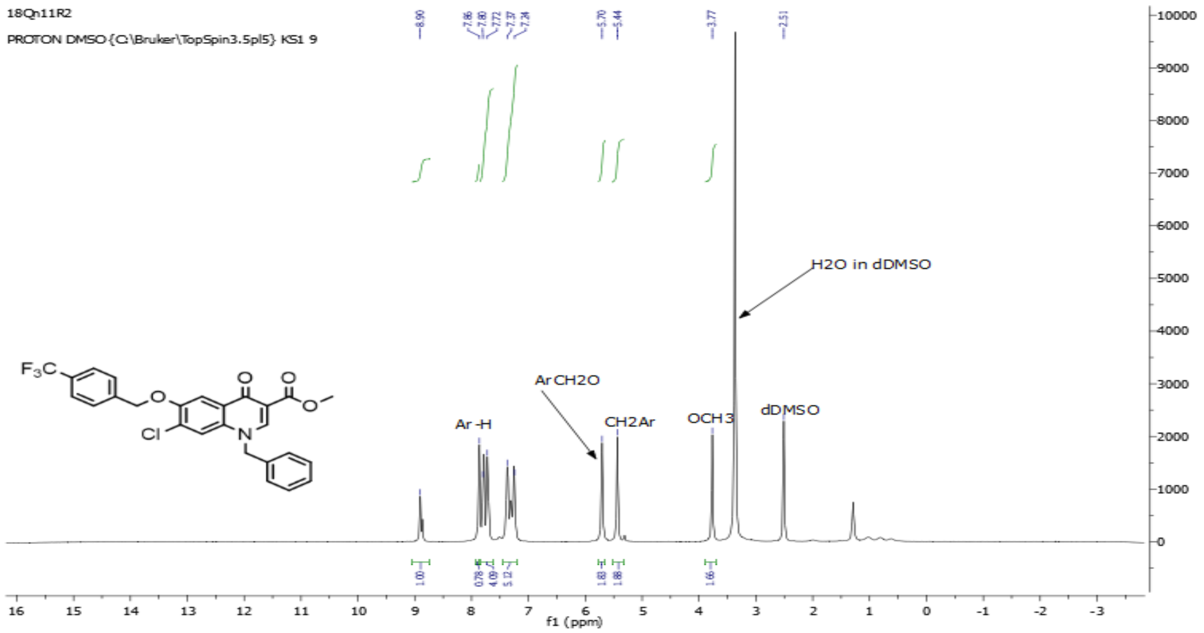
18Qn11R2
PROTON DMSO {C:\Bruker\TopSpin3.5pl5} KS1 9
H2O in dDMSO
Ar CH2O
Ar -H
CH2Ar
OCH3
dDMSO
f1 (ppm)

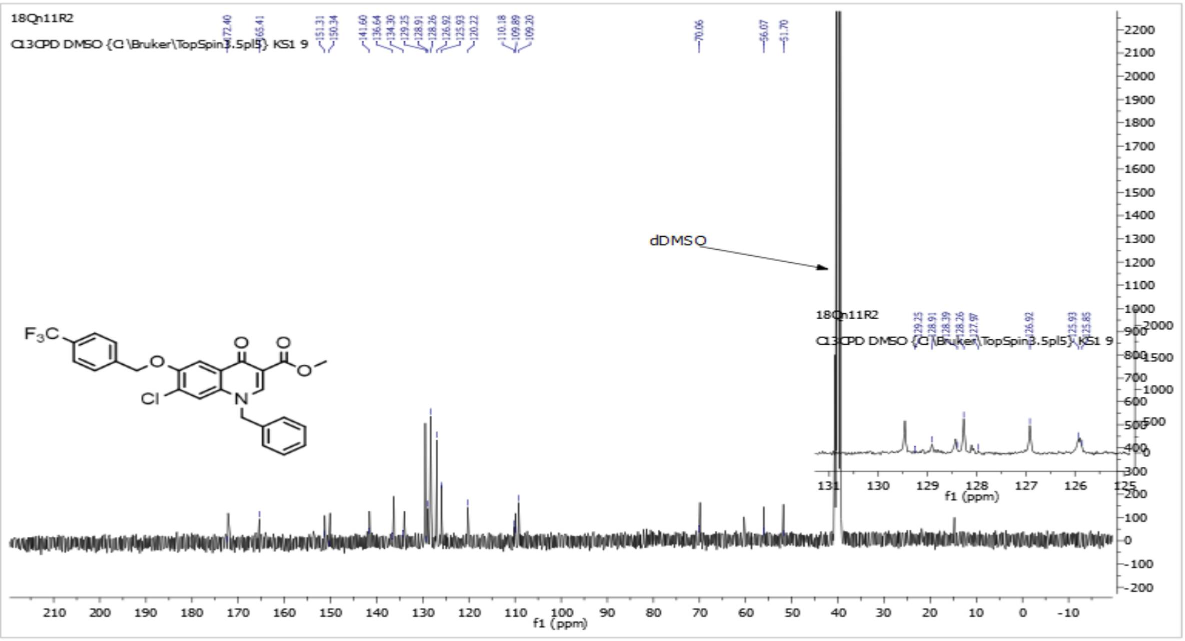
18Qn11R2
C13CPD DMSO {C:\Bruker\TopSpin3.5pl5} KS1 9
dDMSO
f1 (ppm)

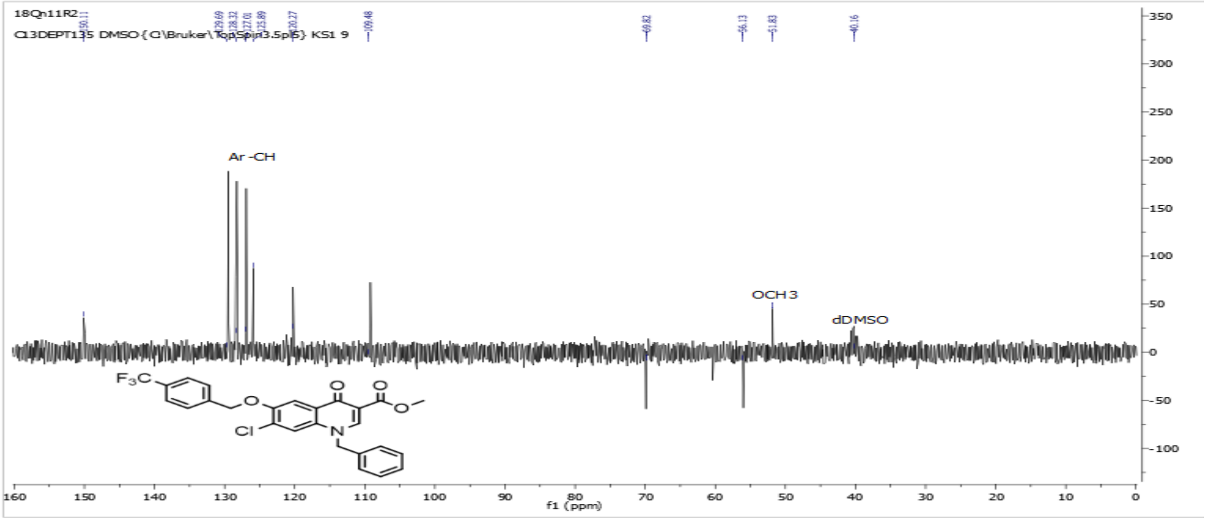

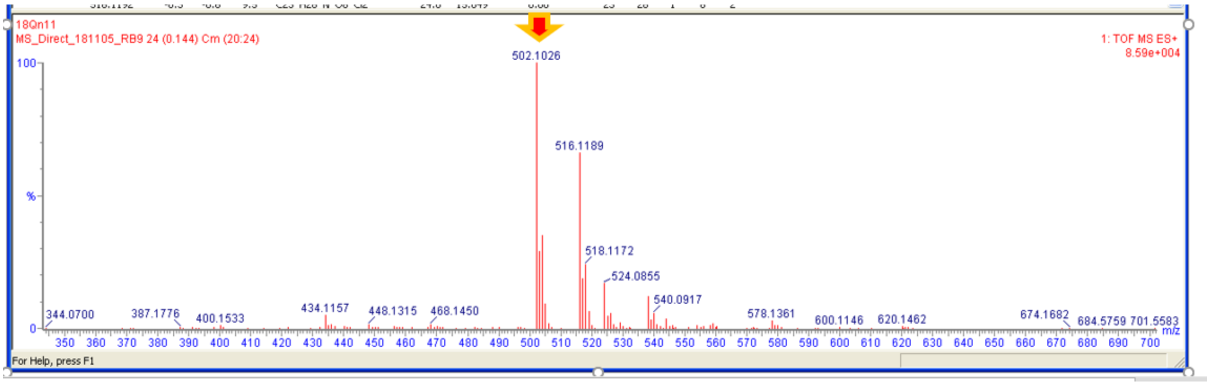

Dose-response against Mtb

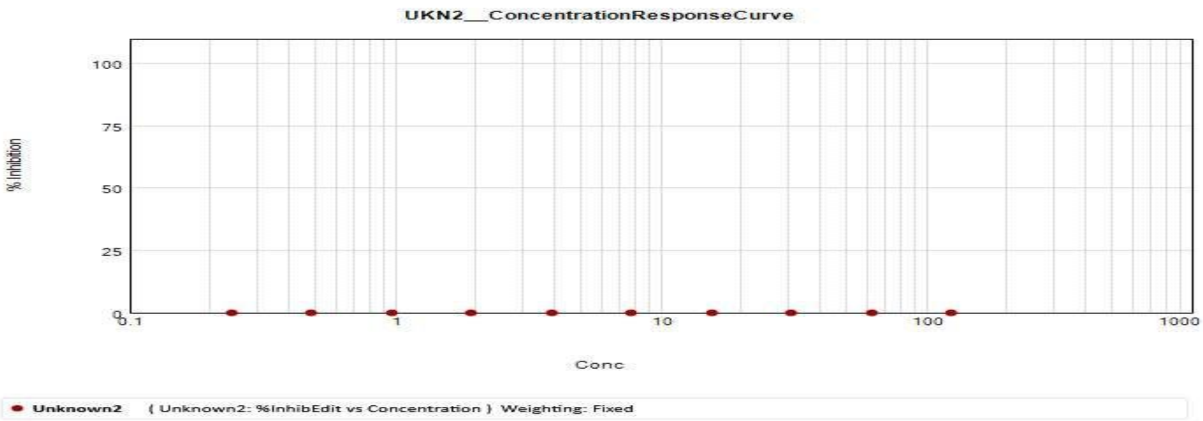

Curve Fit Results ▲

Curve Fit : 4-Parameter $y = D + \frac{A - D}{1 + \left(\frac{x}{C}\right)^B}$

	Parameter	Estimated Value	Std. Error	Confidence Interval
**Unknown2**	A	No fit		
R^2 =	B	No fit		
EC50 =	C	No fit		
	D	No fit		

Compound 8a

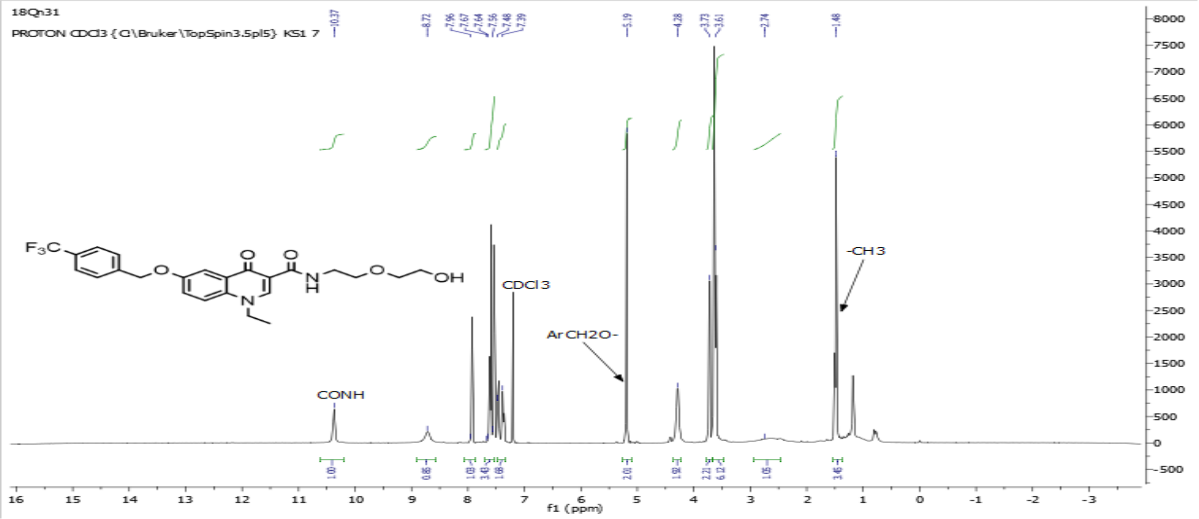

18Qn31
PROTON CDCl3 {C:\Bruker\TopSpin3.5pl5} KS1 7
CONH
CDCl3
ArCH2O-
-CH3
f1 (ppm)

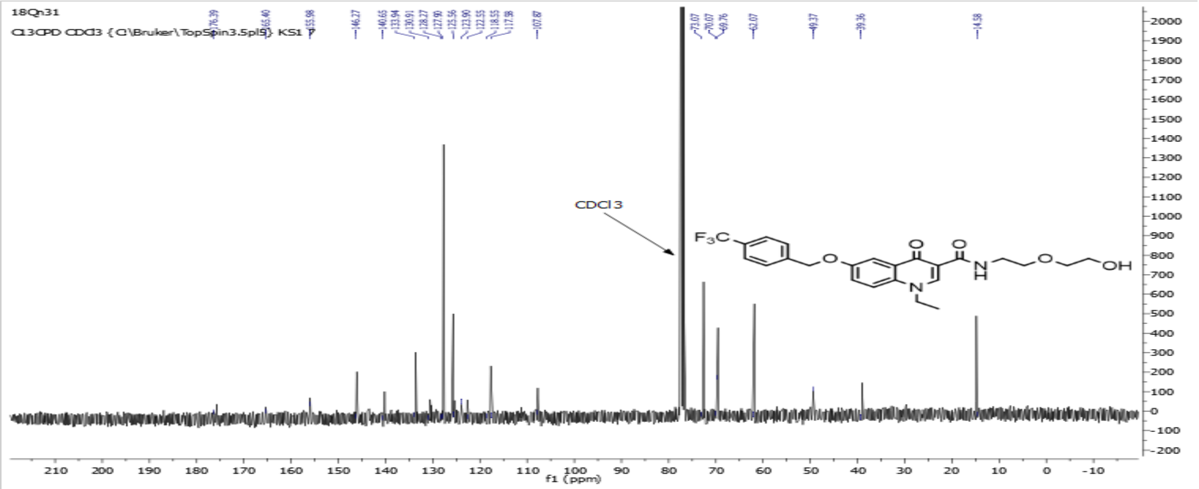

18Qn31
C13CPD CDCl3 {C:\Bruker\TopSpin3.5pl5} KS1 7
CDCl3
f1 (ppm)

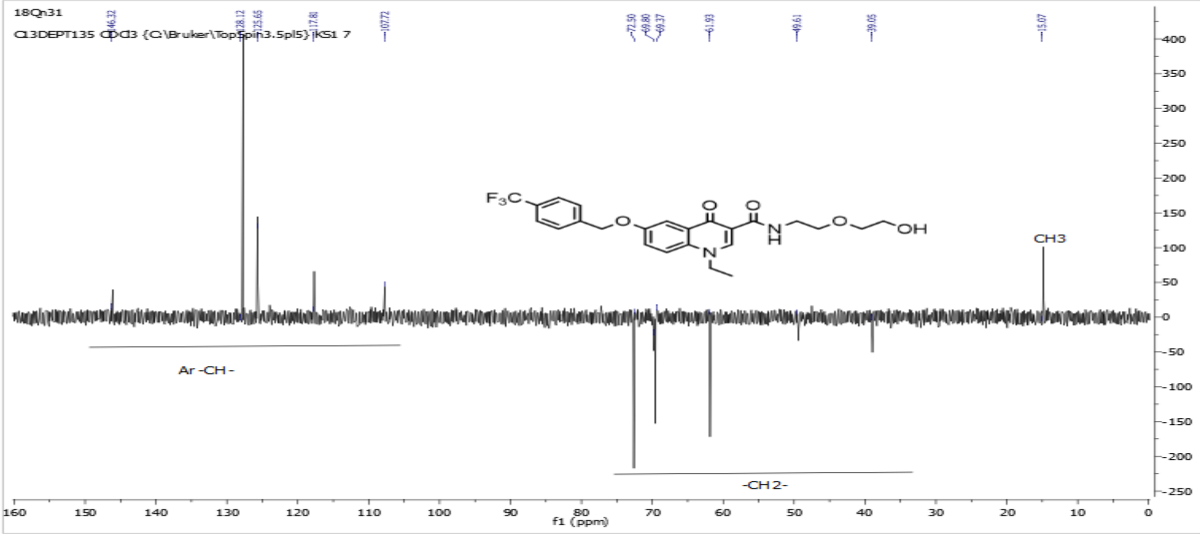

18Qn31
C13DEPT135 CDCl3 {C:\Bruker\TopSpin3.5pl5} KS1 7
CH3
Ar -CH -
-CH2-
f1 (ppm)

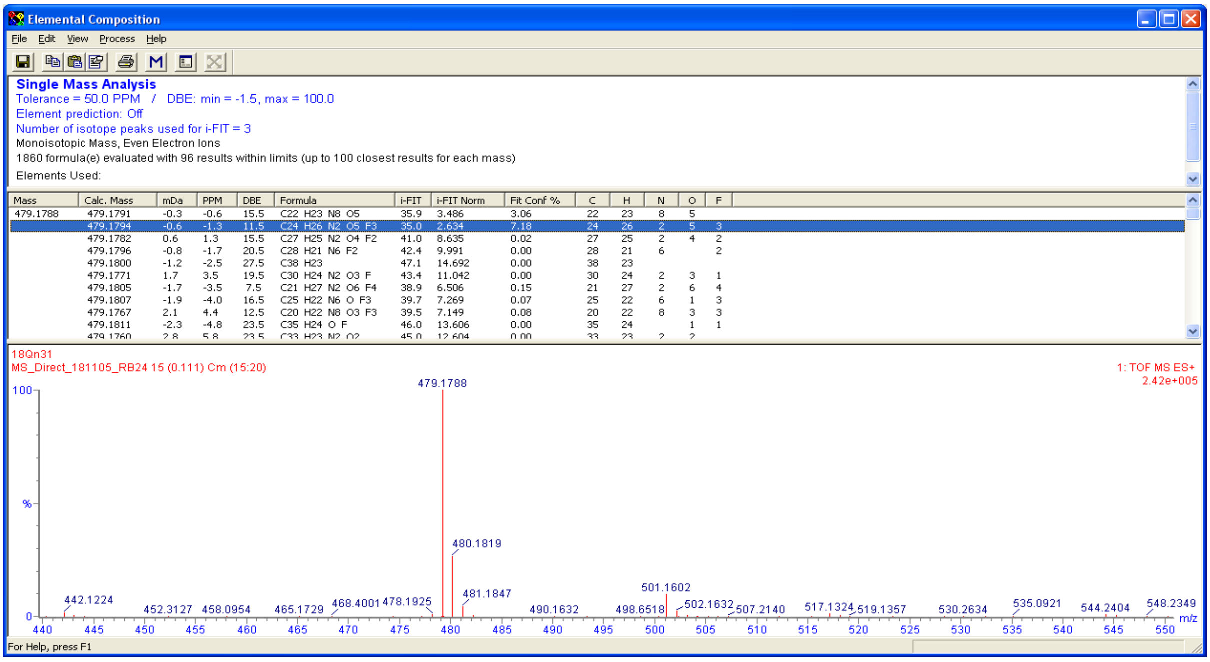

Dose-response against Mtb

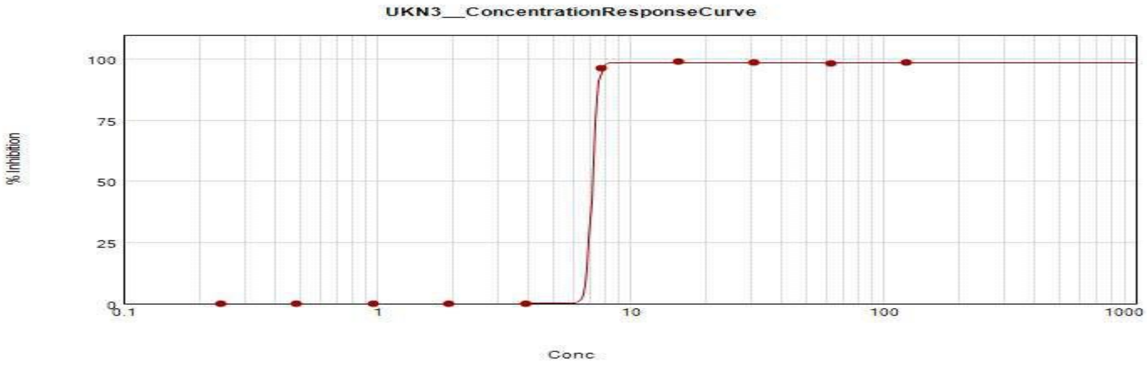

● **Unknown3** (Unknown3: %InhibEdit vs Concentration) Weighting: Fixed

Curve Fit Results ▲

Curve Fit : 4-Parameter $y = D + \frac{A - D}{1 + \left(\frac{x}{C}\right)^B}$

	Parameter	Estimated Value	Std. Error	Confidence Interval
**Unknown3** $R^2 = 1.000$ EC50 = 7.103	A	-2.35e-9	0.116	[-0.284, 0.284]
	B	38.21	3.28e+7	[-8.03e+7, 8.03e+7]
	C	7.103	5.81e+5	[-1.42e+6, 1.42e+6]
	D	98.64	0.115	[98.35, 98.92]

Compound 8b

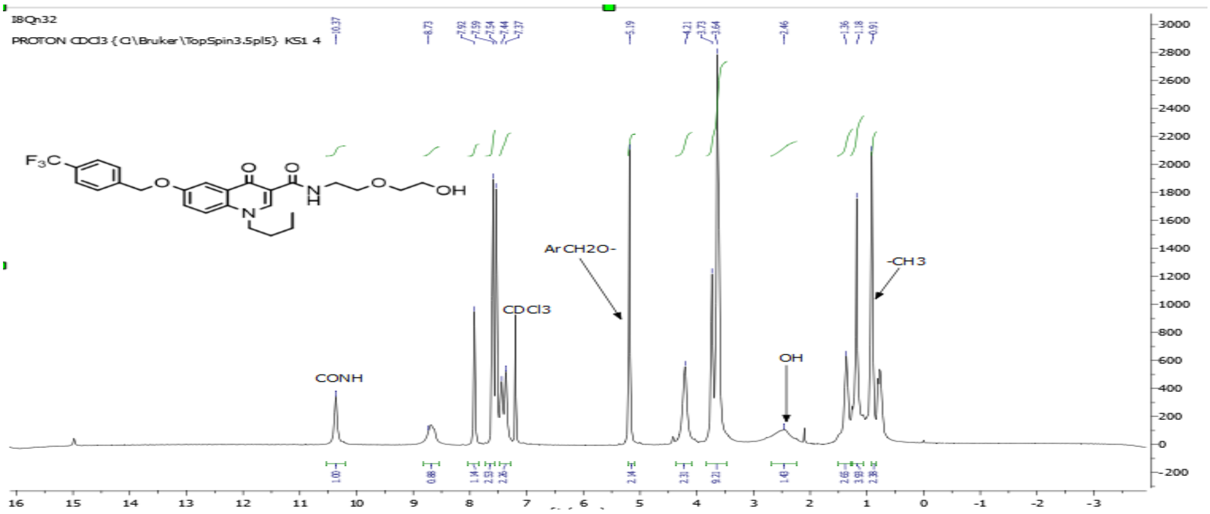
IBQn32
PROTON CDCl3 {C:\Bruker\TopSpin3.5pl5} KS1 4
CONH
ArCH2O-
CDCl3
OH
-CH3

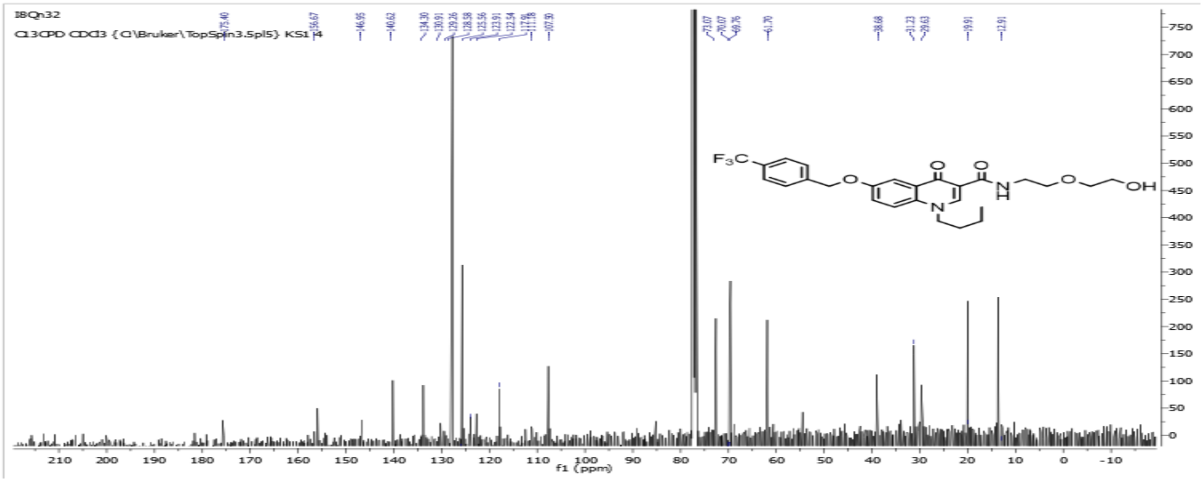
IBQn32
C13CPD CDCl3 {C:\Bruker\TopSpin3.5pl5} KS1 4
f1 (ppm)

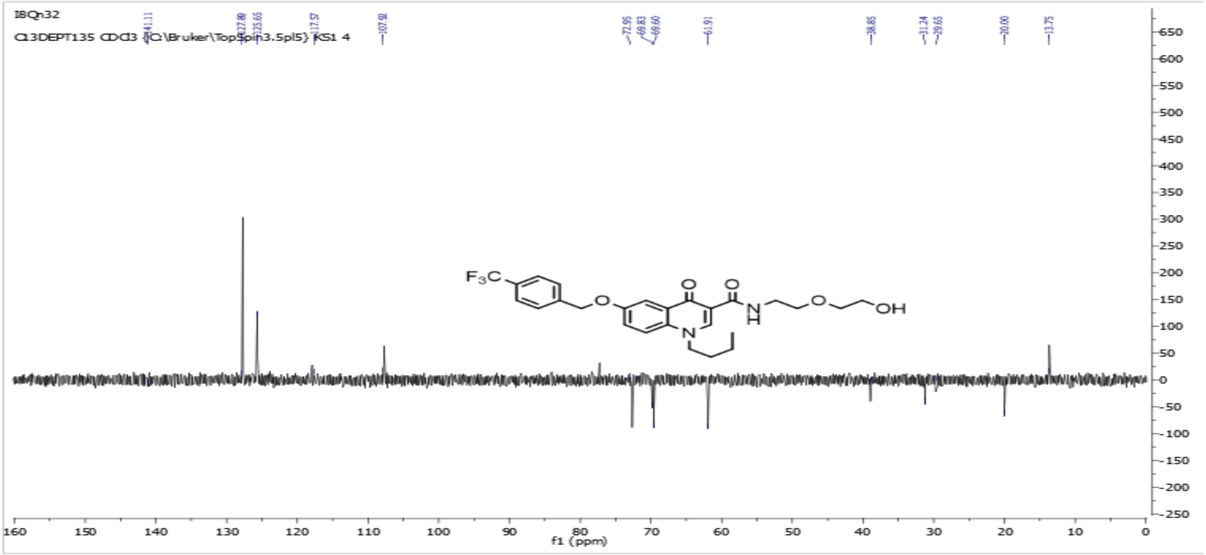
IBQn32
C13DEPT135 CDCl3 {C:\Bruker\TopSpin3.5pl5} KS1 4
f1 (ppm)

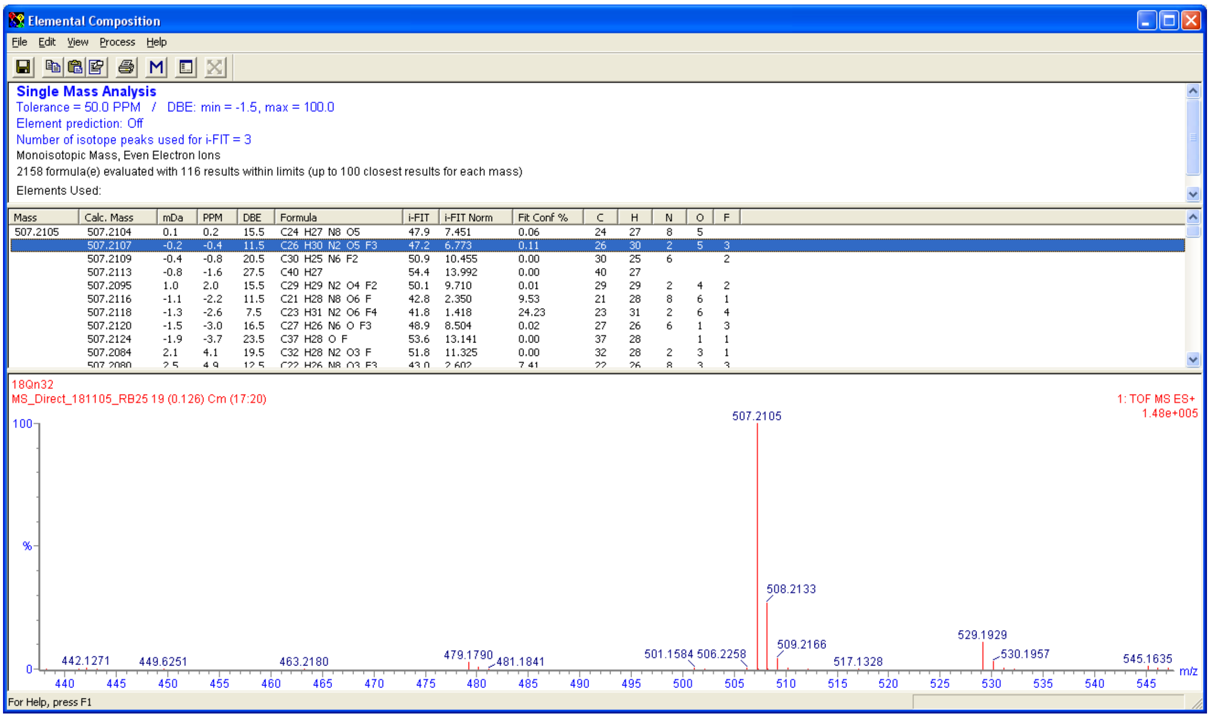

Dose-response against Mtb

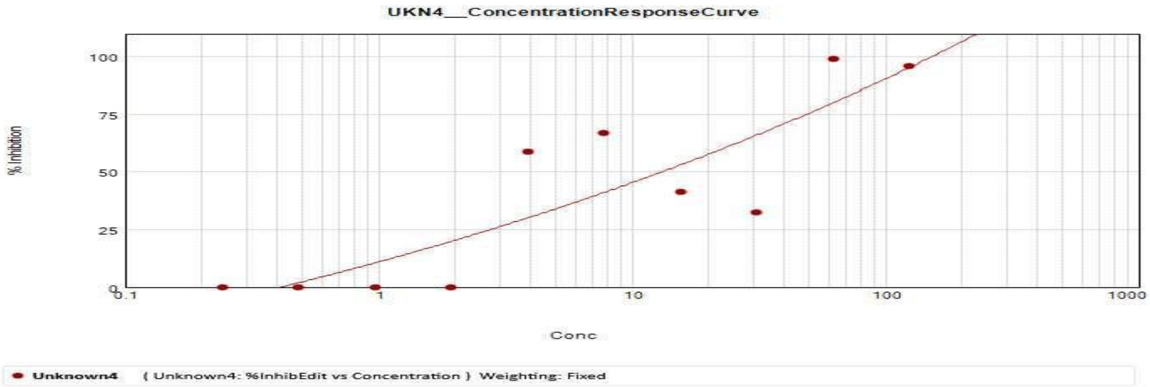

Curve Fit Results ▲

Curve Fit : 4-Parameter $y = D + \frac{A - D}{1 + \left(\frac{x}{C}\right)^B}$

	Parameter	Estimated Value	Std. Error	Confidence Interval
**Unknown4**<br>R^2 = 0.742<br>EC50 = 4.28e+36	A	-99.64	2517	[-6259, 6059]
	B	0.118	4.322	[-10.46, 10.69]
	C	4.28e+36	1.07e+43	[-2.61e+43, 2.61e+43]
	D	2.24e+6	6.55e+11	[-1.60e+12, 1.60e+12]

Compound 8c

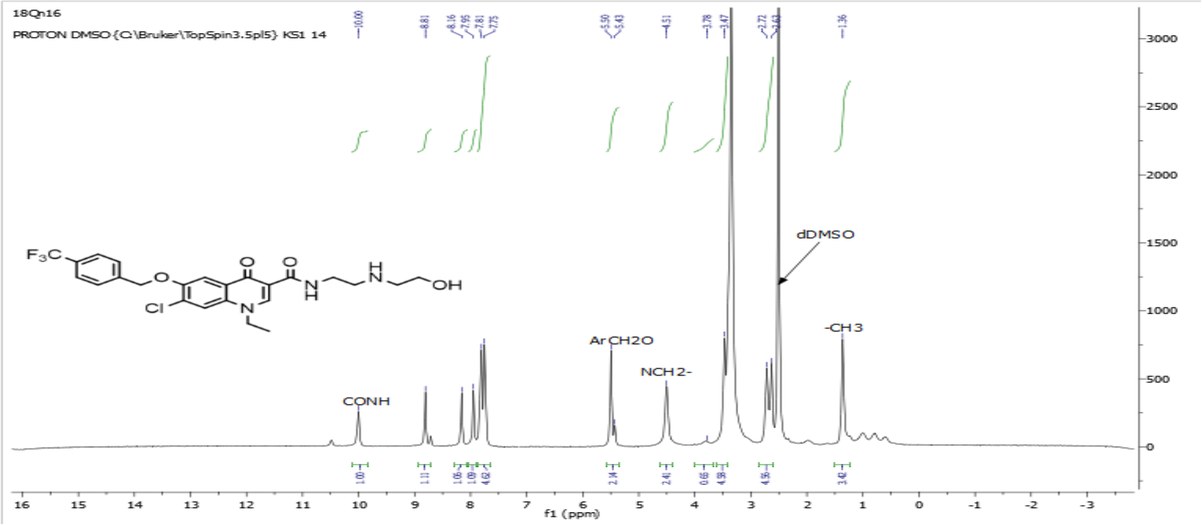
18Qn16
PROTON DMSO {C:\Bruker\TopSpin3.5pl5} KS1 14
dDMSO
-CH3
ArCH2O
NCH2-
CONH
f1 (ppm)

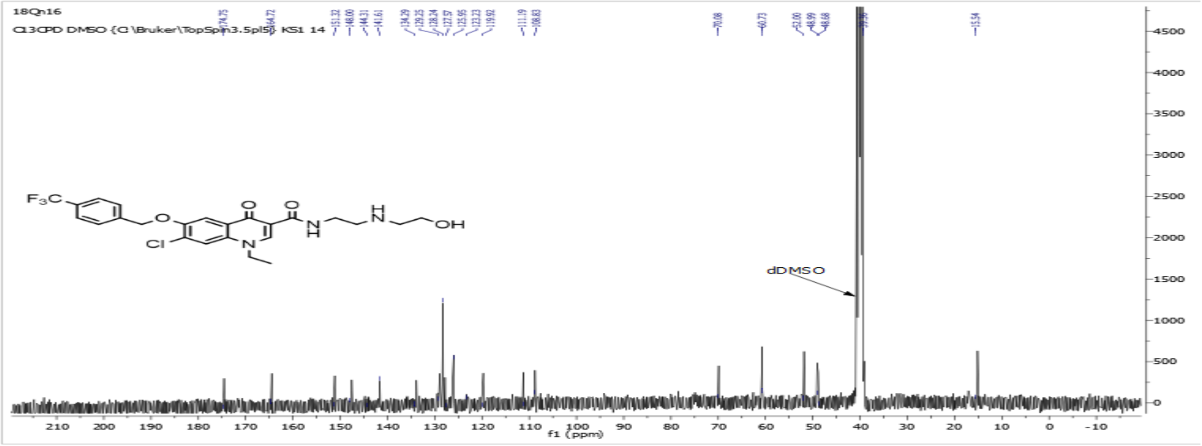
18Qn16
C13CPD DMSO {C:\Bruker\TopSpin3.5pl5} KS1 14
dDMSO
f1 (ppm)

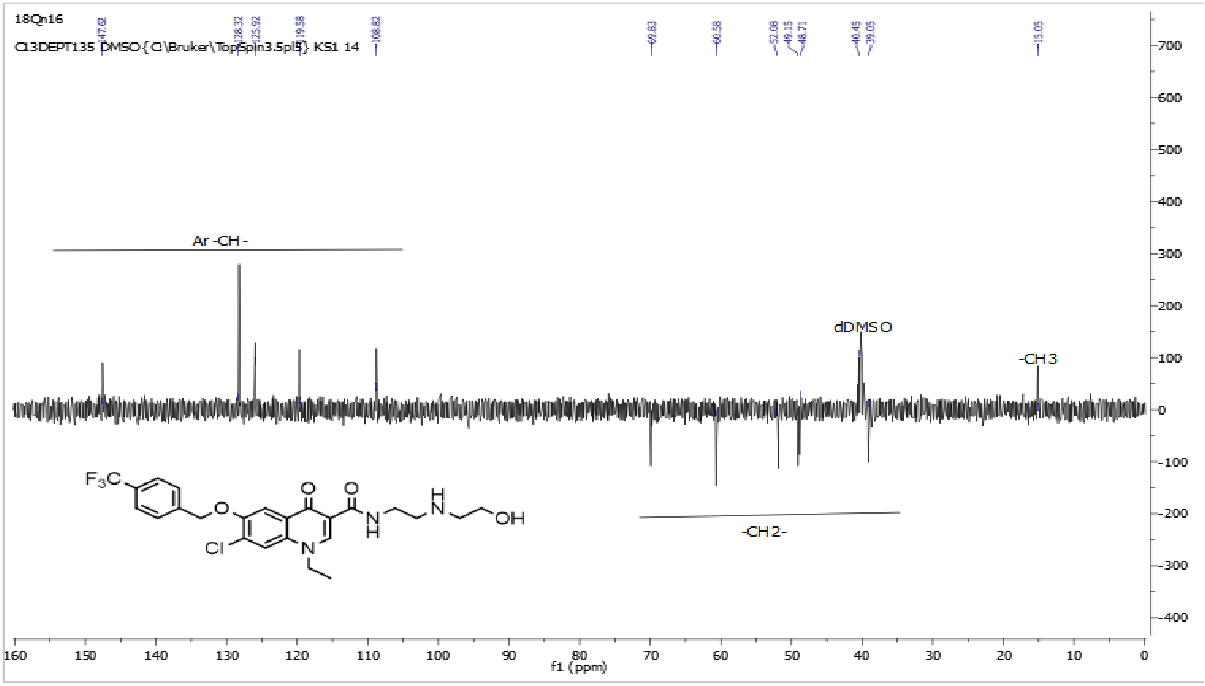
18Qn16
C13DEPT135 DMSO {C:\Bruker\TopSpin3.5pl5} KS1 14
Ar -CH -
dDMSO
-CH3
-CH2-
f1 (ppm)

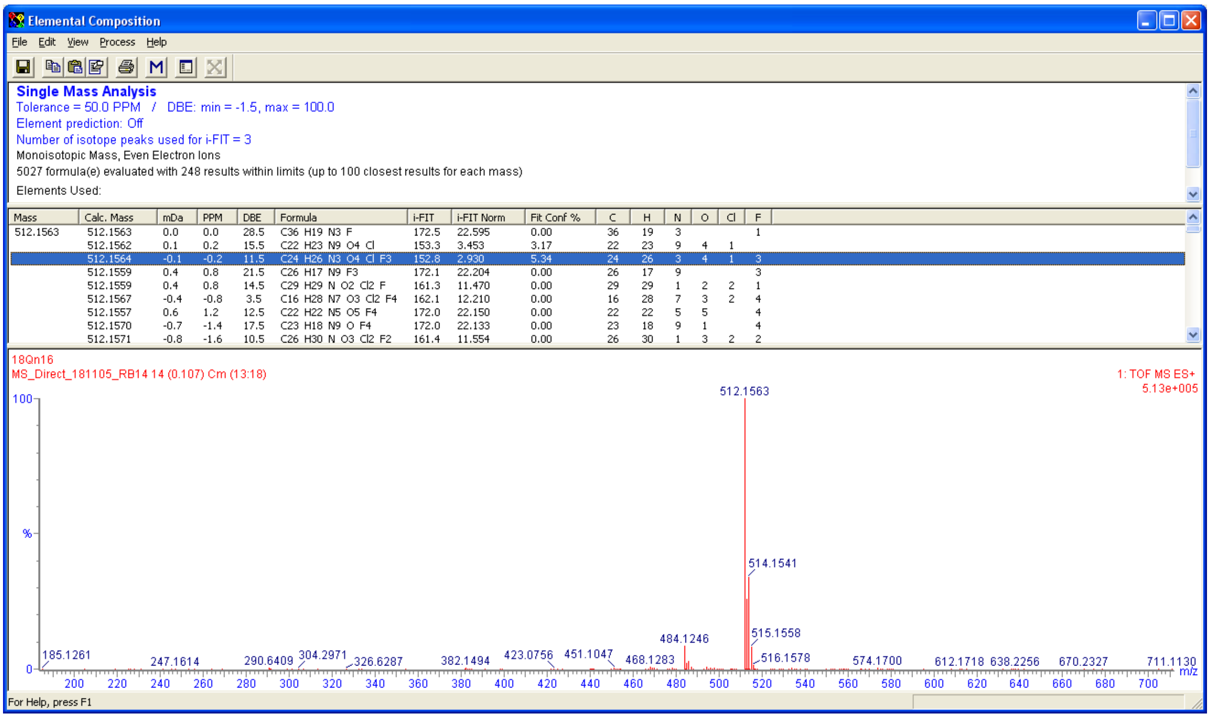

Dose-response against Mtb

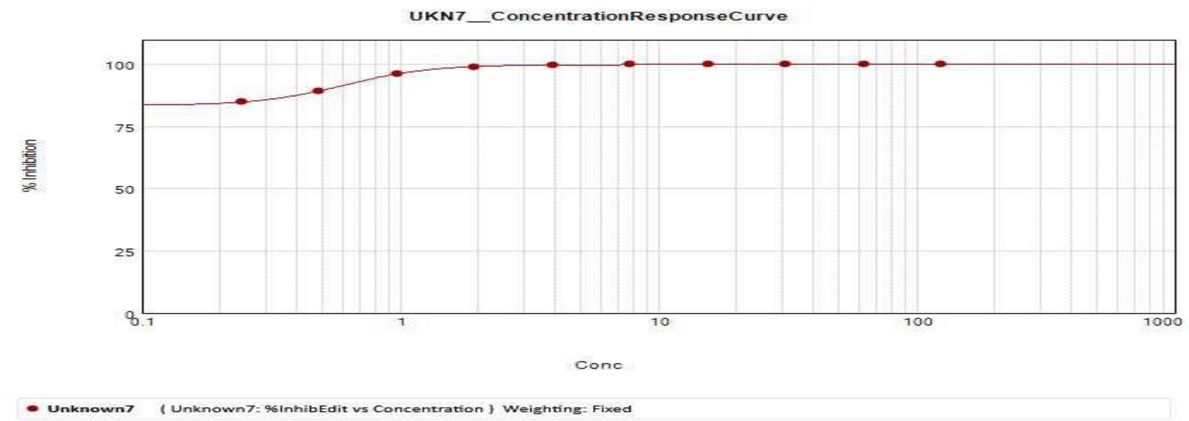

Curve Fit Results ▲

Curve Fit : 4-Parameter $y = D + \frac{A - D}{1 + \left(\frac{x}{C}\right)^{B}}$

	Parameter	Estimated Value	Std. Error	Confidence Interval
Unknown7 R^2 = 0.999 EC50 = 0.609	A	83.57	0.318	[82.79, 84.34]
	B	2.610	0.116	[2.325, 2.895]
	C	0.609	0.014	[0.573, 0.644]
	D	99.96	0.063	[99.80, 100.1]

Compound 8d

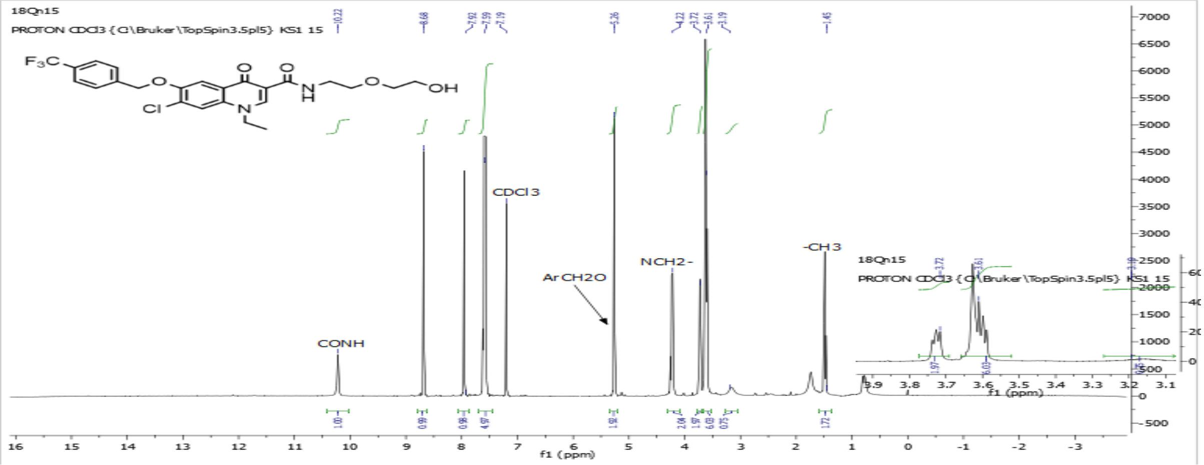
18Qn15
PROTON CDCl3 {C:\Bruker\TopSpin3.5pl5} KS1 15
CONH
Ar CH2O
NCH2-
CDCl3
-CH3
f1 (ppm)

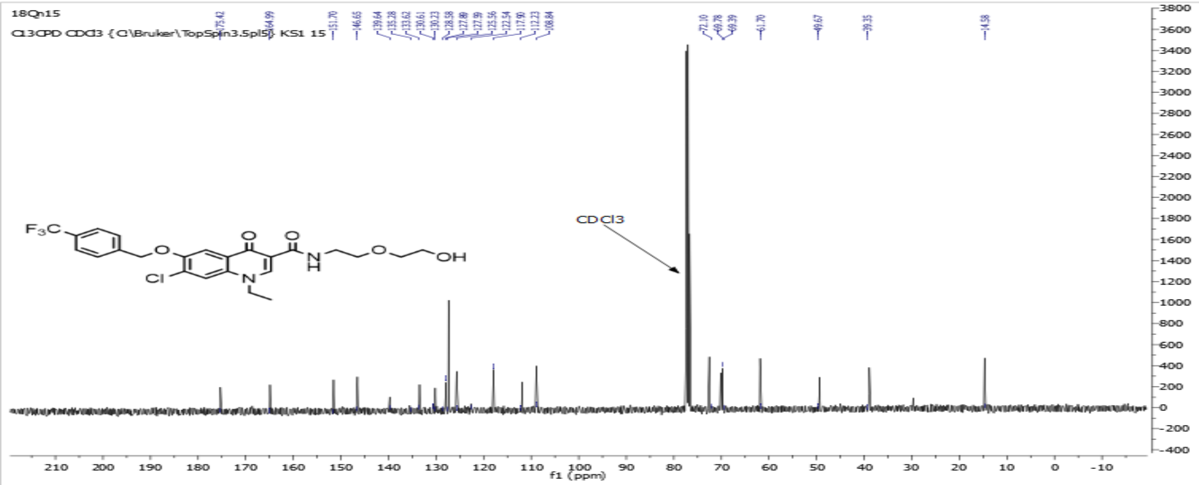
18Qn15
C13CPD CDCl3 {C:\Bruker\TopSpin3.5pl5} KS1 15
CDCl3
f1 (ppm)

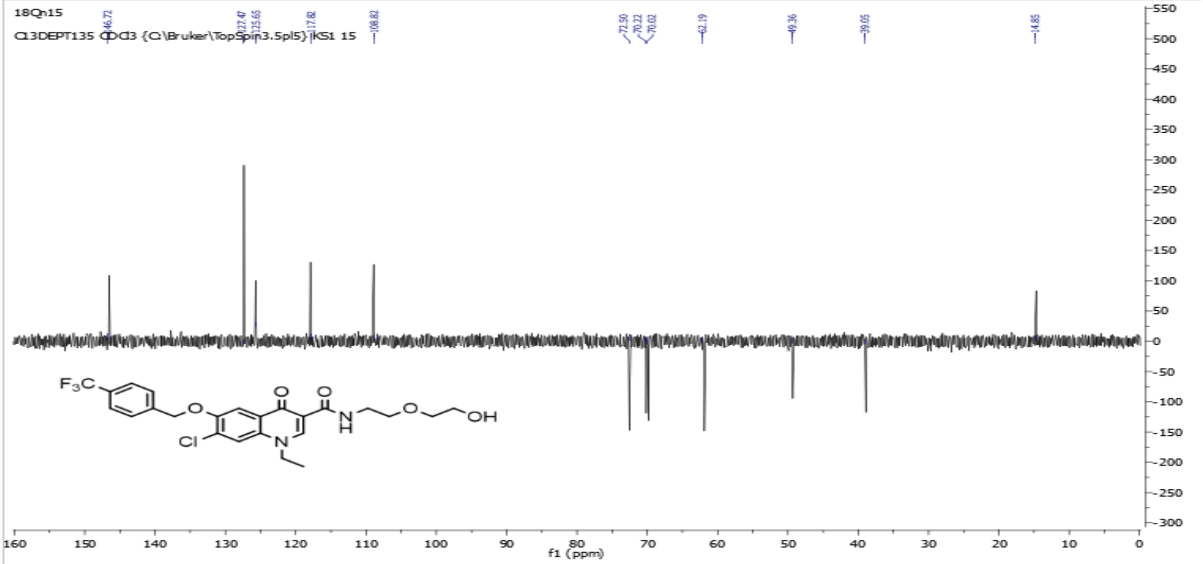
18Qn15
C13DEPT135 CDCl3 {C:\Bruker\TopSpin3.5pl5} KS1 15
f1 (ppm)

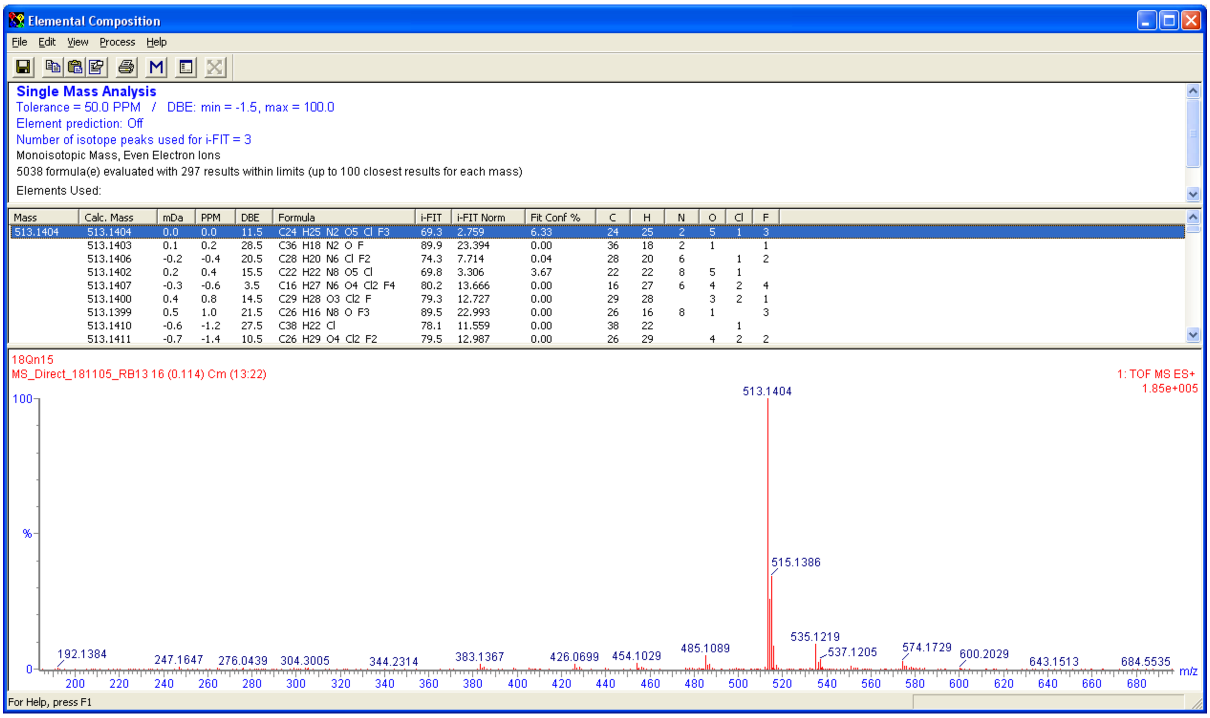

Dose-response against Mtb

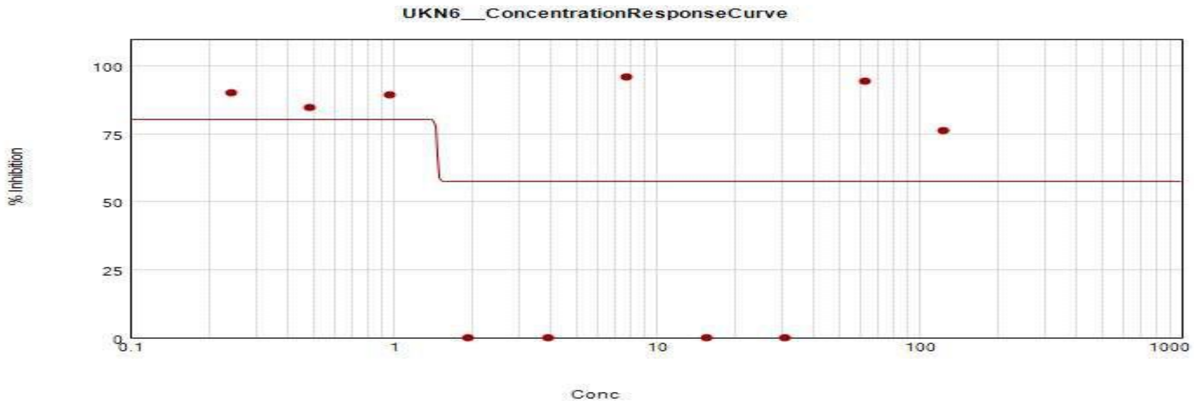

● Unknown6 (Unknown6: %InhibEdit vs Concentration) Weighting: Fixed

Curve Fit Results ▲

Curve Fit : 4-Parameter $y = D + \frac{A - D}{1 + \left(\frac{x}{C}\right)^{B}}$

	Parameter	Estimated Value	Std. Error	Confidence Interval
Unknown6<br>$R^2 = 0.124$<br>EC50 = 1.468	A	80.41	37.24	[-10.72, 171.5]
	B	135.3	3.85e+24	[-9.41e+24, 9.41e+24]
	C	1.468	1.19e+22	[-2.91e+22, 2.91e+22]
	D	57.69	21.51	[5.073, 110.3]

Compound 8e

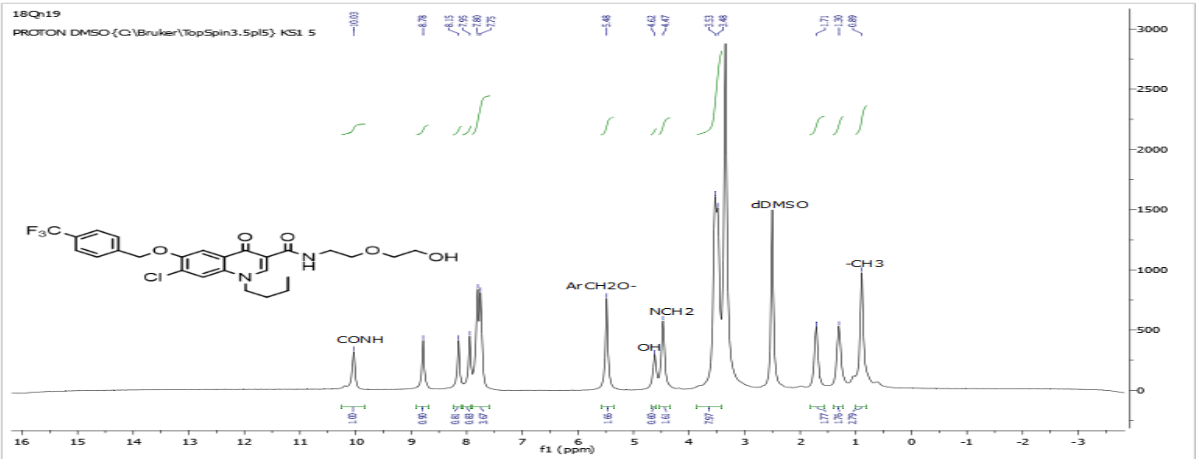
18Qn19
PROTON DMSO {C:\Bruker\TopSpin3.5pl5} KS1 5
CONH
ArCH2O-
OH
NCH2
dDMSO
-CH3
f1 (ppm)

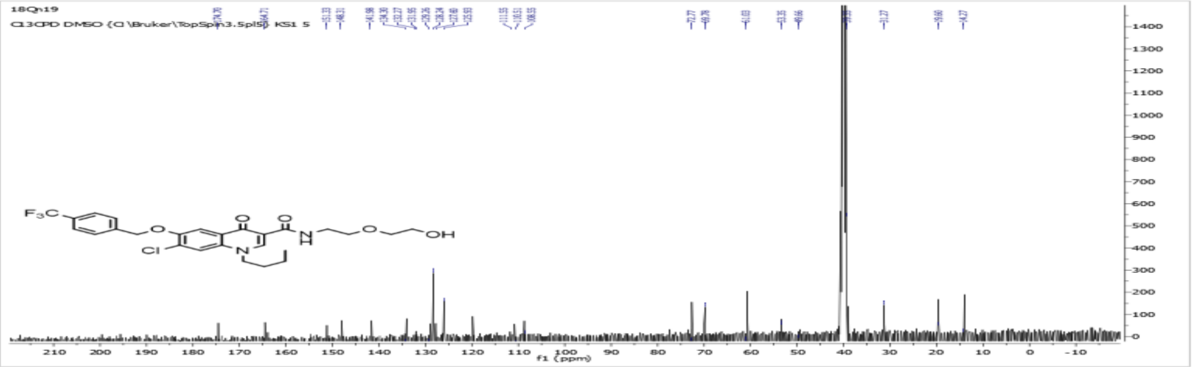
18Qn19
C13CPD DMSO {C:\Bruker\TopSpin3.5pl5} KS1 5
f1 (ppm)

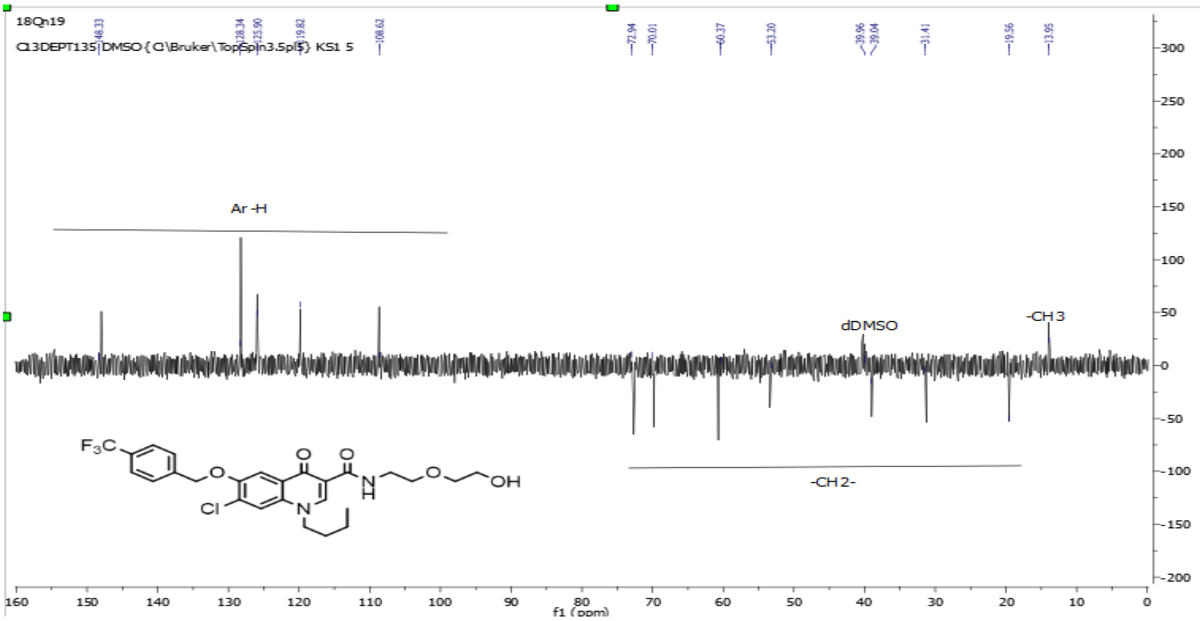
18Qn19
C13DEPT135 DMSO {C:\Bruker\TopSpin3.5pl5} KS1 5
Ar-H
dDMSO
-CH3
-CH2-
f1 (ppm)

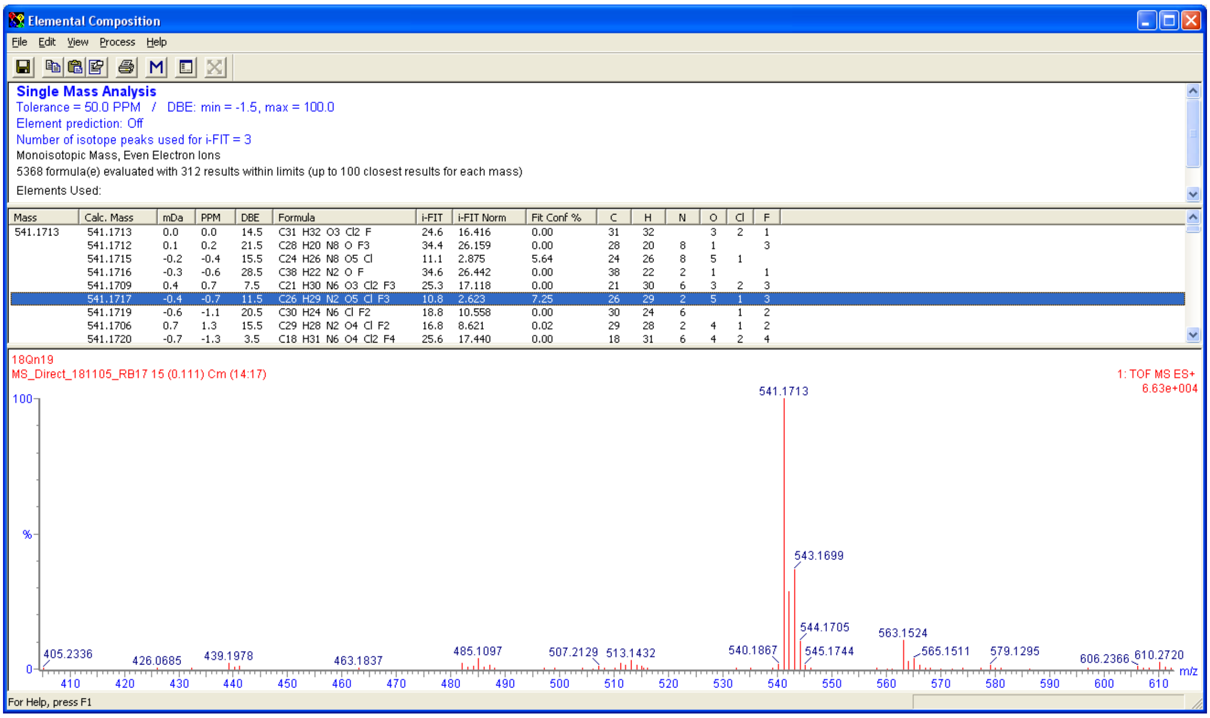

Dose-response against Mtb

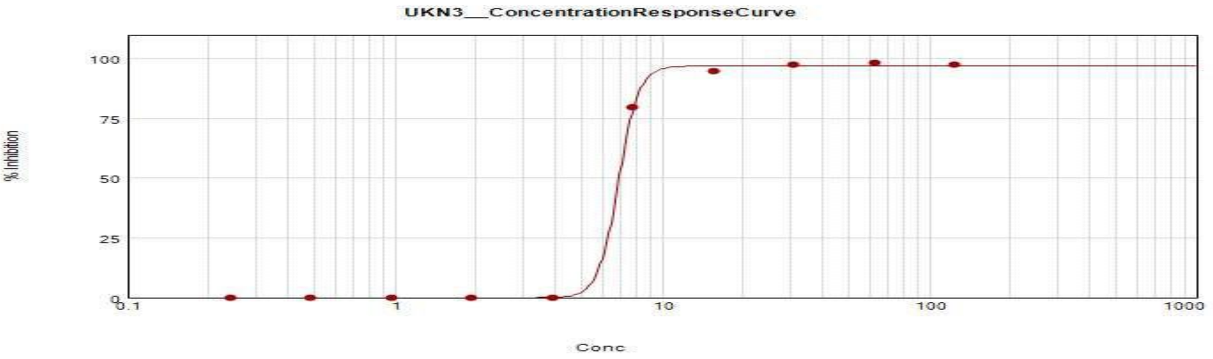

● Unknown3 (Unknown3: %InhibEdit vs Concentration) Weighting: Fixed

Curve Fit Results ▲

Curve Fit : 4-Parameter $y = D + \frac{A - D}{1 + \left(\frac{x}{C}\right)^B}$

	Parameter	Estimated Value	Std. Error	Confidence Interval
Unknown3<br>R^2 = 1.000<br>EC50 = 6.851	A	-0.028	0.583	[-1.455, 1.398]
	B	11.60	13.16	[-20.60, 43.81]
	C	6.851	1.023	[4.347, 9.354]
	D	96.96	0.583	[95.53, 98.39]

Compound 8f

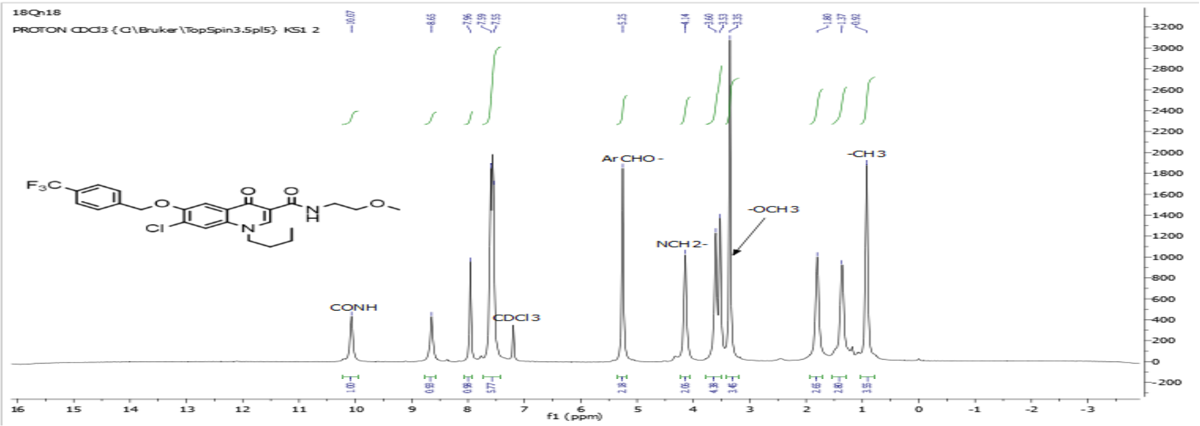
18Qn18
PROTON CDCl3 {C:\Bruker\TopSpin3.5pl5} KS1 2
CONH
CDCl3
Ar CHO-
NCH2-
-OCH3
-CH3
f1 (ppm)

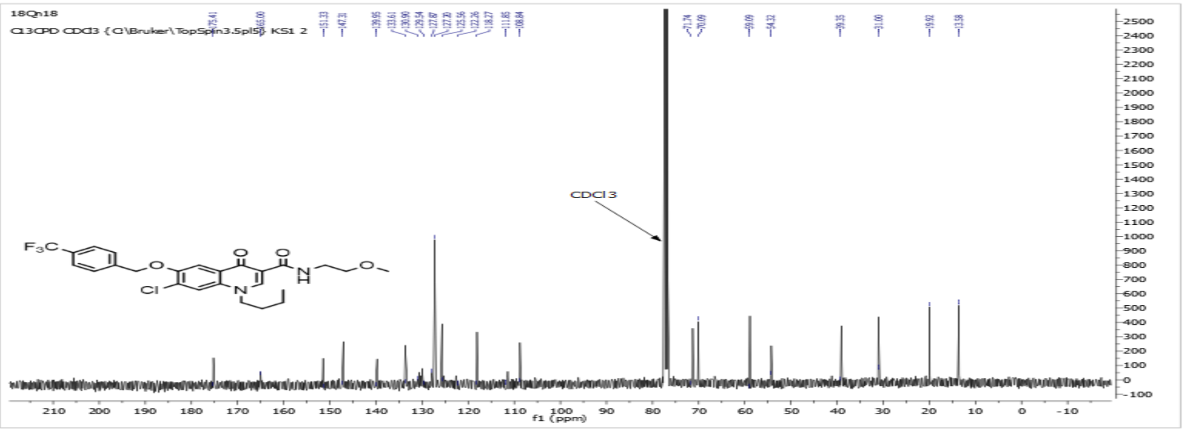
18Qn18
C13CPD CDCl3 {C:\Bruker\TopSpin3.5pl5} KS1 2
CDCl3
f1 (ppm)

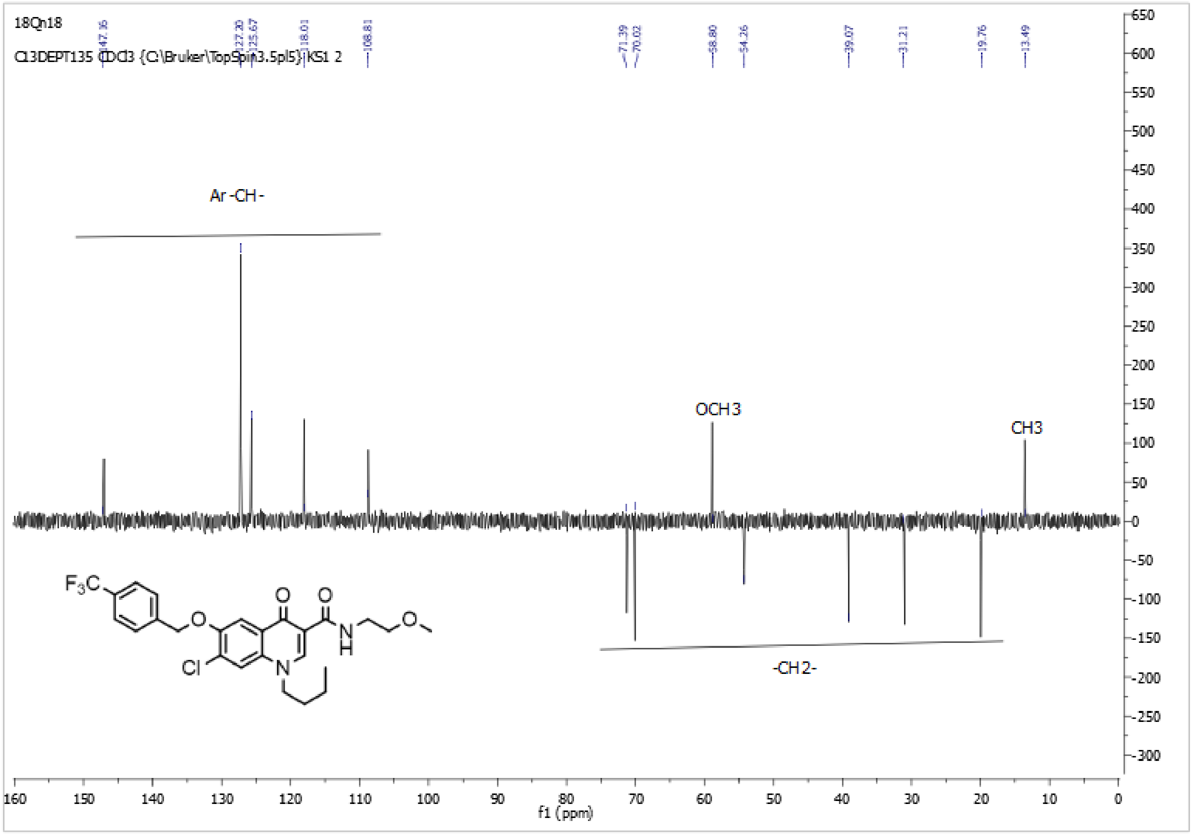
18Qn18
C13DEPT135 CDCl3 {C:\Bruker\TopSpin3.5pl5} KS1 2
147.35
127.20
125.67
118.01
108.81
71.39
70.02
58.80
54.26
39.07
31.21
19.76
13.49
Ar-CH-
OCH3
CH3
-CH2-
f1 (ppm)

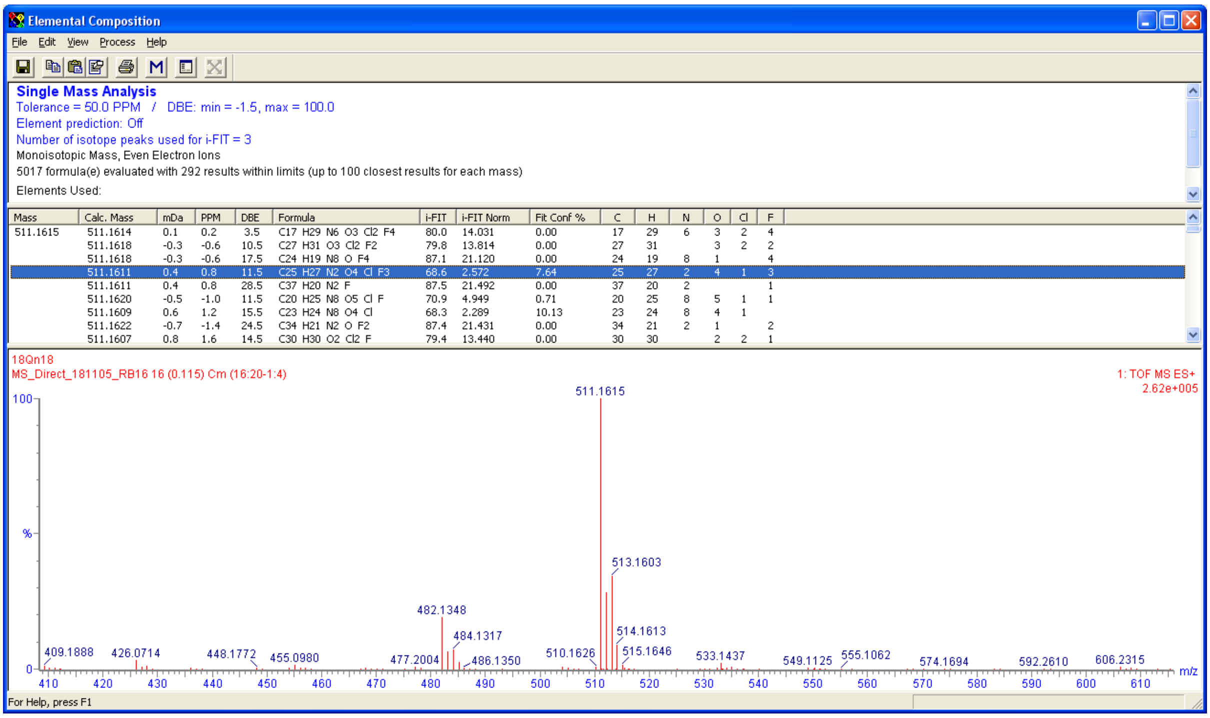

Dose-response against Mtb

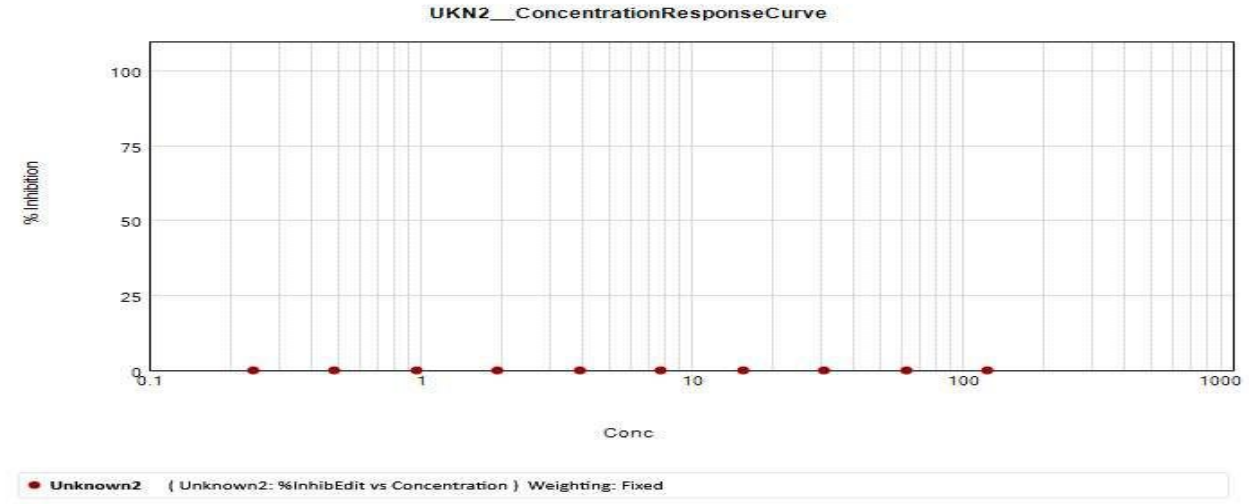

Curve Fit : 4-Parameter $y = D + \frac{A - D}{1 + \left(\frac{x}{C}\right)^B}$

	Parameter	Estimated Value	Std. Error	Confidence Interval
Unknown2	A	No fit		
R^2 =	B	No fit		
EC50 =	C	No fit		
	D	No fit		

Compound 8g

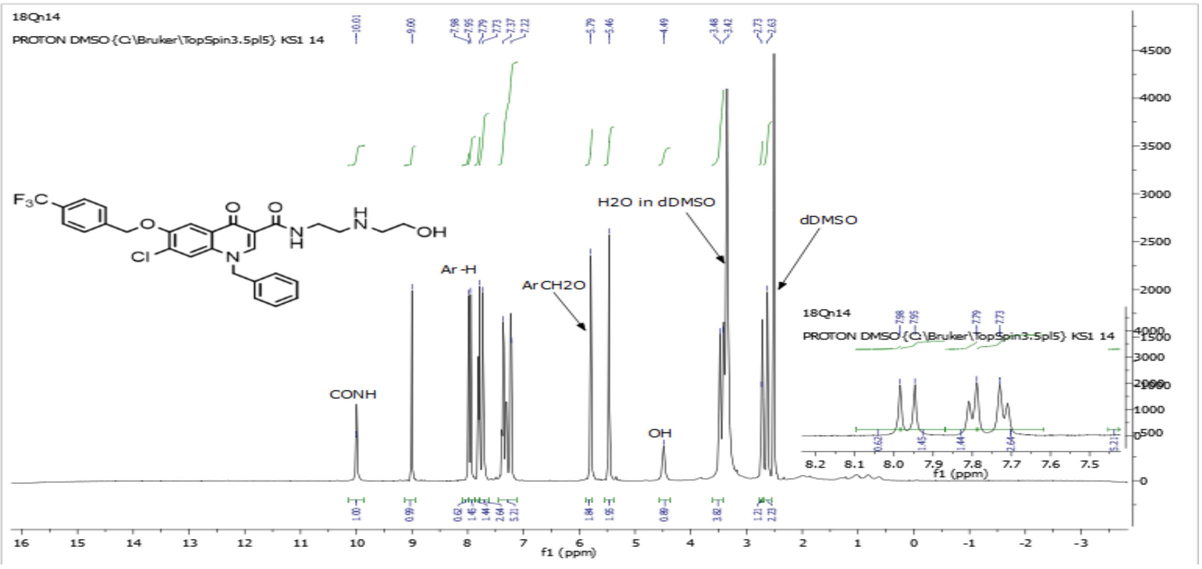
18Qn14
PROTON DMSO {C:\Bruker\TopSpin3.5pl5} KS1 14
CONH
Ar-H
ArCH2O
H2O in dDMSO
dDMSO
OH
f1 (ppm)

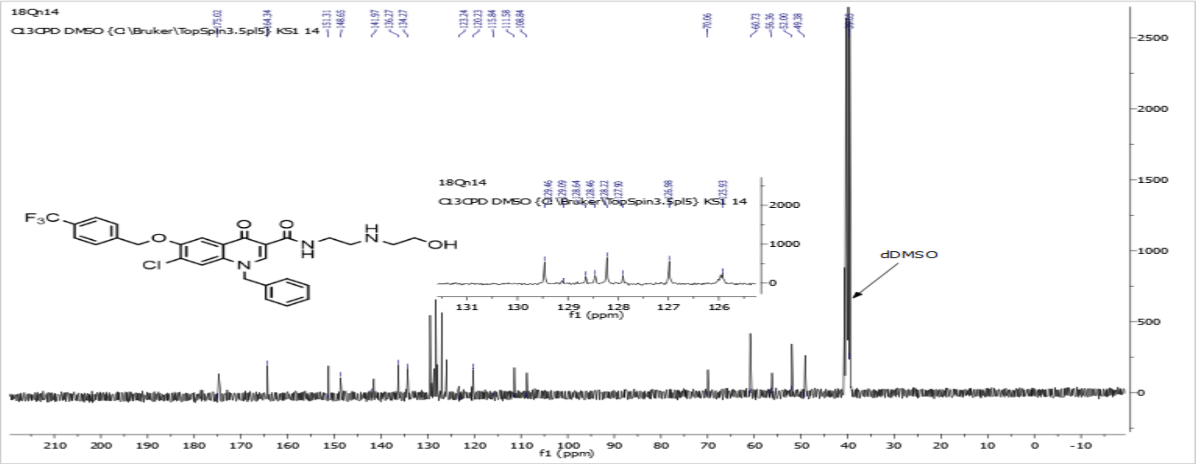
18Qn14
C13CPD DMSO {C:\Bruker\TopSpin3.5pl5} KS1 14
dDMSO
f1 (ppm)

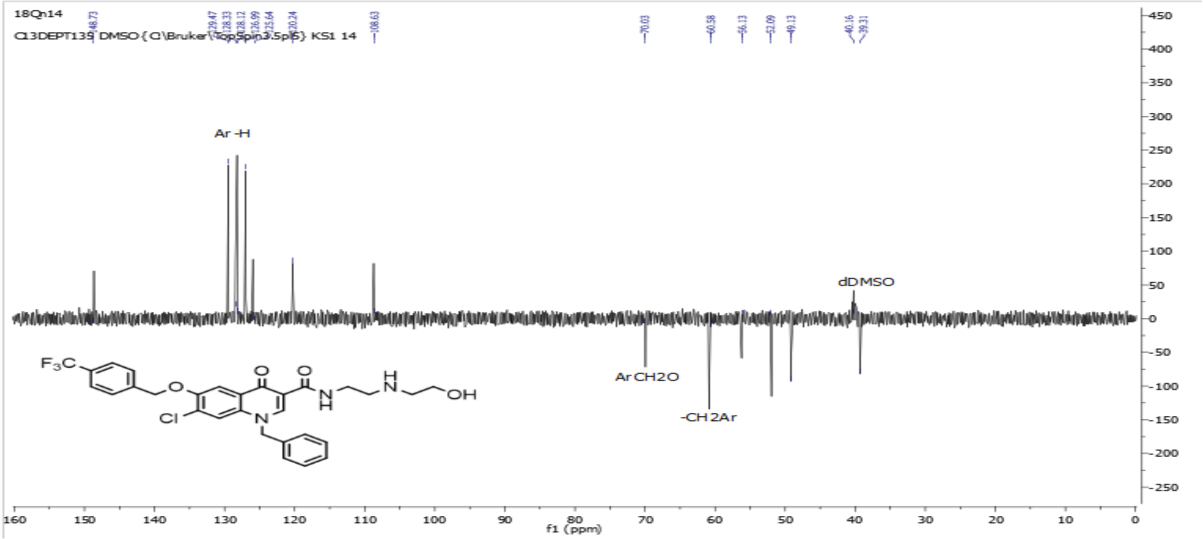
18Qn14
C13DEPT135 DMSO {C:\Bruker\TopSpin3.5pl5} KS1 14
Ar-H
dDMSO
ArCH2O
-CH2Ar
f1 (ppm)

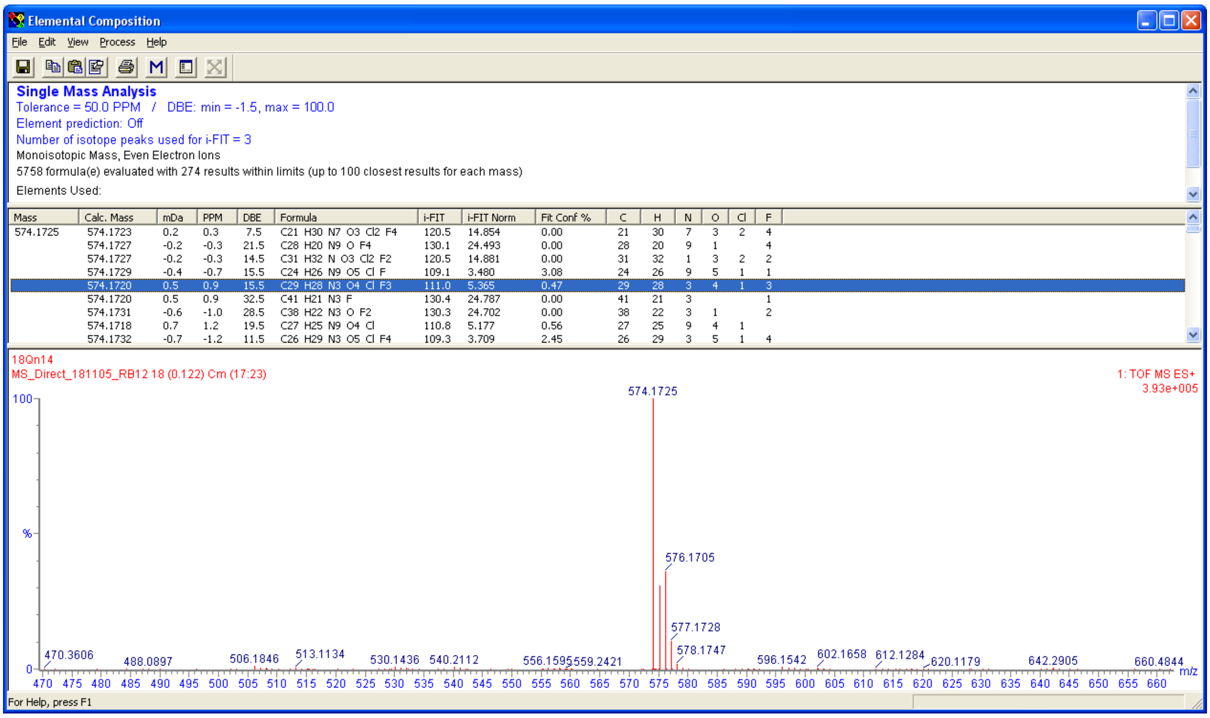

Dose-response against Mtb

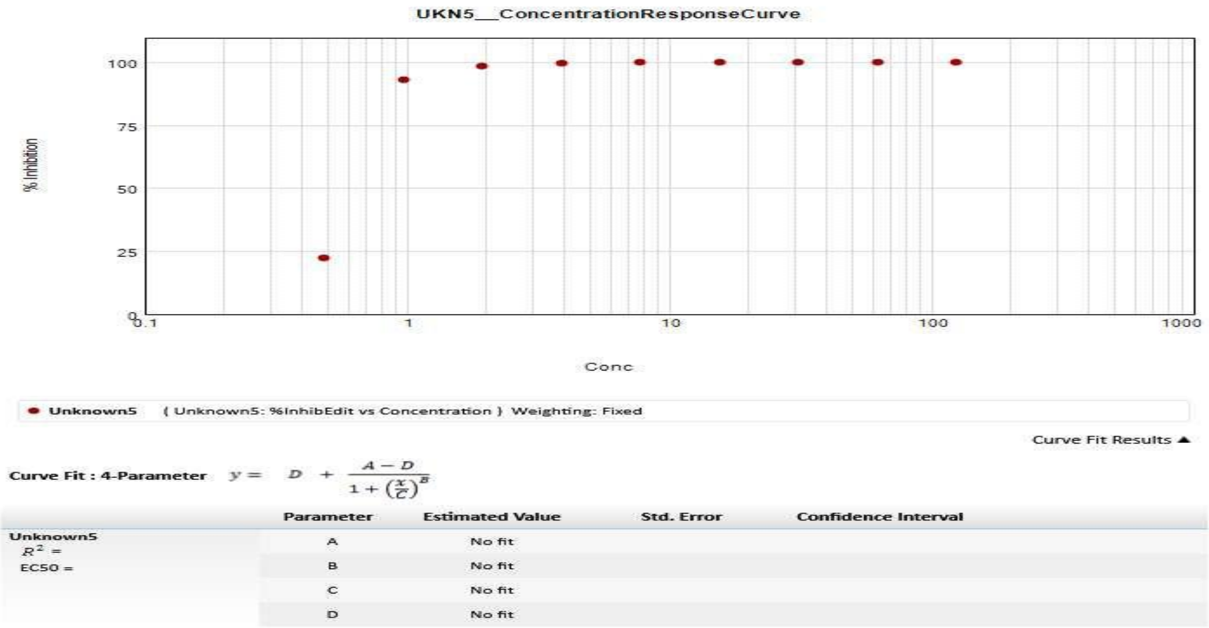

Compound 8h

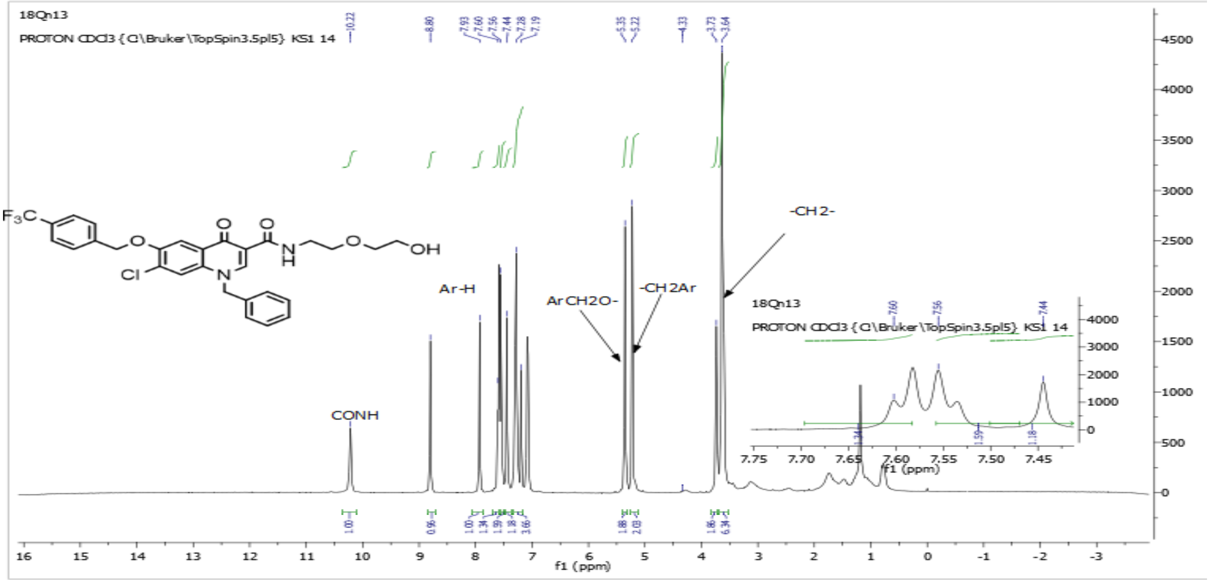

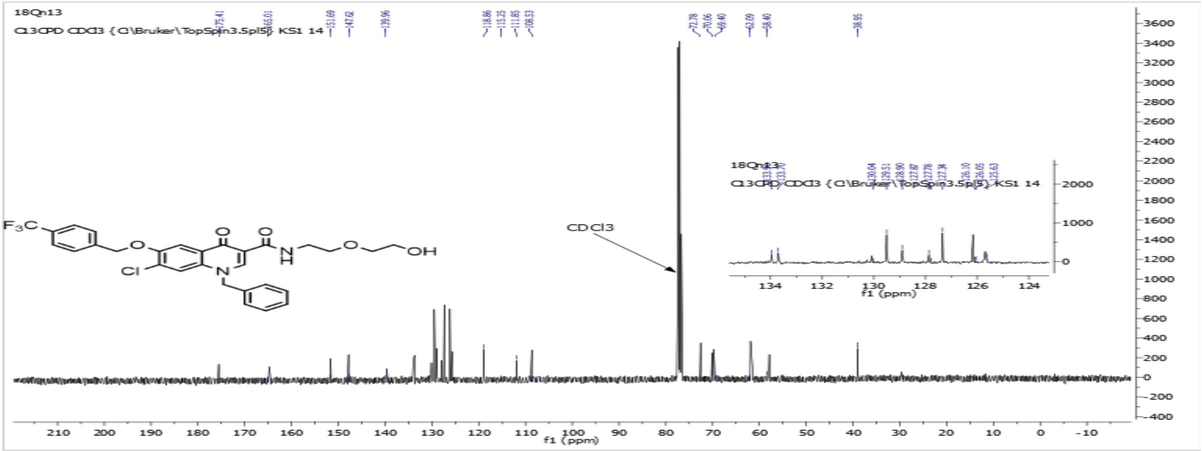

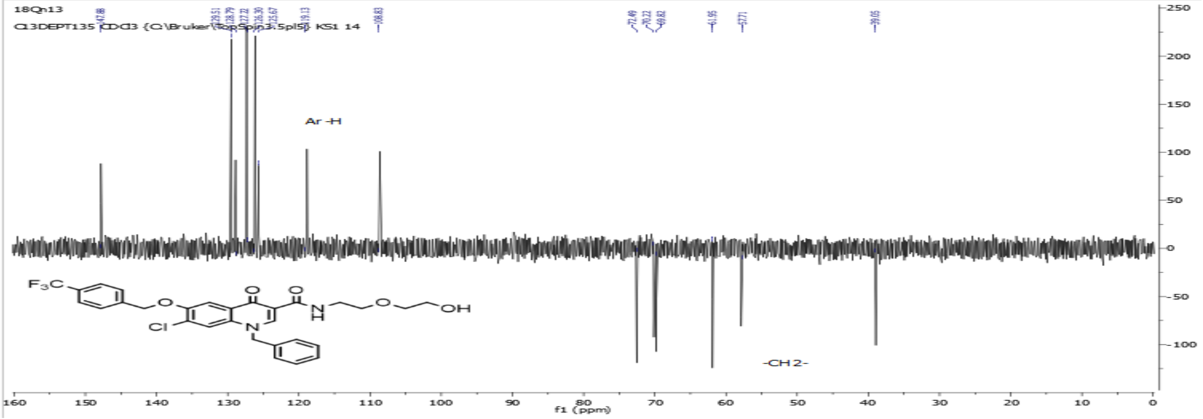

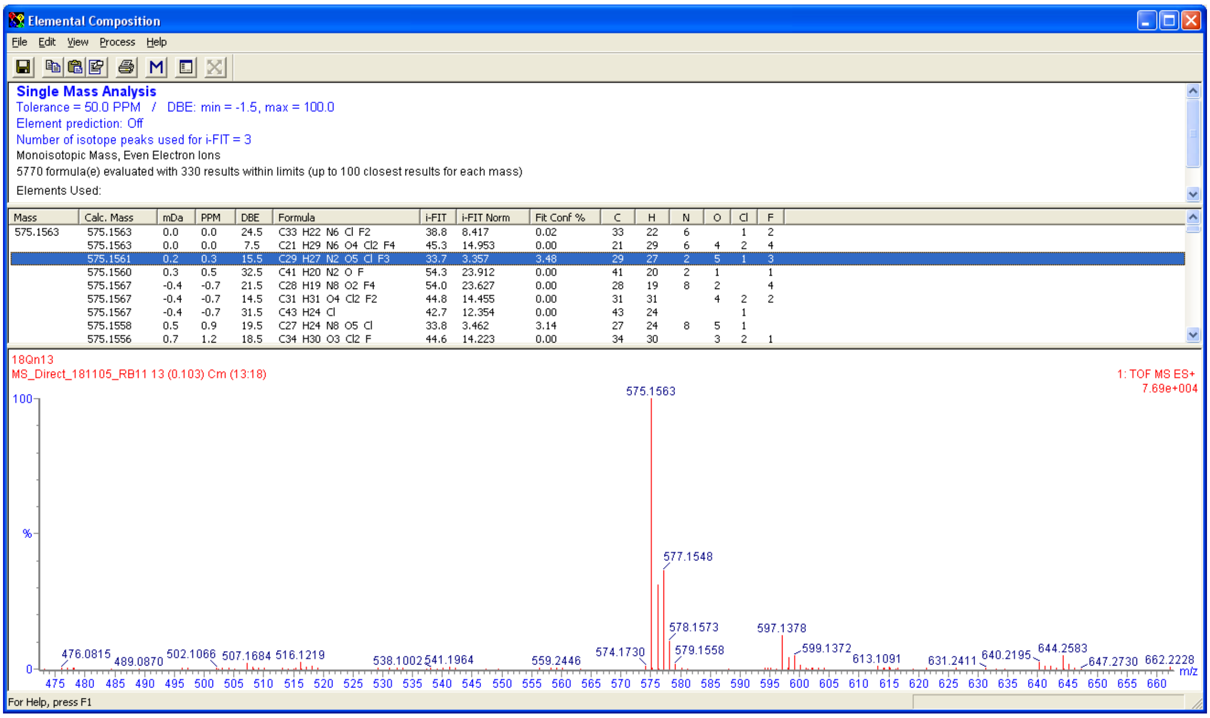

Mass	Calc. Mass	mDa	PPM	DBE	Formula	i-FIT	i-FIT Norm	Fit Conf %	C	H	N	O	Cl	F
575.1563	575.1563	0.0	0.0	24.5	C33 H22 N6 Cl F2	38.8	8.417	0.02	33	22	6		1	2
	575.1563	0.0	0.0	7.5	C21 H29 N6 O4 Cl2 F4	45.3	14.953	0.00	21	29	6	4	2	4
	575.1561	0.2	0.3	15.5	C29 H27 N2 O5 Cl F3	33.7	3.357	3.48	29	27	2	5	1	3
	575.1560	0.3	0.5	32.5	C41 H20 N2 O F	54.3	23.912	0.00	41	20	2	1		1
	575.1567	-0.4	-0.7	21.5	C28 H19 N8 O2 F4	54.0	23.627	0.00	28	19	8	2		4
	575.1567	-0.4	-0.7	14.5	C31 H31 O4 Cl2 F2	44.8	14.455	0.00	31	31		4	2	2
	575.1567	-0.4	-0.7	31.5	C43 H24 Cl	42.7	12.354	0.00	43	24			1	
	575.1558	0.5	0.9	19.5	C27 H24 N8 O5 Cl	33.8	3.462	3.14	27	24	8	5	1	
	575.1556	0.7	1.2	18.5	C34 H30 O3 Cl2 F	44.6	14.223	0.00	34	30		3	2	1

Dose-response against Mtb

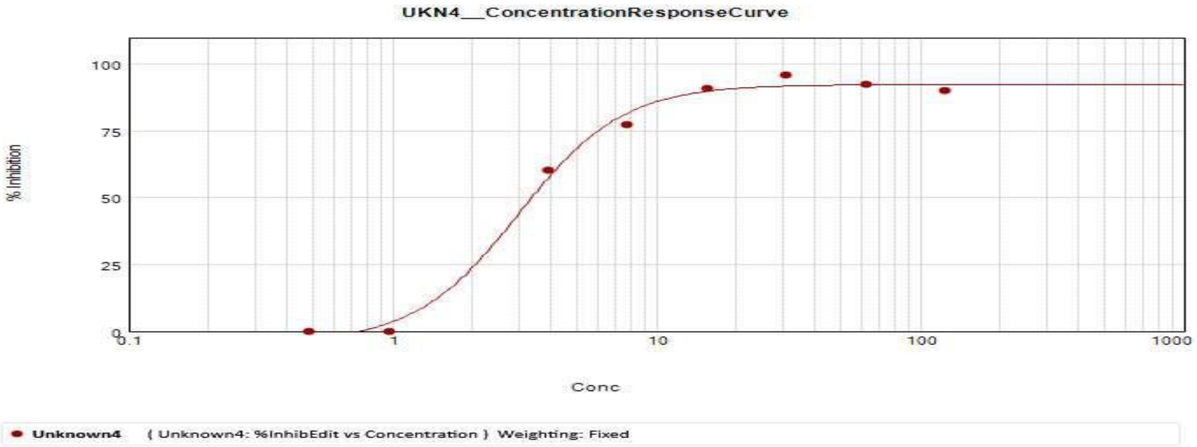

Curve Fit Results ▲

Curve Fit : 4-Parameter $y = D + \frac{A - D}{1 + \left(\frac{x}{C}\right)^B}$

	Parameter	Estimated Value	Std. Error	Confidence Interval
Unknown4<br>$R^2 = 0.995$<br>EC50 = 3.004	A	-4.305	5.073	[-18.39, 9.779]
	B	2.200	0.529	[0.731, 3.670]
	C	3.004	0.384	[1.937, 4.071]
	D	92.42	2.241	[86.19, 98.64]

Compound 11

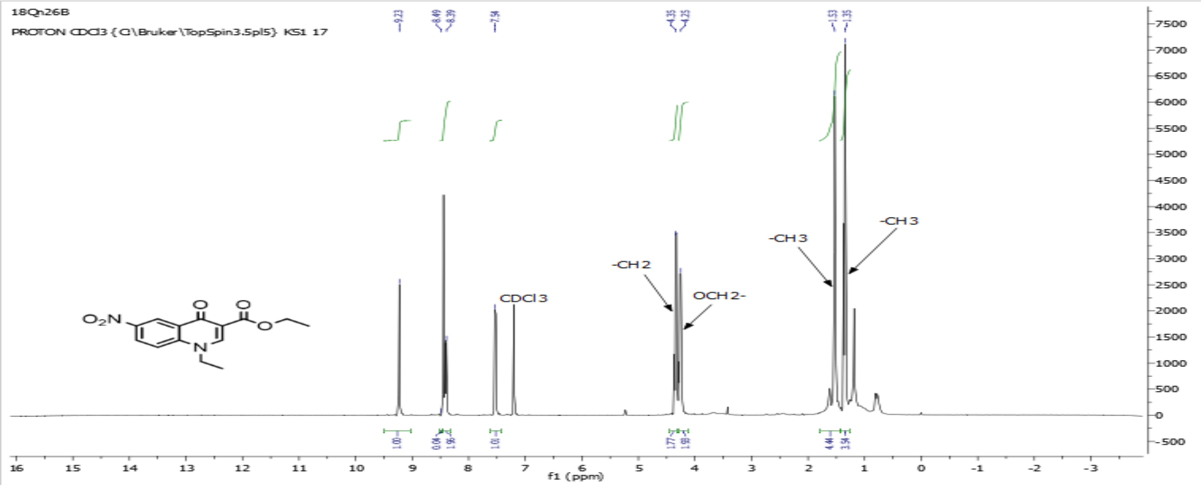
18Qn26B
PROTON CDCl3 {C:\Bruker\TopSpin3.5pl5} KS1 17
CDCl3
-CH2
OCH2-
-CH3
-CH3
f1 (ppm)

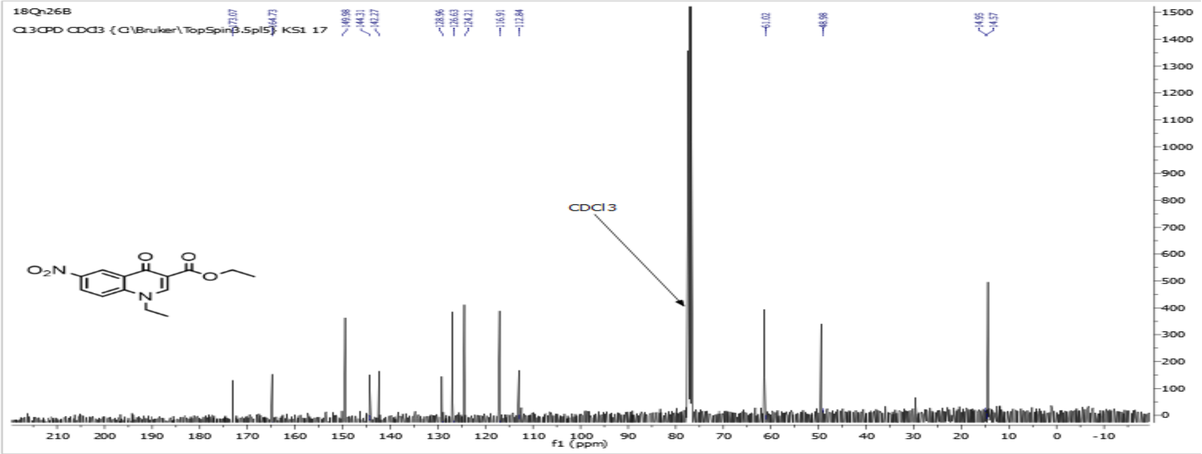
18Qn26B
C13CPD CDCl3 {C:\Bruker\TopSpin3.5pl5} KS1 17
CDCl3
f1 (ppm)

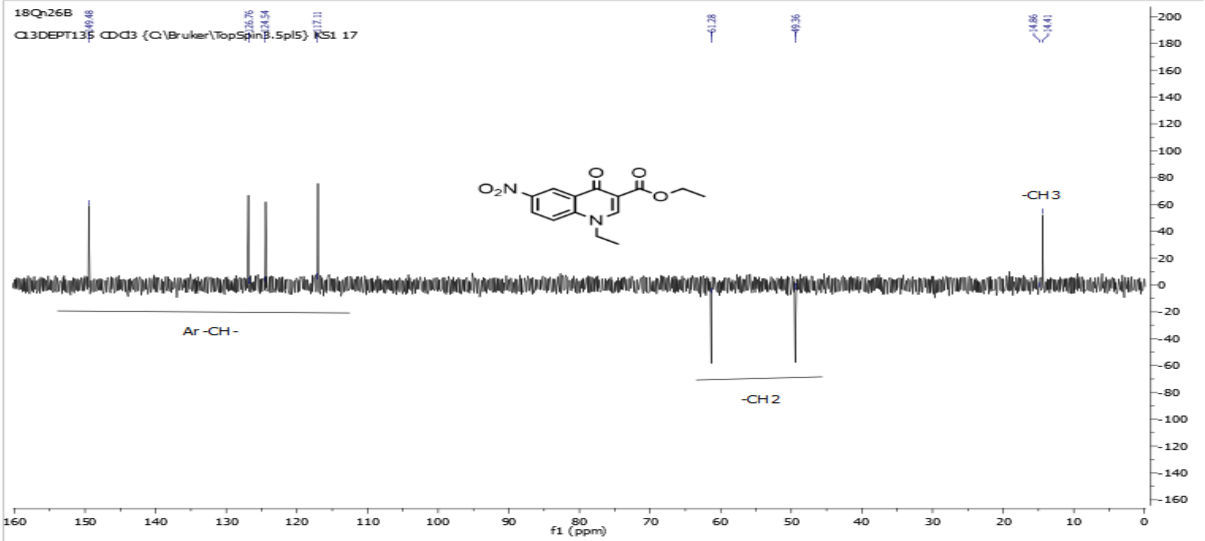
18Qn26B
C13DEPT135 CDCl3 {C:\Bruker\TopSpin3.5pl5} KS1 17
-CH3
Ar -CH-
-CH2
f1 (ppm)

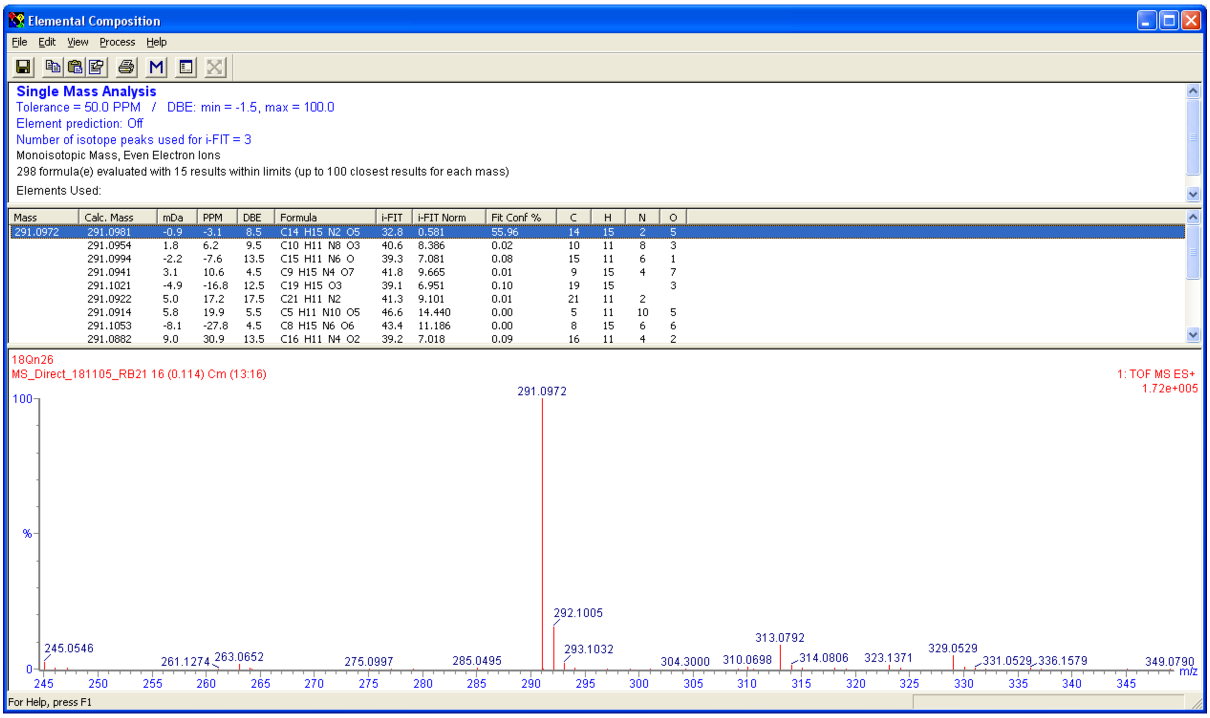

Dose-response against Mtb

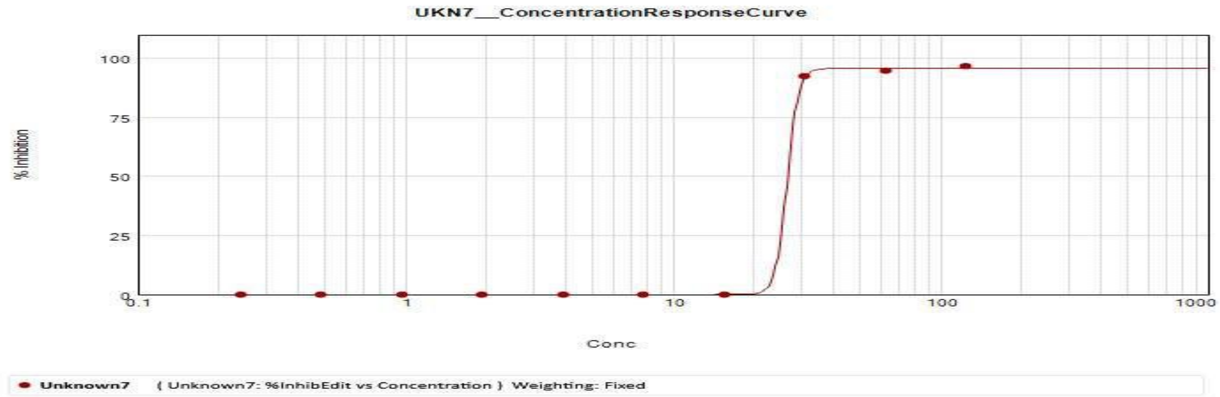

Curve Fit Results

Curve Fit : 4-Parameter $y = D + \frac{A - D}{1 + \left(\frac{x}{C}\right)^B}$

	Parameter	Estimated Value	Std. Error	Confidence Interval
Unknown7 R^2 = 1.000 EC50 = 26.65	A	-2.38e-4	0.232	[-0.568, 0.567]
	B	20.53	531.3	[-1280, 1321]
	C	26.65	109.8	[-242.1, 295.4]
	D	95.78	0.402	[94.80, 96.76]

Compound 12

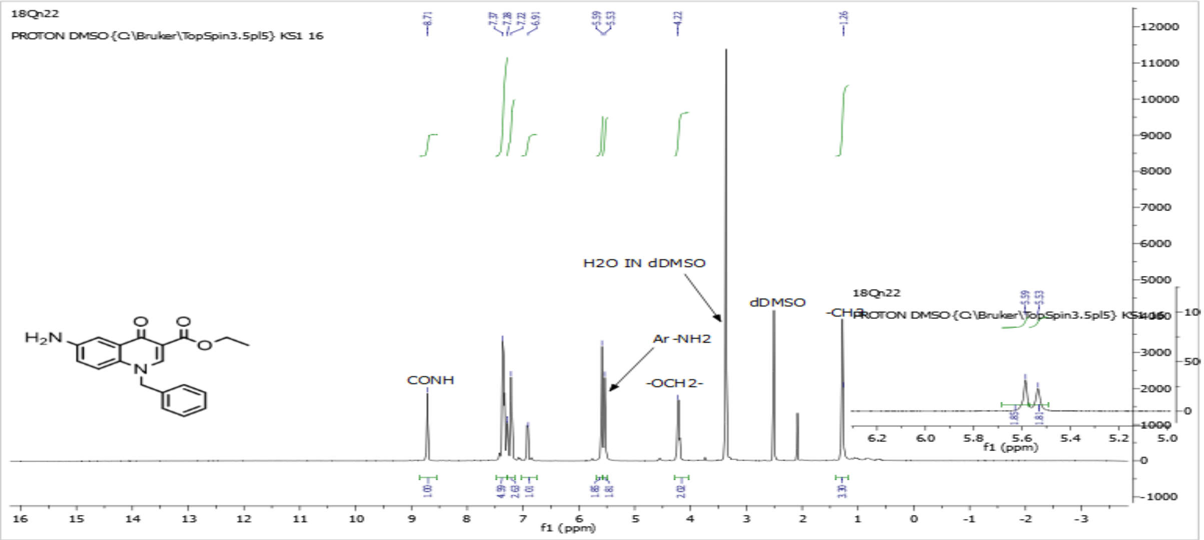
18Qn22
PROTON DMSO {C:\Bruker\TopSpin3.5pl5} KS1 16
H2O IN dDMSO
dDMSO
-CH3
Ar-NH2
CONH
-OCH2-
f1 (ppm)

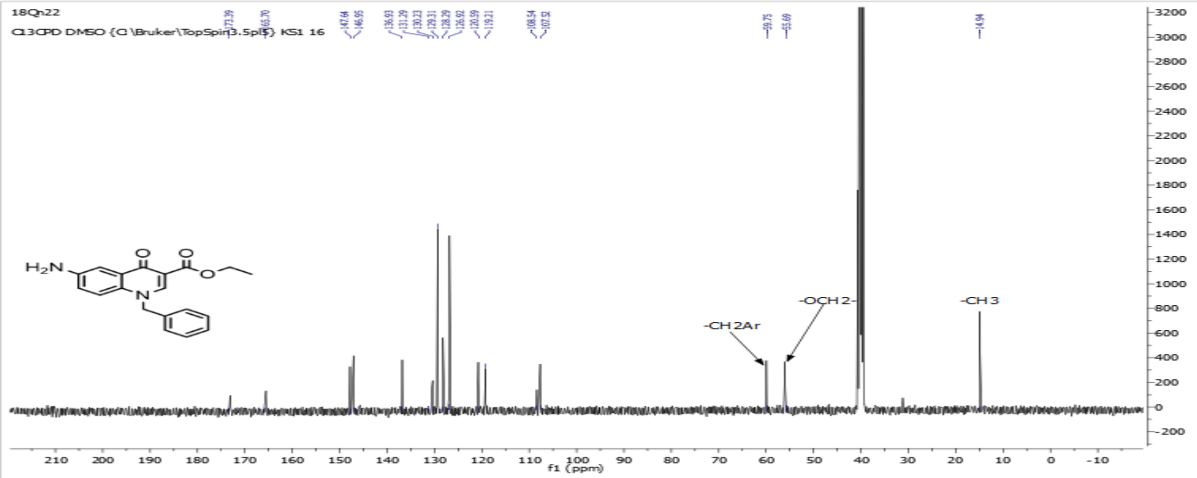
18Qn22
C13CPD DMSO {C:\Bruker\TopSpin3.5pl5} KS1 16
-OCH2-
-CH3
-CH2Ar
f1 (ppm)

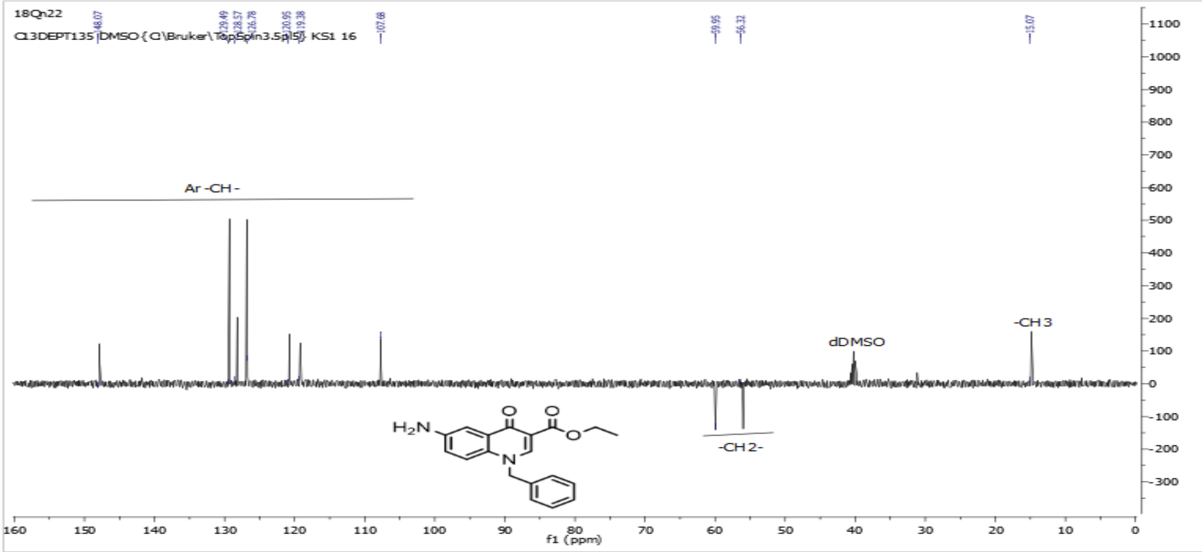
18Qn22
C13DEPT135 DMSO {C:\Bruker\TopSpin3.5pl5} KS1 16
Ar-CH-
dDMSO
-CH3
-CH2-
f1 (ppm)

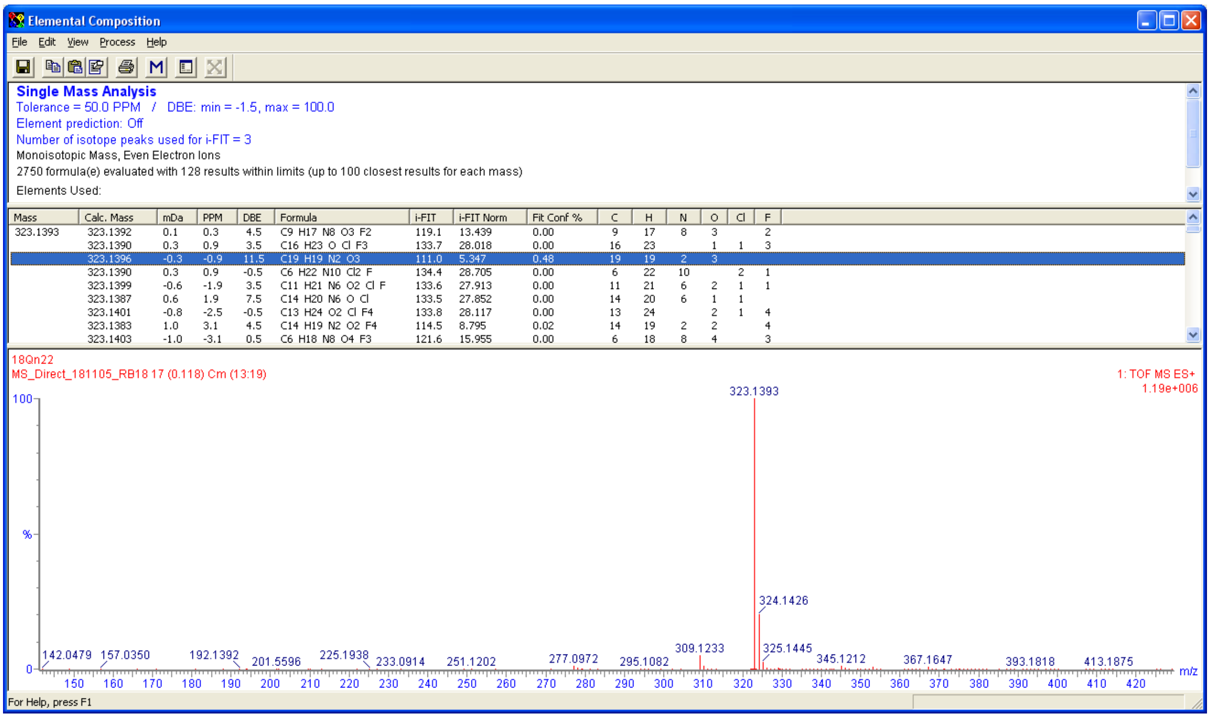

Elemental Composition

File Edit View Process Help

Single Mass Analysis
Tolerance = 50.0 PPM / DBE: min = -1.5, max = 100.0
Element prediction: Off
Number of isotope peaks used for i-FIT = 3
Monoisotopic Mass, Even Electron Ions
2750 formula(e) evaluated with 128 results within limits (up to 100 closest results for each mass)
Elements Used:

Mass	Calc. Mass	mDa	PPM	DBE	Formula	i-FIT	i-FIT Norm	Fit Conf %	C	H	N	O	Cl	F
323.1393	323.1392	0.1	0.3	4.5	C9 H17 N8 O3 F2	119.1	13.439	0.00	9	17	8	3		2
	323.1390	0.3	0.9	3.5	C16 H23 O Cl F3	133.7	28.018	0.00	16	23		1	1	3
	323.1396	-0.3	-0.9	11.5	C19 H19 N2 O3	111.0	5.347	0.48	19	19	2	3		
	323.1390	0.3	0.9	-0.5	C6 H22 N10 Cl2 F	134.4	28.705	0.00	6	22	10		2	1
	323.1399	-0.6	-1.9	3.5	C11 H21 N6 O2 Cl F	133.6	27.913	0.00	11	21	6	2	1	1
	323.1387	0.6	1.9	7.5	C14 H20 N6 O Cl	133.5	27.852	0.00	14	20	6	1	1	
	323.1401	-0.8	-2.5	-0.5	C13 H24 O2 Cl F4	133.8	28.117	0.00	13	24		2	1	4
	323.1383	1.0	3.1	4.5	C14 H19 N2 O2 F4	114.5	8.795	0.02	14	19	2	2		4
	323.1403	-1.0	-3.1	0.5	C6 H18 N8 O4 F3	121.6	15.955	0.00	6	18	8	4		3

Dose-response against Mtb

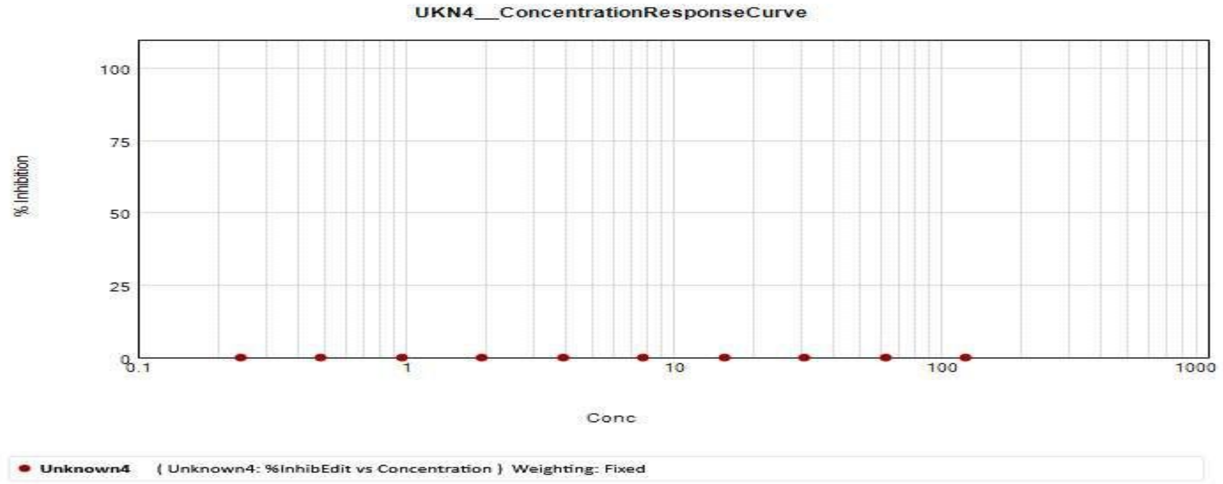

Curve Fit Results ▲

Curve Fit : 4-Parameter $y = D + \frac{A - D}{1 + \left(\frac{x}{C}\right)^B}$

	Parameter	Estimated Value	Std. Error	Confidence Interval
**Unknown4**	A	No fit		
R^2 =	B	No fit		
EC50 =	C	No fit		
	D	No fit		

Compound 13

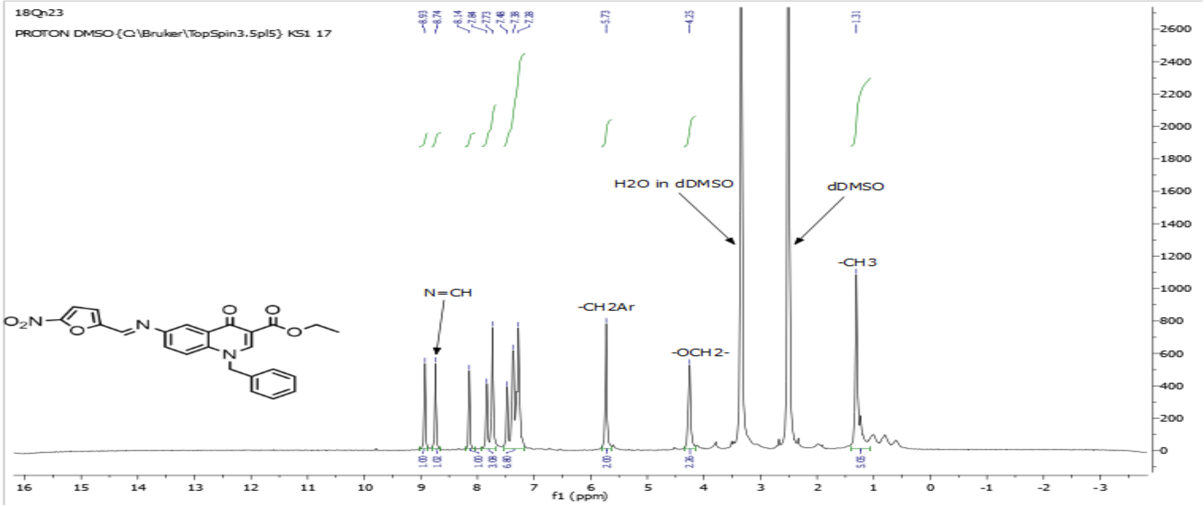

18Qn23
PROTON DMSO {C:\Bruker\TopSpin3.5pl5} KS1 17
8.93
8.74
8.14
7.84
7.73
7.48
7.38
7.28
5.73
4.25
1.31
H2O in dDMSO
dDMSO
-CH3
N=CH
-CH2Ar
-OCH2-
f1 (ppm)

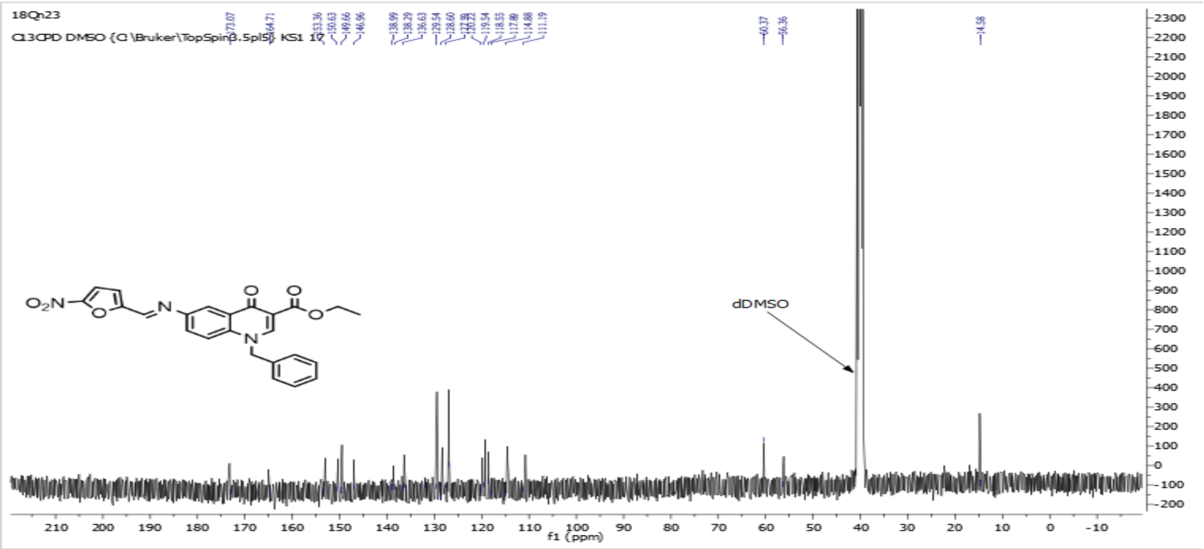

18Qn23
C13CPD DMSO {C:\Bruker\TopSpin3.5pl5} KS1 17
173.07
164.71
60.37
56.36
14.58
dDMSO
f1 (ppm)

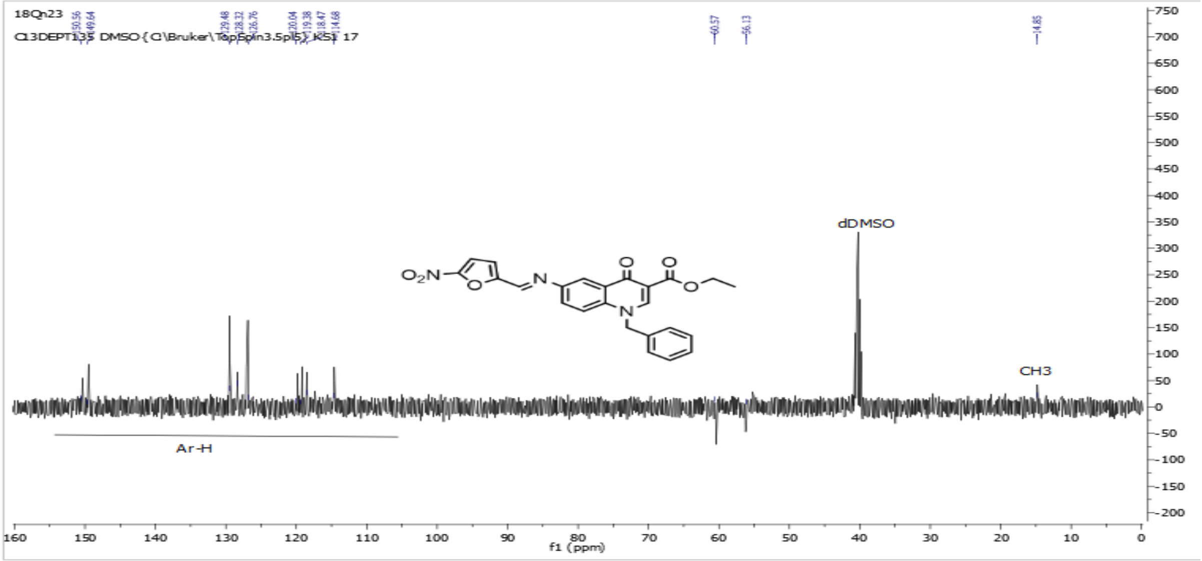

18Qn23
C13DEPT135 DMSO {C:\Bruker\TopSpin3.5pl5} KS1 17
60.57
56.13
14.85
dDMSO
CH3
Ar-H
f1 (ppm)

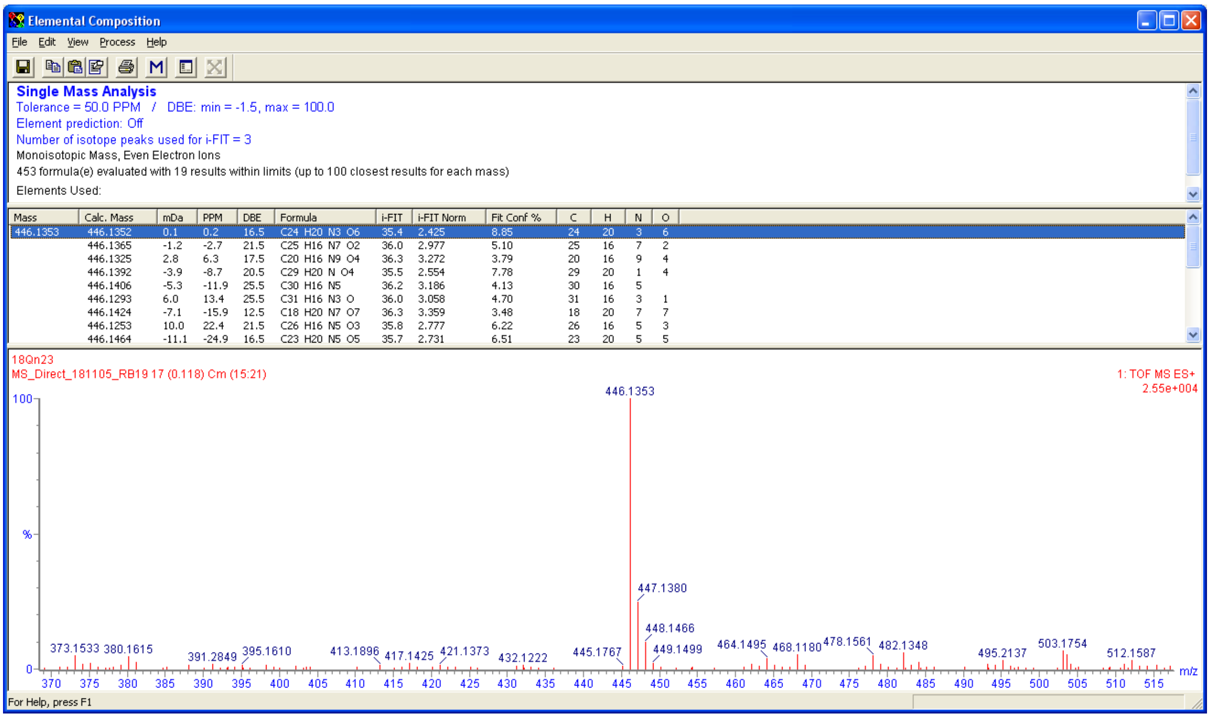

Dose-response against Mtb

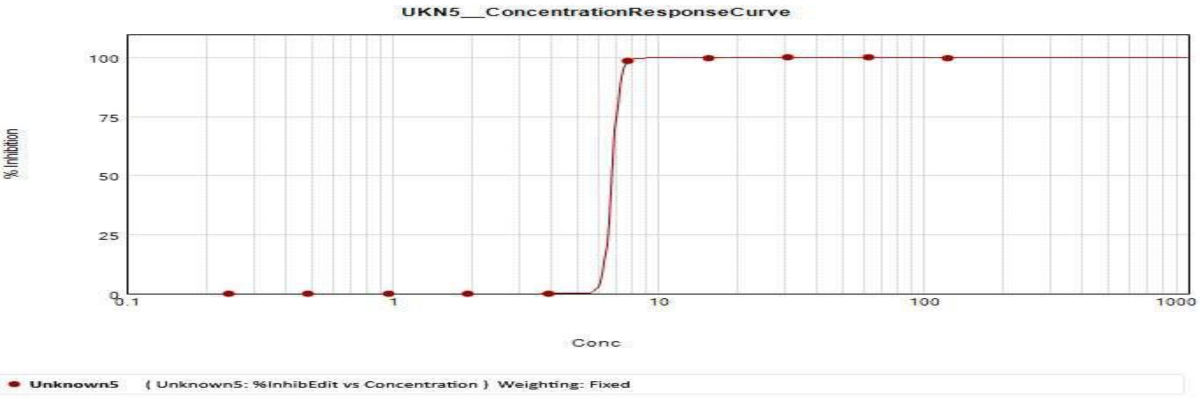

Curve Fit Results ▲

Curve Fit : 4-Parameter $y = D + \frac{A - D}{1 + \left(\frac{x}{C}\right)^B}$

	Parameter	Estimated Value	Std. Error	Confidence Interval
Unknown5<br>R^2 = 1.000<br>EC50 = 6.725	A	-2.69e-6	0.018	[-0.045, 0.045]
	B	29.12	4426	[-1.08e+4, 1.09e+4]
	C	6.725	153.2	[-368.1, 381.5]
	D	99.95	0.018	[99.90, 99.99]

Compound 14

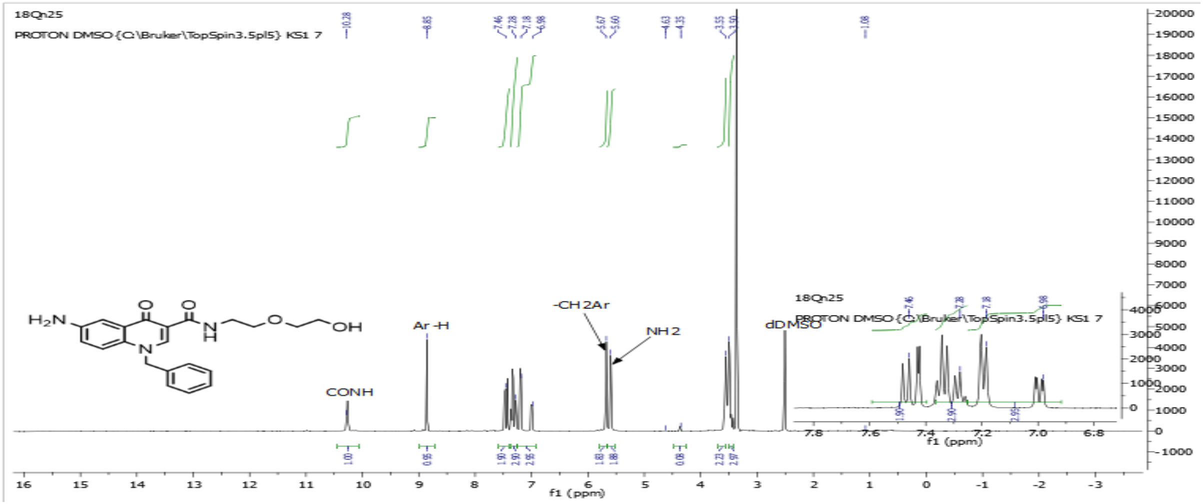
18Qn25
PROTON DMSO {C:\Bruker\TopSpin3.5pl5} KS1 7
CONH
Ar-H
-CH2Ar
NH2
dDMSO
f1 (ppm)

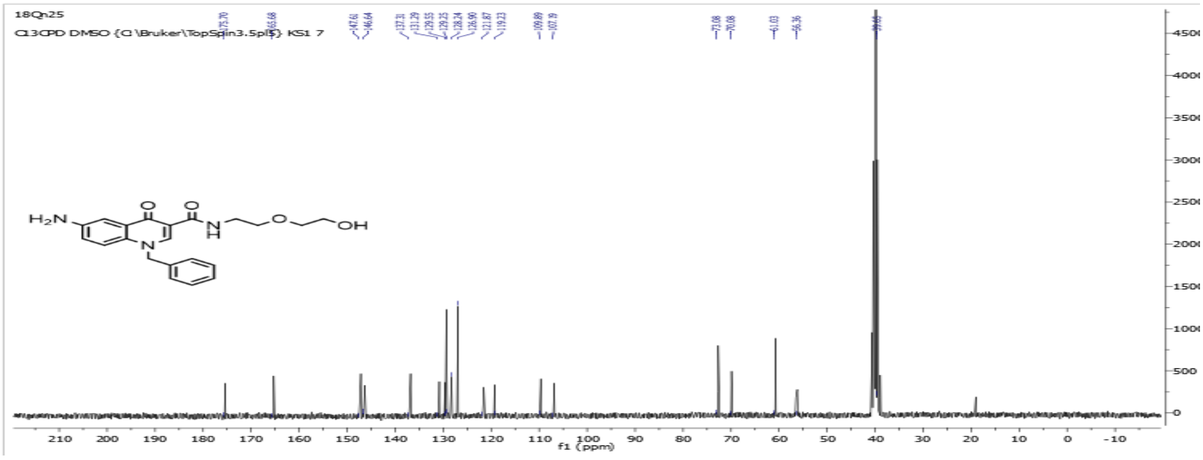
18Qn25
C13CPD DMSO {C:\Bruker\TopSpin3.5pl5} KS1 7
f1 (ppm)

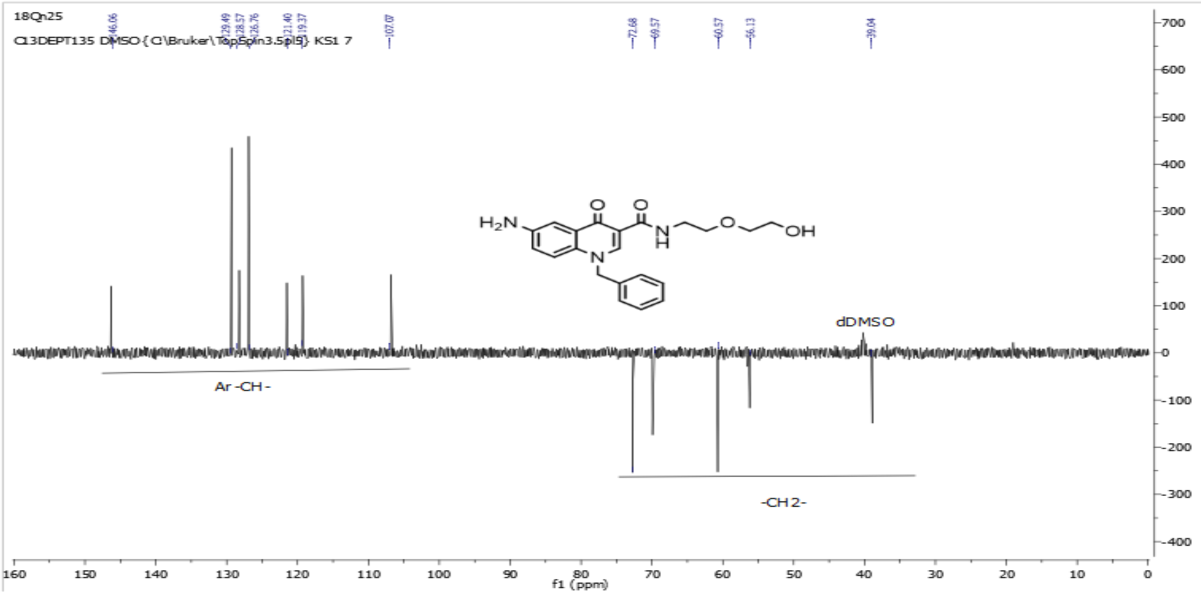
18Qn25
C13DEPT135 DMSO {C:\Bruker\TopSpin3.5pl5} KS1 7
dDMSO
Ar-CH-
-CH2-
f1 (ppm)

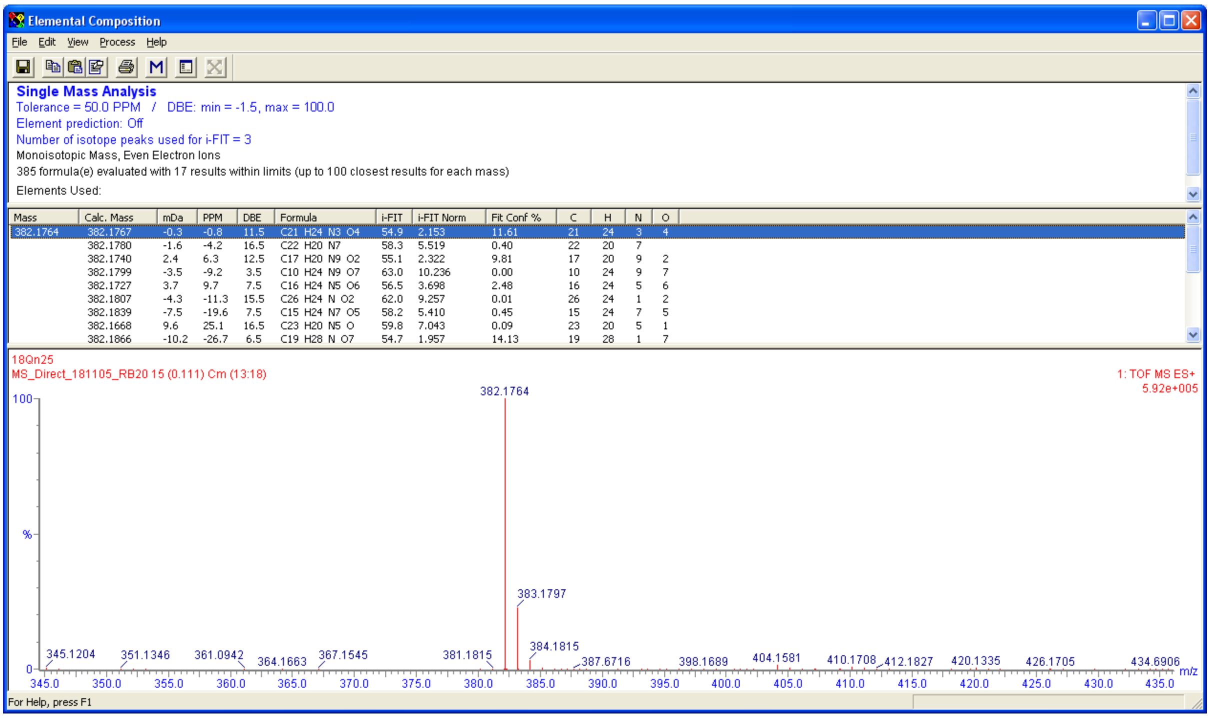

Dose-response against Mtb

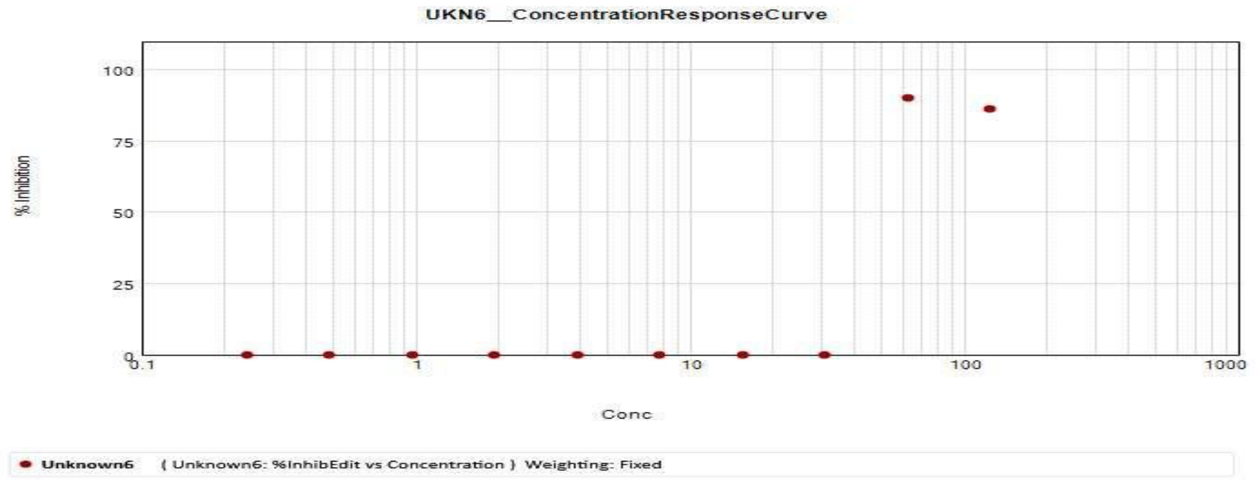

Curve Fit Results ▲

Curve Fit : 4-Parameter $y = D + \frac{A - D}{1 + \left(\frac{x}{C}\right)^B}$

	Parameter	Estimated Value	Std. Error	Confidence Interval
**Unknown6**	A	No fit		
R^2 =	B	No fit		
EC50 =	C	No fit		
	D	No fit		

Compound 16

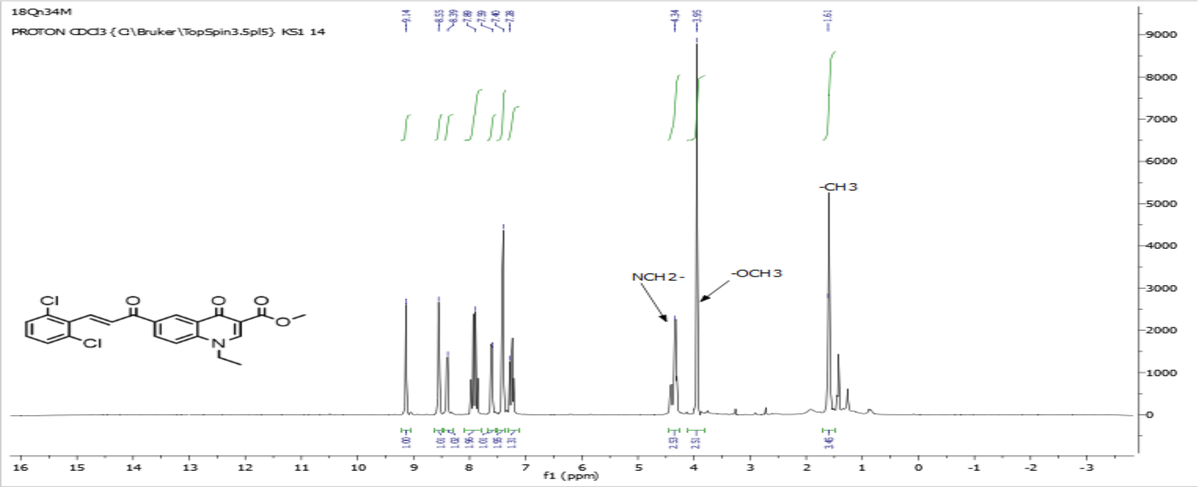

18Qn34M
PROTON CDCl3 {C:\Bruker\TopSpin3.5pl5} KS1 14
-CH3
NCH2-
-OCH3
f1 (ppm)

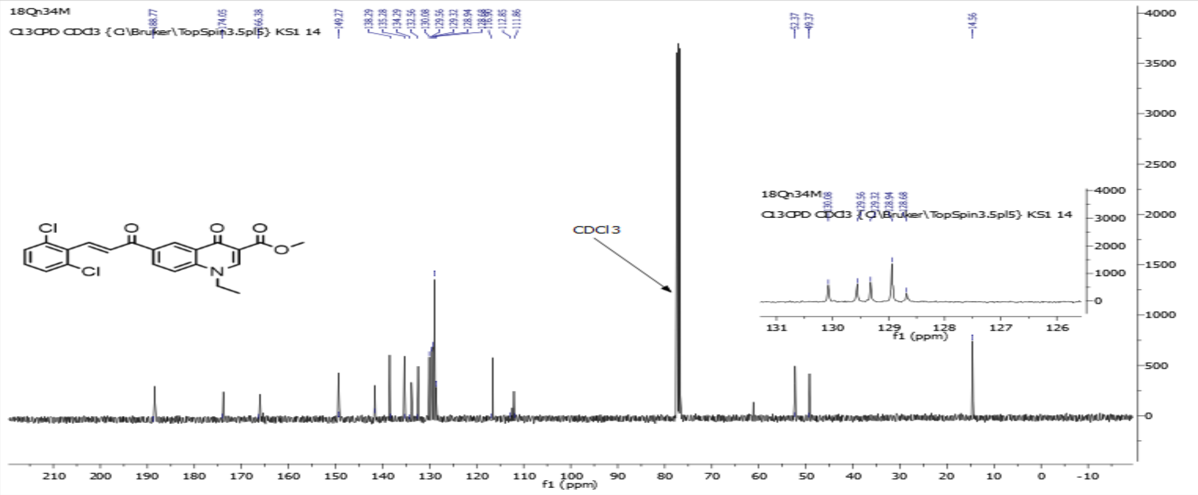

18Qn34M
C13CPD CDCl3 {C:\Bruker\TopSpin3.5pl5} KS1 14
CDCl3
f1 (ppm)

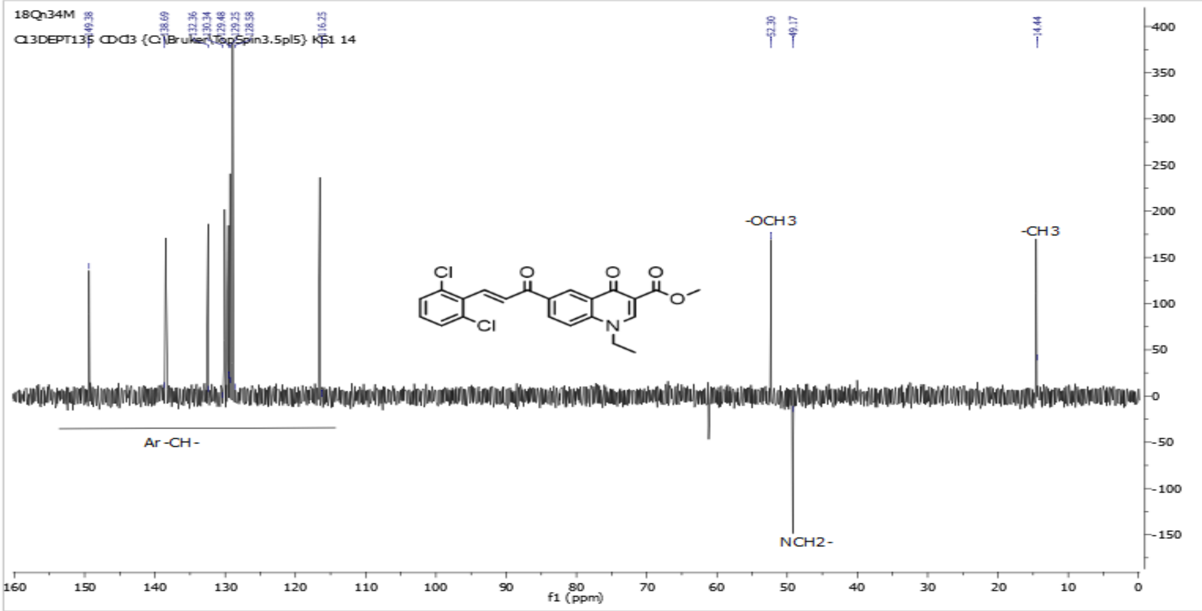

18Qn34M
C13DEPT135 CDCl3 {C:\Bruker\TopSpin3.5pl5} KS1 14
-OCH3
-CH3
NCH2-
Ar -CH-
f1 (ppm)

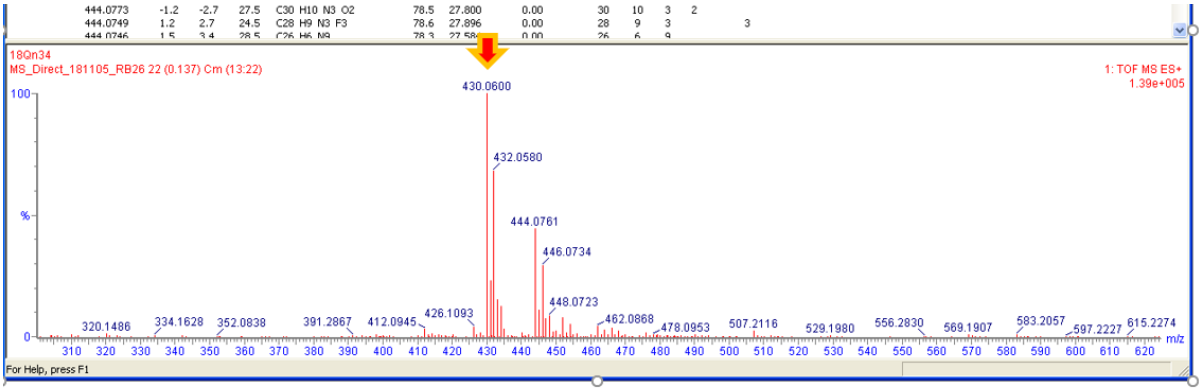

Dose-response against Mtb

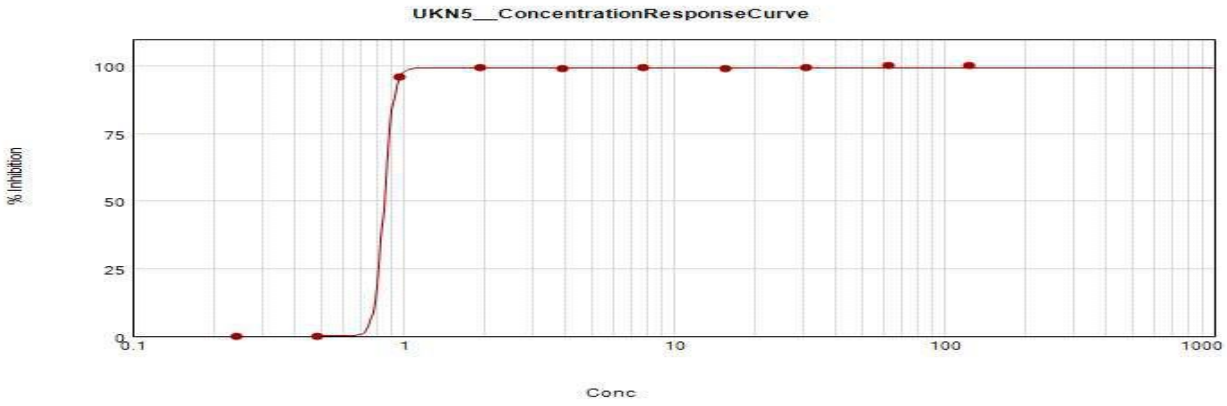

● Unknown5 { Unknown5: %InhibEdit vs Concentration } Weighting: Fixed

Curve Fit Results ▲

Curve Fit : 4-Parameter $y = D + \frac{A - D}{1 + \left(\frac{x}{C}\right)^B}$

	Parameter	Estimated Value	Std. Error	Confidence Interval
**Unknown5**<br>R^2 = 1.000<br>EC50 = 0.851	A	-6.48e-5	0.408	[-0.997, 0.997]
	B	24.39	6411	[-1.57e+4, 1.57e+4]
	C	0.851	30.78	[-74.47, 76.17]
	D	99.40	0.154	[99.02, 99.77]
